# A two-step approach to achieve secondary amide transamidation enabled by nickel catalysis

**DOI:** 10.1038/ncomms11554

**Published:** 2016-05-20

**Authors:** Emma L. Baker, Michael M. Yamano, Yujing Zhou, Sarah M. Anthony, Neil K. Garg

**Affiliations:** 1 Department of Chemistry and Biochemistry, University of California, Los Angeles, California 90095, USA

## Abstract

A long-standing challenge in synthetic chemistry is the development of the transamidation reaction. This process, which involves the conversion of one amide to another, is typically plagued by unfavourable kinetic and thermodynamic factors. Although some advances have been made with regard to the transamidation of primary amide substrates, secondary amide transamidation has remained elusive. Here we present a simple two-step approach that allows for the elusive overall transformation to take place using non-precious metal catalysis. The methodology proceeds under exceptionally mild reaction conditions and is tolerant of amino-acid-derived nucleophiles. In addition to overcoming the classic problem of secondary amide transamidation, our studies expand the growing repertoire of new transformations mediated by base metal catalysis.

## Abstract (web summary)

Transamidation reactions are kinetically and thermodynamically challenging because of the stability of the amide starting materials. Here, the authors show a two-step process—activation of a secondary amide, followed by nickel-catalysed C–N bond cleavage—that allows mild and high yielding transamidation.

## Graphical abstract

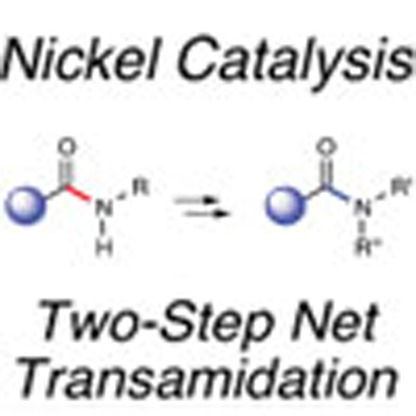

Within the realm of chemical synthesis, our ability to interconvert functional groups remains an important endeavour with tremendous impact^1^,^2^. One particularly interesting transformation is the transamidation reaction (Fig. 1a)^3^,^4^,^5^. In addition to being conceptually curious, the reaction could serve as a practical means to prepare amides. Transamidation, however, has proven difficult to achieve both synthetically and enzymatically^6^. Primary amide transamidation is the most common^7^,^8^. In contrast, the transamidation of secondary amides, which are ubiquitous in peptides, has remained an unsolved problem. Over the past few decades, only two methodological advances have been described: Bertrand's usage of excess AlCl_3_ to promote the transamidation of several unfunctionalized substrates, described in 1994 (ref. 9), and the 2003 report by Gelman and Stahl of a dimeric aluminium complex to promote secondary amide transamidation, which yields equilibrium mixtures^10^. A simple and efficient method to achieve secondary amide transamidation has eluded discovery.

Two main challenges must be overcome to achieve the transamidation of secondary amides. First, amides are notoriously stable because of resonance effects^11^,^12^. One must therefore surmount the high kinetic barrier for acyl C–N bond cleavage. The other difficulty pertains to thermodynamics, as the conversion of a secondary amide to another amide is effectively a thermoneutral process, aside from specialized cases. Indeed, the state-of-the-art methodology to achieve the transamidation of secondary amides gives equilibrium mixtures of substrate and product amides^10^,^12^.

To overcome the kinetic and thermodynamic challenges associated with secondary amide transamidation, we pursued the two-step approach outlined in Fig. 1b. It was envisioned that substrate **1** would first undergo *N*-functionalization with an electron-withdrawing group as a means to weaken the amide C–N bond^13^,^14^. From ‘activated' species **5**, oxidative addition using an appropriate nickel^15^,^16^,^17^ catalyst would give acyl metal species **6**. This step would rely on the recently disclosed nickel-catalysed amide bond cleavage that has only seen limited use in esterification and the Suzuki–Miyaura coupling.^18^,^19^ Interception of this intermediate with primary or secondary amines **2** would furnish the desired product **3**. The reaction would be driven thermodynamically by the liberation of the *N*-functionalized amine **7**, which would be less nucleophilic than amine **2** undergoing transamidation.

In this manuscript, we demonstrate the success of our two-step approach to achieve the net transamidation of secondary amides. The methodology utilizes non-precious metal catalysis and proceeds under exceptionally mild reaction conditions. Moreover, the chemistry is tolerant of amino-acid-derived nucleophiles. These studies provide the most general solution to the classic challenge of secondary amide transamidation available to-date.

## Results

### Discovery of the transamidation reaction

**Figure 2:**
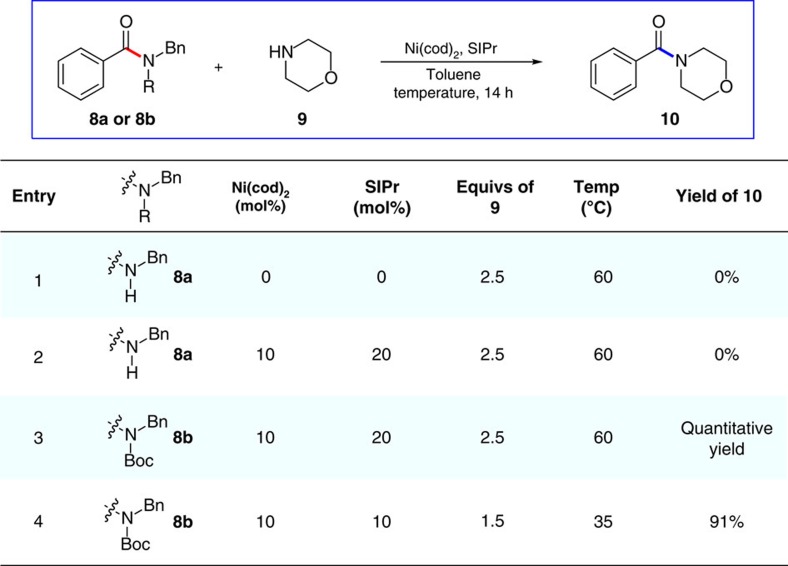
Key experiments and reaction discovery. Failed reaction using substrate **8a** in the absence of catalyst/ligands or activating group (entries 1 and 2), initial promising result (entry 3) and optimized reaction conditions (entry 4). For entries 1–3, yields shown were determined by ^1^H NMR analysis with an internal standard. For entry 4, the yield shown reflects the average of two isolation experiments.

Our studies commenced by surveying the reaction of simple benzamides and morpholine using nickel catalysis (see Supplementary Table 1 for full details). A small sampling of control experiments and key results are shown in Fig. 2. As expected, the attempted transamidation of secondary amide **8a** with morpholine (**9**) fails in the absence (entry 1) or presence of nickel/SIPr (entry 2). However, after Boc activation of the substrate, we were delighted to find that **8b** underwent the nickel-catalysed reaction with **9** to deliver morpholino amide **10** quantitatively (entry 3). Of note, no reaction takes place if the nickel and SIPr are excluded from the reaction, thus highlighting the unique ability of nickel catalysis to facilitate the net transamidation reaction. We also examined less forcing conditions. It was ultimately found that exposure of **8b** to 1.5 equivalents of amine **9** at 35 °C furnished amide **10** in 91% isolated yield (entry 4). These exceptionally mild reaction conditions were deemed suitable for exploration of the substrate scope.

### Scope of the methodology

**Figure 3:**
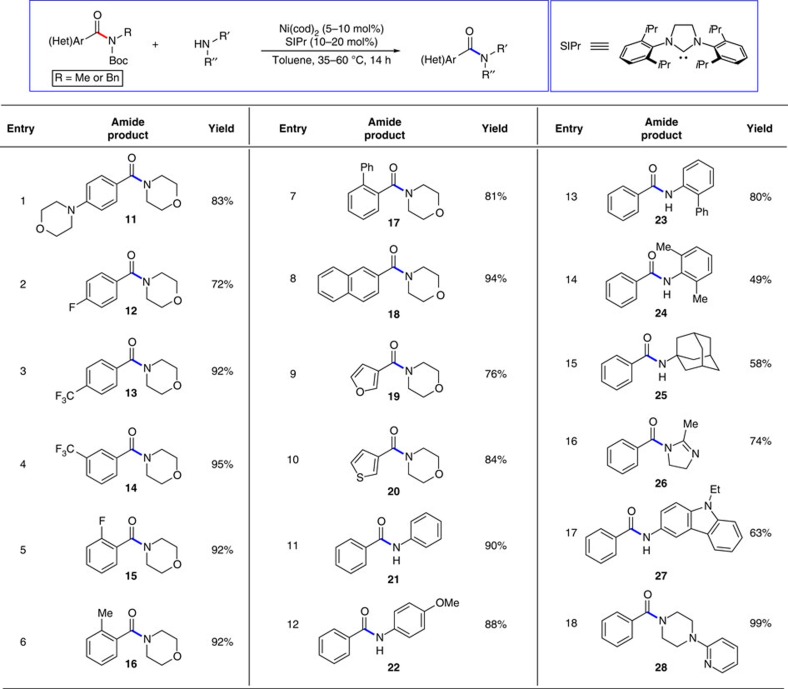
Scope of methodology. The scope of the transamidation methodology was evaluated with respect to the Boc-activated amide substrate (entries 1–10), and with respect to the amine nucleophile using **8b** (entries 11–18). Reactions were carried out using Ni(cod)_2_ (5–10 mol%), SIPr (10–20 mol%), substrate (1.00 equiv), amine (1.5–2.5 equiv) and toluene (1.0 M) at 35–60 °C for 14 h. Yields shown reflect the average of two isolation experiments.

A variety of readily accessible Boc-activated secondary amides were examined. As shown in Fig. 3, considerable variation is tolerated with regard to the benzamide substrate and the amine nucleophile. Substrates bearing electron-donating or -withdrawing groups can be utilized, as shown by the formation of **11**–**15** (entries 1–5). Of note, CF_3_ and F substituents, often encountered in medicinally relevant scaffolds^20^,^21^, posed no difficulty (entries 2–5). Another interesting aspect of the reaction scope is that ortho substitution is well tolerated (entries 5–7), suggesting that the methodology does not suffer from steric hindrance. The extended aromatic naphthyl unit could also be employed to give **18** (entry 8), in addition to the furan and thiophene heterocycles to furnish **19** and **20**, respectively (entries 9 and 10). Substrates derived from aliphatic amides do not undergo the nickel-catalysed transamidation under our typical reaction conditions, which may ultimately provide opportunities for achieving site-selective reactions of polyamide substrates. With regard to the amine reactant, various alternatives to morpholine can be employed. For example, anilines may be utilized, as shown by the formation of secondary amides **21**–**24** (entries 11–14). Moreover, the use of adamantyl-1-amine furnished amide **25** in 58% yield (entry 15). The formation of **24** and **25** (entries 14 and 15) again highlights the tolerance of this methodology towards steric demands. Finally, several heterocyclic amine nucleophiles were tested and found to undergo the nickel-catalysed reaction. The successful use of an amidine, carbazole and pyridine-appended piperazine to provide **26**–**28**, respectively (entries 16–18), demonstrates the tolerance of the nickel catalyst system towards multiple basic heteroatoms.

## Discussion

**Figure 4:**
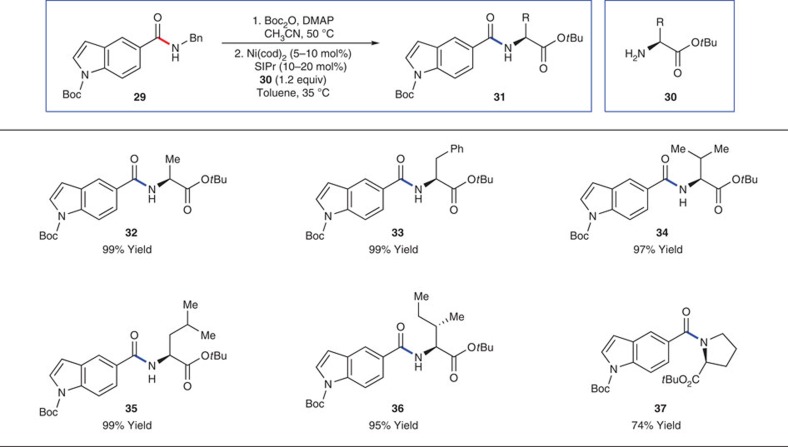
The two-step approach for transamidation with amino-acid derivatives. The scope of the transamidation was evaluated with various amino-acid derivatives to give products **32**–**37**. Amide **29** was first Boc-activated with Boc_2_O (3.0 equiv), 4-(dimethylamino)pyridine (30 mol%) and CH_3_CN (0.20 M) at 50 °C for 19 h. The transamidation reactions were then carried out using Ni(cod)_2_ (5–10 mol%), SIPr (10–20 mol%), substrate (1.00 equiv), amine (1.2 equiv) and toluene (1.0 M) at 35 °C for 14 h. Yields shown reflect the average of two isolation experiments.

The results above demonstrate the feasibility of the second part of our two-step reaction design. As a critical final evaluation of this chemistry, we performed the net transamidation of a secondary amide substrate using a variety of amino-acid-derived nucleophiles (Fig. 4). Thus, indolylamide **29** readily underwent Boc activation in quantitative yield under standard conditions. Next, exposure of this intermediate to a variety of optically pure amino esters gave the corresponding secondary amides in excellent yields over the two-step process. Nucleophiles derived from alanine, phenylalanine, valine, leucine, isoleucine and proline could all be utilized, leading to secondary amides **32**–**37**. The mild nature of this two-step process should not be overlooked. In all cases, the *N*-Boc of the indole and the ester of the amine nucleophile were not disturbed. Moreover, each product was obtained in >99% ee, suggesting that no appreciable loss of stereochemical fidelity occurred. This is notable since each nucleophile and product contains an epimerizable stereocentre.

In summary, we have developed a two-step approach to effect the transamidation of secondary amides. Our strategy relies on a simple Boc-activation step of the secondary amide substrates, followed by an uncommon nickel-catalysed activation of an acyl C–N bond. The methodology proceeds under mild conditions, is tolerant of variation in both reaction partners and proceeds in the presence of basic heteroatoms and several heterocyclic scaffolds. Moreover, ester functional groups and epimerizable stereocentres found in amino-acid derivatives easily withstand the reaction conditions. To test the scalability of this method, a gram-scale coupling of the indole substrate with the phenylalanine amino ester derivative gave the desired transamidated product in excellent yield. Future efforts focused on ligand development are expected to fuel further breakthroughs in reaction discoveries, much like the evolution witnessed in recent decades pertaining to Pd- and Ni-catalysed couplings. Nonetheless, these studies provide the most general solution to the classic challenge of secondary amide transamidation available to-date, while also expanding the growing repertoire of new transformations mediated by base metal catalysis.

## Methods

### General

^1^H NMR (500 MHz), ^13^C NMR (125 MHz) and ^19^F NMR (282 MHz) spectra were recorded on Bruker spectrometers. High resolution mass spectrometry was performed on Thermo Scientific Exactive Mass Spectrometer with DART ED-CUBE. Infrared spectra were recorded on a Perkin-Elmer UATR Two Fourier transform infrared spectrometer. Determination of enantiopurity was carried out on a Mettler Toledo supercritical fluid chromatography using a Daicel ChiralPak OJ-H column. Supplementary Figs 1–46 for the NMR spectra and HPLC chromatograms, Supplementary Table 1 for the optimization of reaction and Supplementary Methods for the characterization data can be found in the Supplementary Information.

### General procedure Ni-catalysed transamidation reaction

A 1-dram vial containing a magnetic stir bar was flame-dried under reduced pressure, and then allowed to cool under N_2_. The vial was charged with substrate **SI-7** (0.200 mmol, 1.0 equiv) and the free-based amino ester (0.240 mmol, 1.2 equiv). The vial was flushed with N_2_, then taken into a glove box and charged with Ni(cod)_2_ (0.010–0.020 mmol, 5–10 mol%) and SIPr (0.020–0.040 mmol, 10–20 mol%). Subsequently, toluene (0.20 ml, 1.0 M) was added. The vial was sealed with a Teflon-lined screw cap, removed from the glove box and stirred at 35 °C for 14 h. After cooling to 23 °C, the mixture was diluted with hexanes (0.5 ml) and filtered over a plug of silica gel (10 ml of ethyl acetate eluent). The volatiles were removed under reduced pressure, and the crude residue was purified by preparative thin-layer chromatography.

### Gram-scale transamidation procedure

A scintillation vial containing a magnetic stir bar was flame-dried under reduced pressure, and then allowed to cool under N_2_. The vial was charged with **SI-7** (2.22 mmol, 1.0 equiv) and the free-based amino ester **SI-28** (2.66 mmol, 1.2 equiv). The vial was flushed with N_2_, then taken into a glove box and charged with Ni(cod)_2_ (0.111 mmol, 5 mol%) and SIPr (0.222 mmol, 10 mol%). Subsequently, toluene (2.22 ml, 1.0 M) was added. The vial was sealed with a Teflon-lined screw cap, removed from the glove box and stirred at 35 °C for 14 h. After cooling to 23 °C, the mixture was diluted with hexanes (5.0 ml) and filtered over a plug of silica gel (100 ml of EtOAc eluent). The volatiles were removed under reduced pressure, and the crude residue was purified via flash chromatography (20:1 hexanes:EtOAc→15:1→5:1) to yield amide product **33** (quant. yield).

## Additional information

**How to cite this article:** Baker, E. L. *et al*. A two-step approach to achieve secondary amide transamidation enabled by nickel catalysis. *Nat. Commun.* 7:11554 doi: 10.1038/ncomms11554 (2016).

## Supplementary Material

Supplementary Information Supplementary Figures 1-46, Supplementary Table 1, Supplementary Methods and Supplementary References

## Figures and Tables

**Figure 1:**
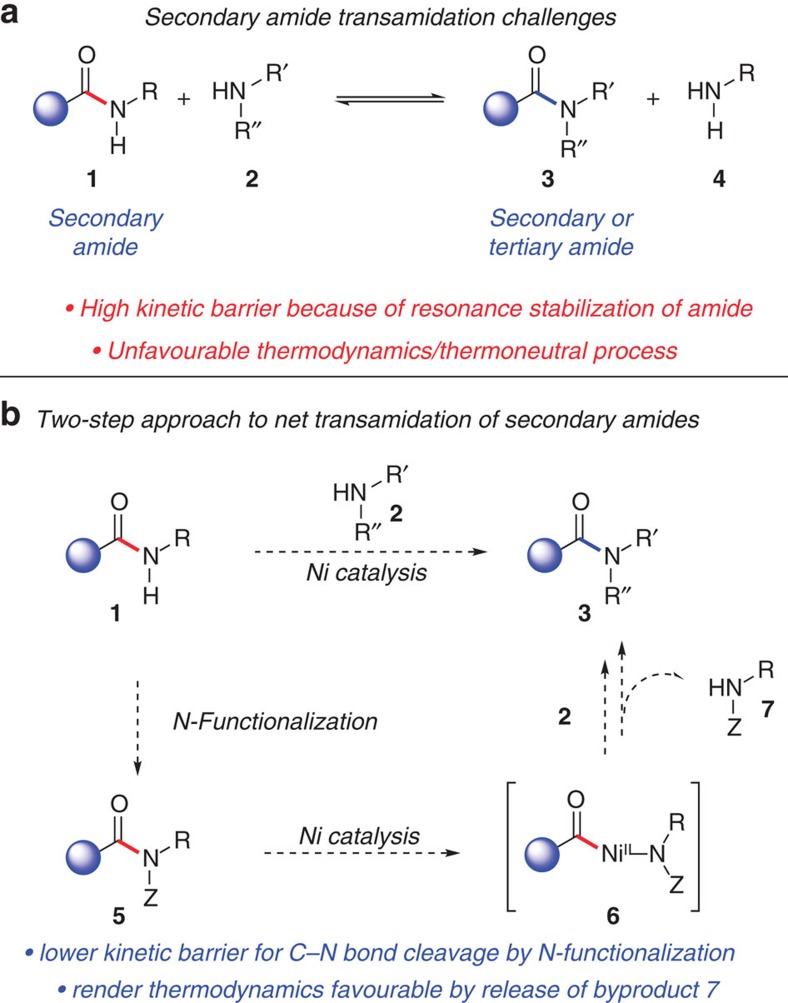
Development of Ni-catalysed secondary amide transamidation. (**a**) An illustration of the challenges currently faced with secondary amide transamidation. (**b**) The two-step approach to achieve net transamidation of secondary amides via *N*-functionalization and C–N bond activation.
