# Supplementary material for: A two-step approach to achieve secondary amide transamidation enabled by nickel catalysis
Source: Nat Commun. 2016 May 20;7:11554. doi: 10.1038/ncomms11554 (PMC4876455; doi:10.1038/ncomms11554)
Supplement: Supplementary Information — Supplementary Figures 1-46, Supplementary Table 1, Supplementary Methods and Supplementary References [file ncomms11554-s1.pdf]

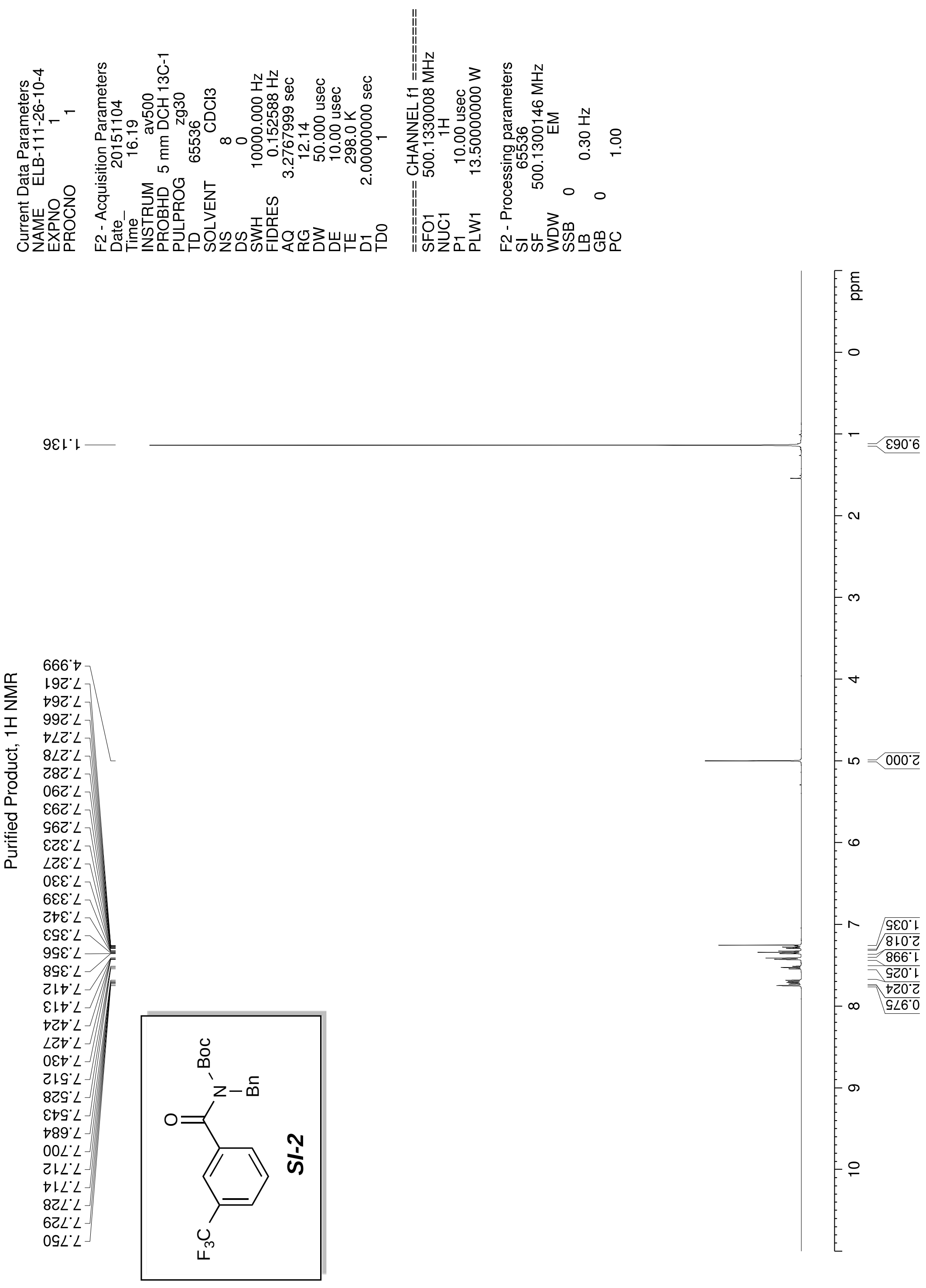


**Supplementary Figure 1.** **$^{1}$H NMR (500 MHz, $CDCl_3$) of SI-2**

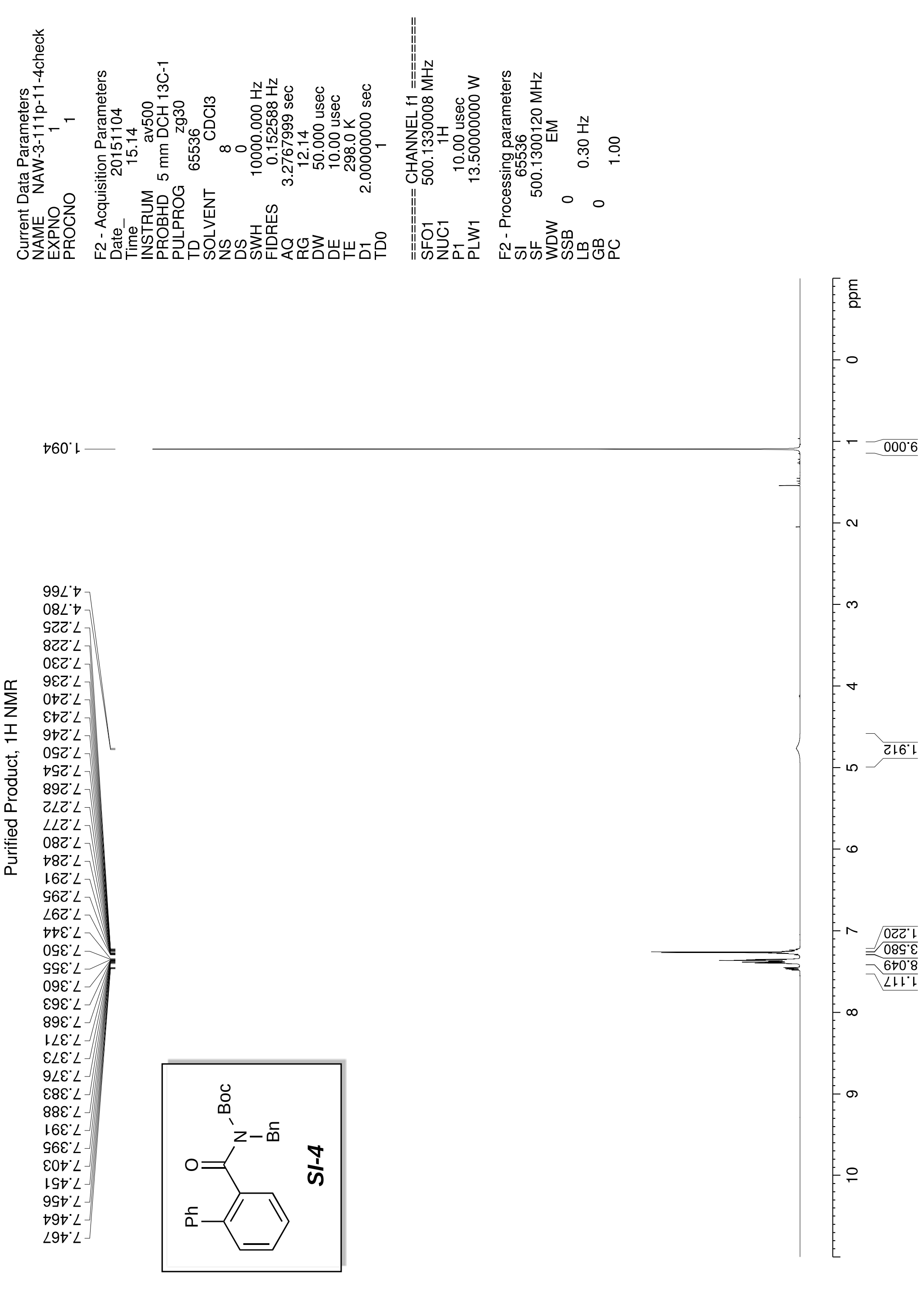


**Supplementary Figure 2. $^{1}$H NMR (500 MHz, $CDCl_3$) of SI-4**

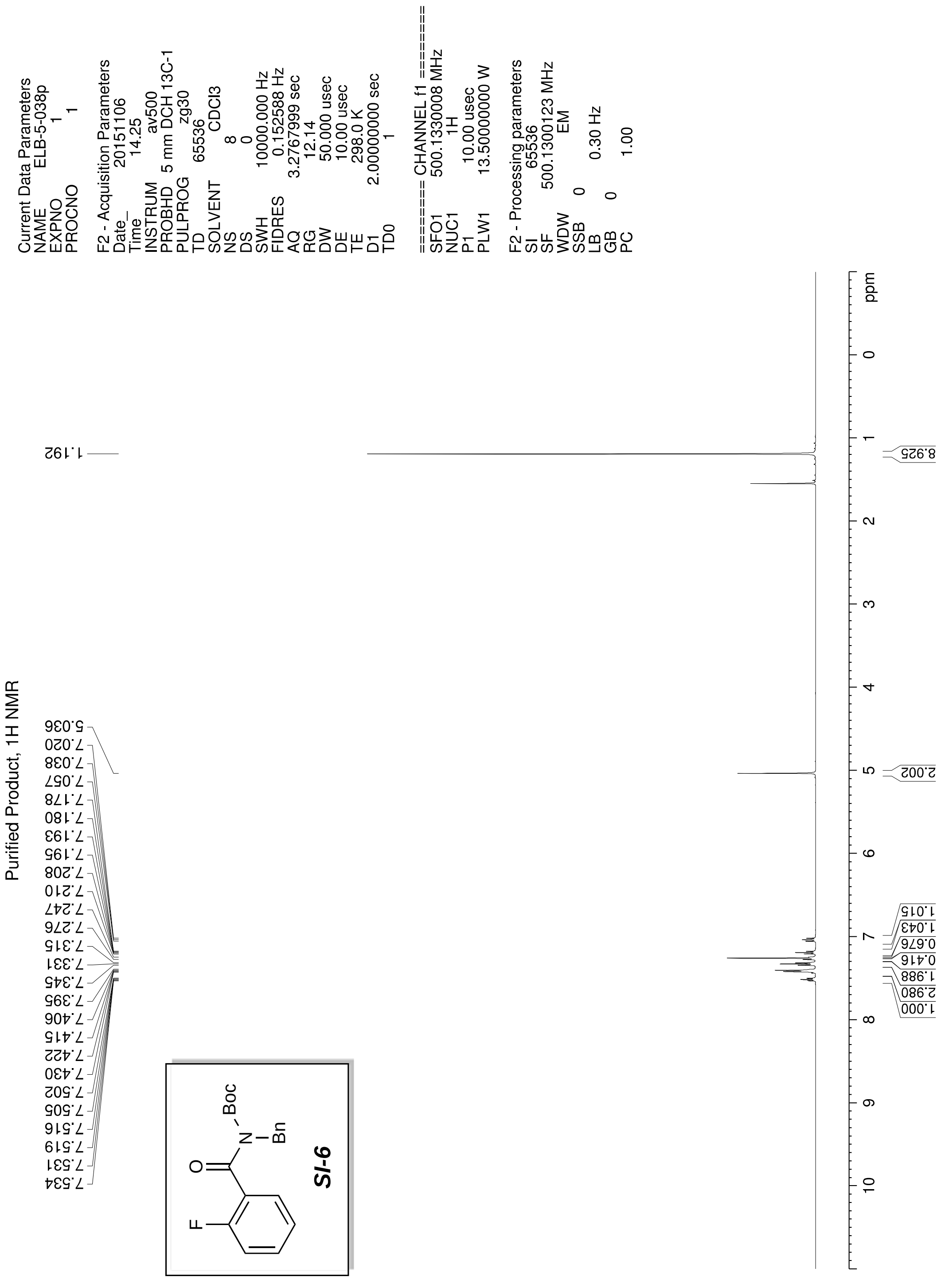


**Supplementary Figure 3.** **$^{1}$H NMR (500 MHz, $CDCl_3$) of SI-6**

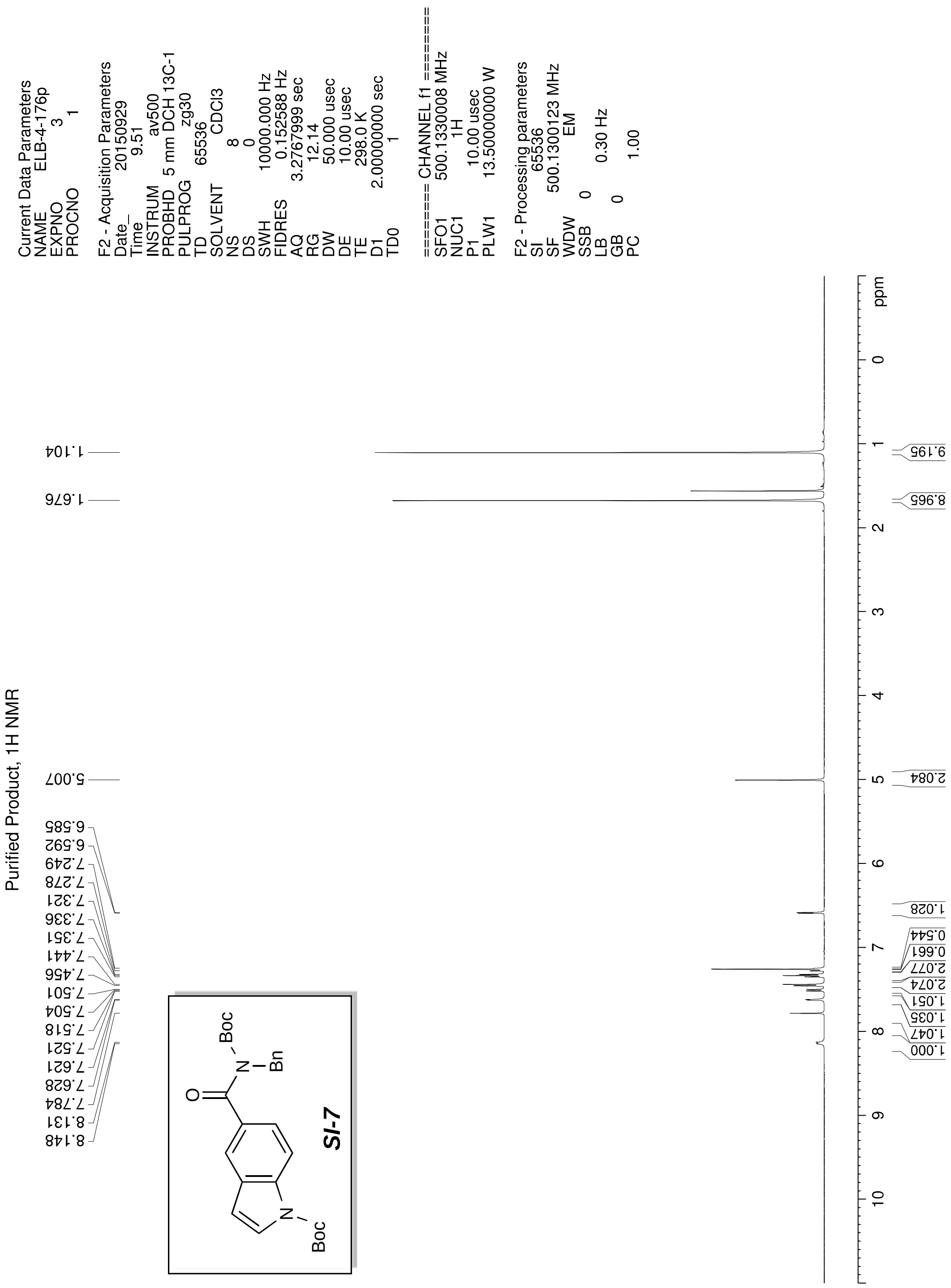


**Supplementary Figure 4. $^{1}$H NMR (500 MHz, $CDCl_3$) of SI-7**

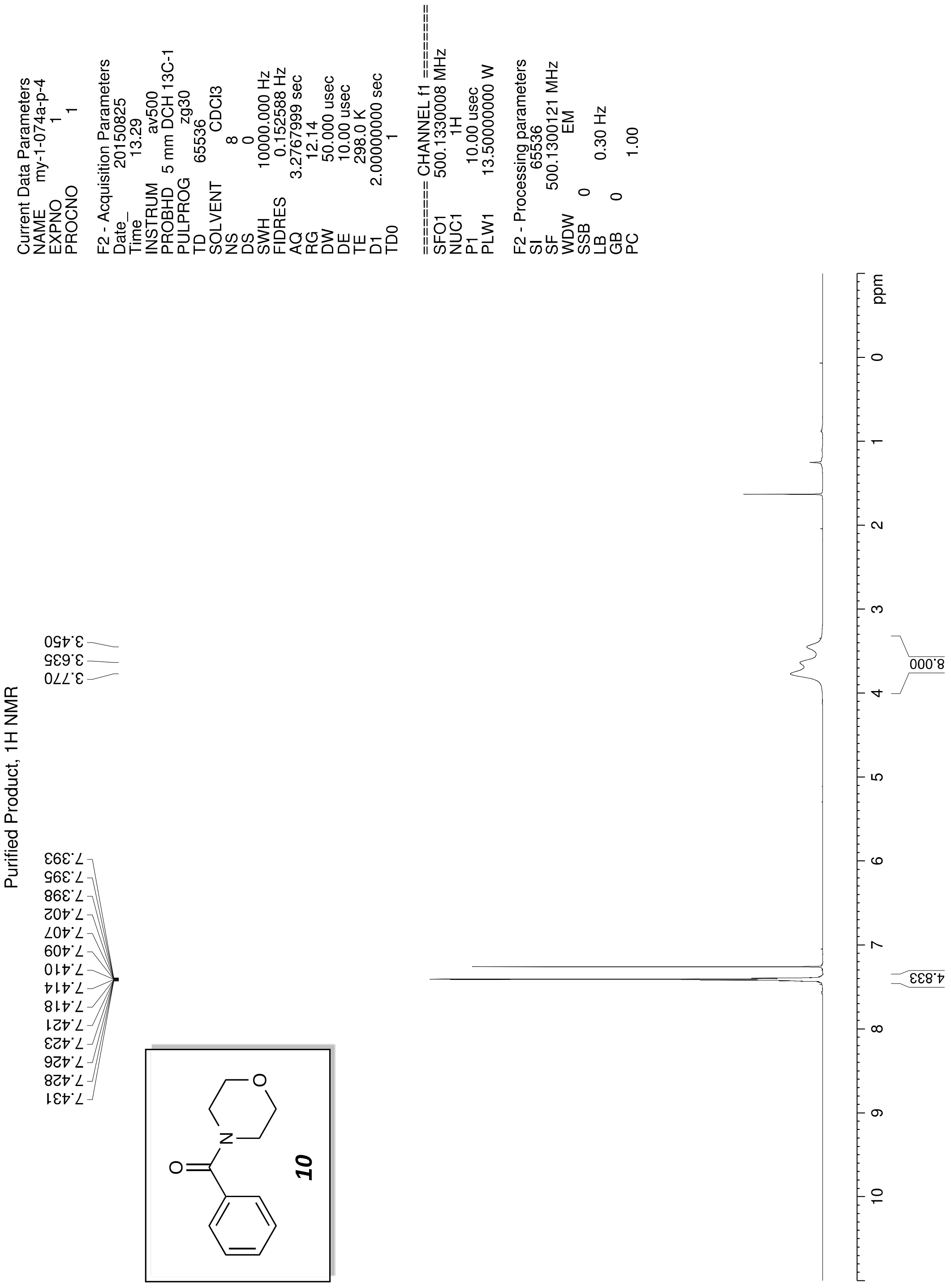


**Supplementary Figure 5. $^{1}$H NMR (500 MHz, $CDCl_3$) of 10**

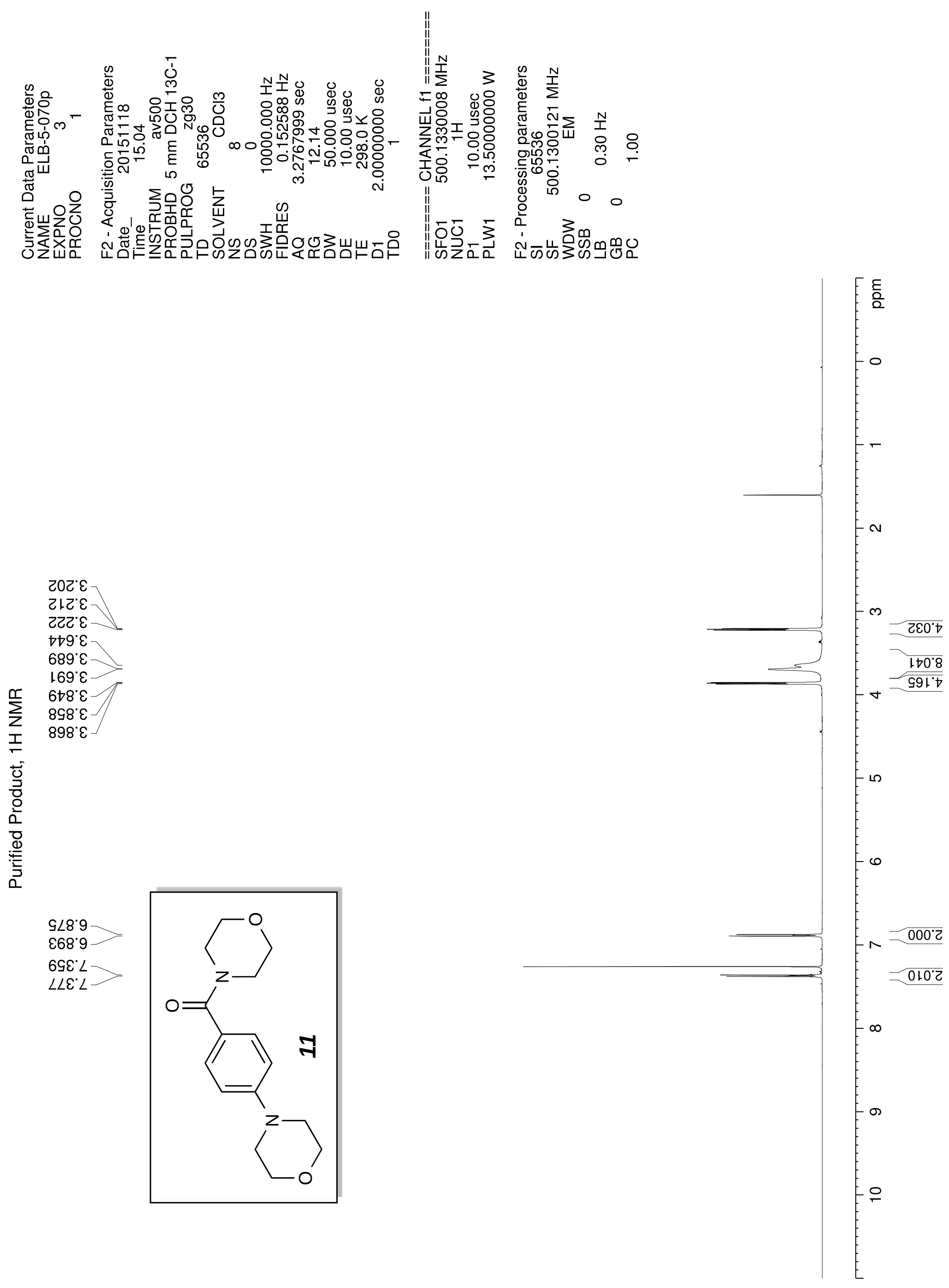


**Supplementary Figure 6. $^{1}$H NMR (500 MHz, $CDCl_3$) of 11**

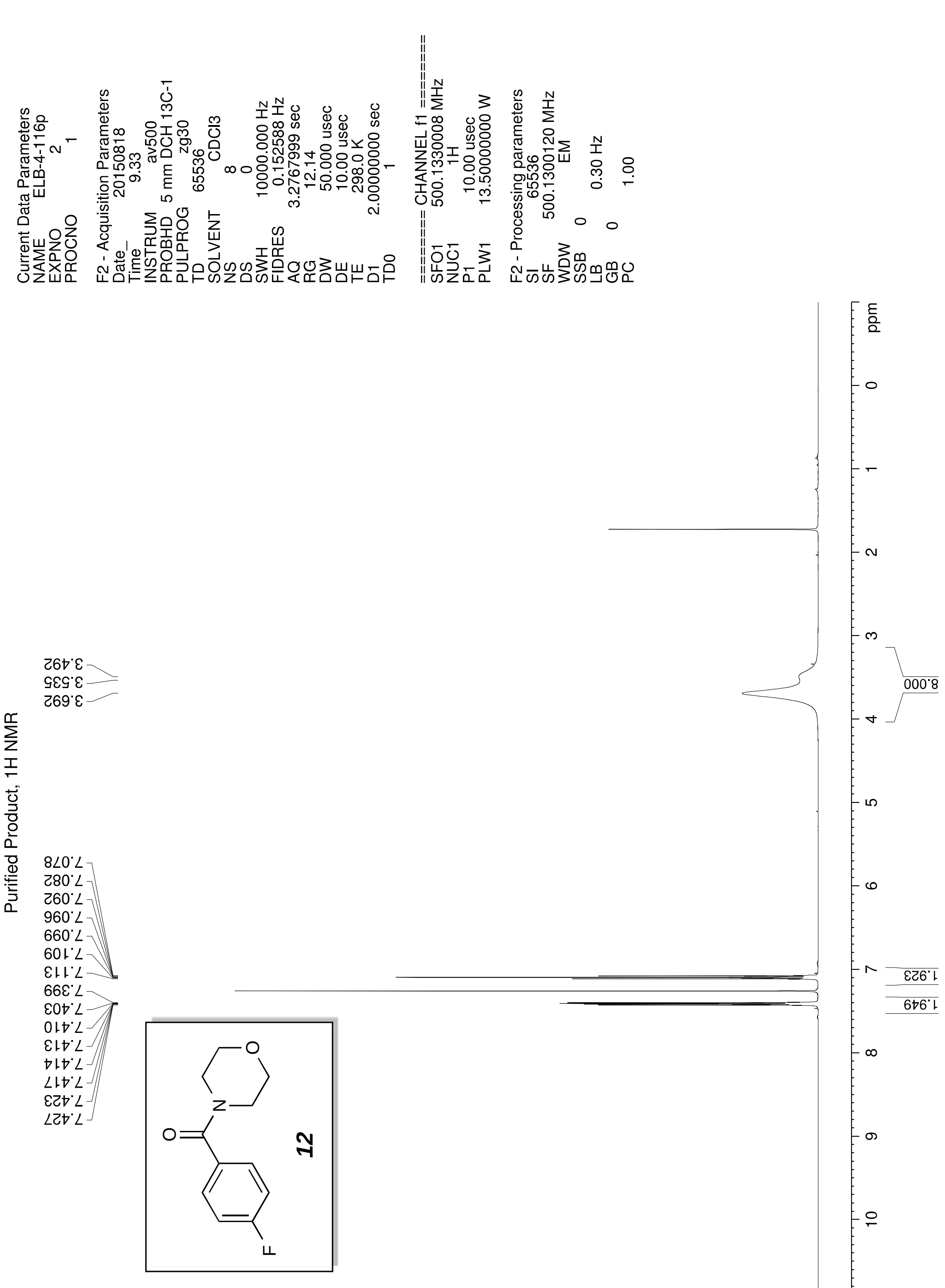


**Supplementary Figure 7. $^1$H NMR (500 MHz, $CDCl_3$) of 12**

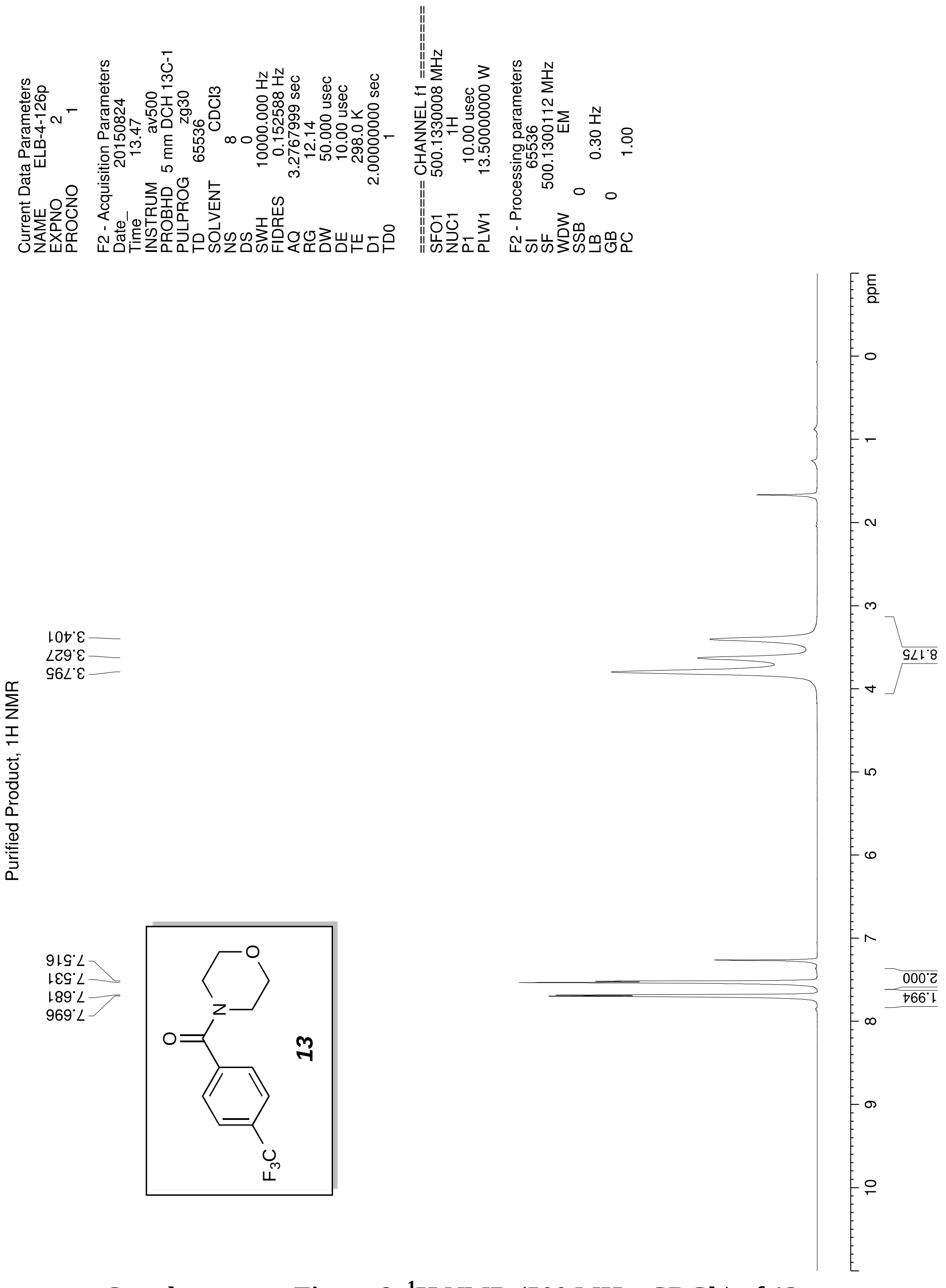


**Supplementary Figure 8. $^1$H NMR (500 MHz, $CDCl_3$) of 13**

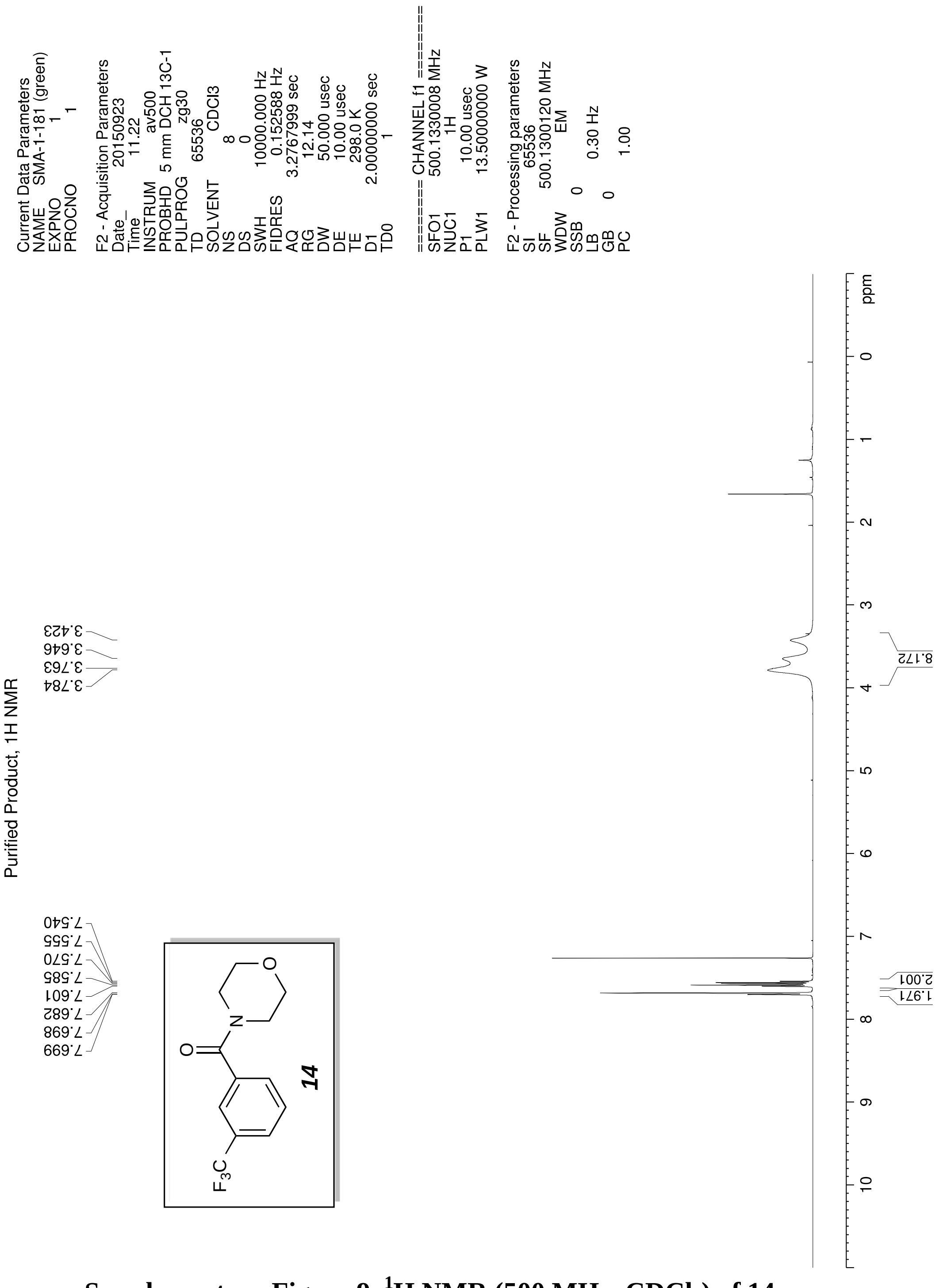


**Supplementary Figure 9. $^1$H NMR (500 MHz, $CDCl_3$) of 14**

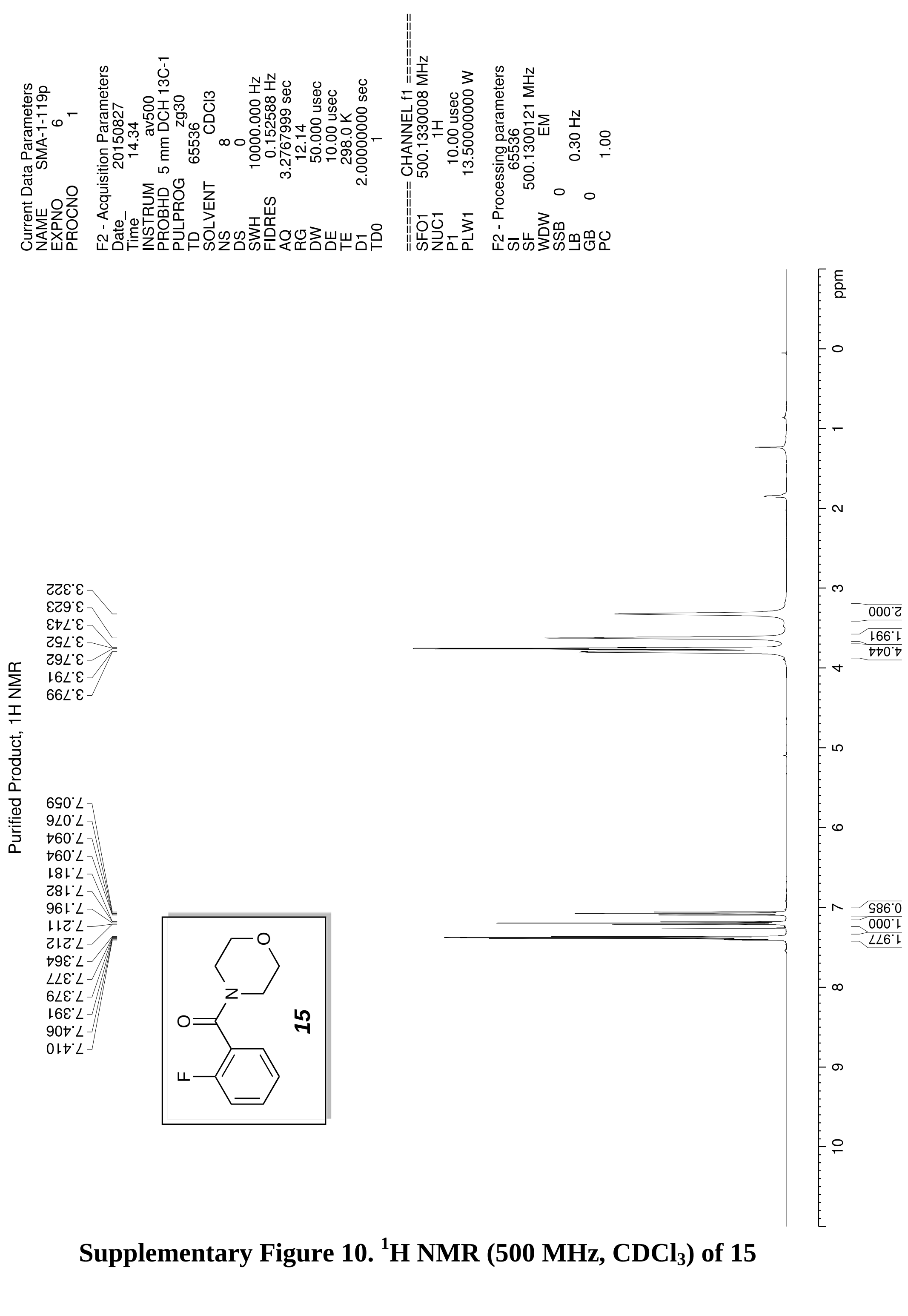


**Supplementary Figure 10.** **$^{1}$H NMR (500 MHz, $CDCl_3$) of 15**

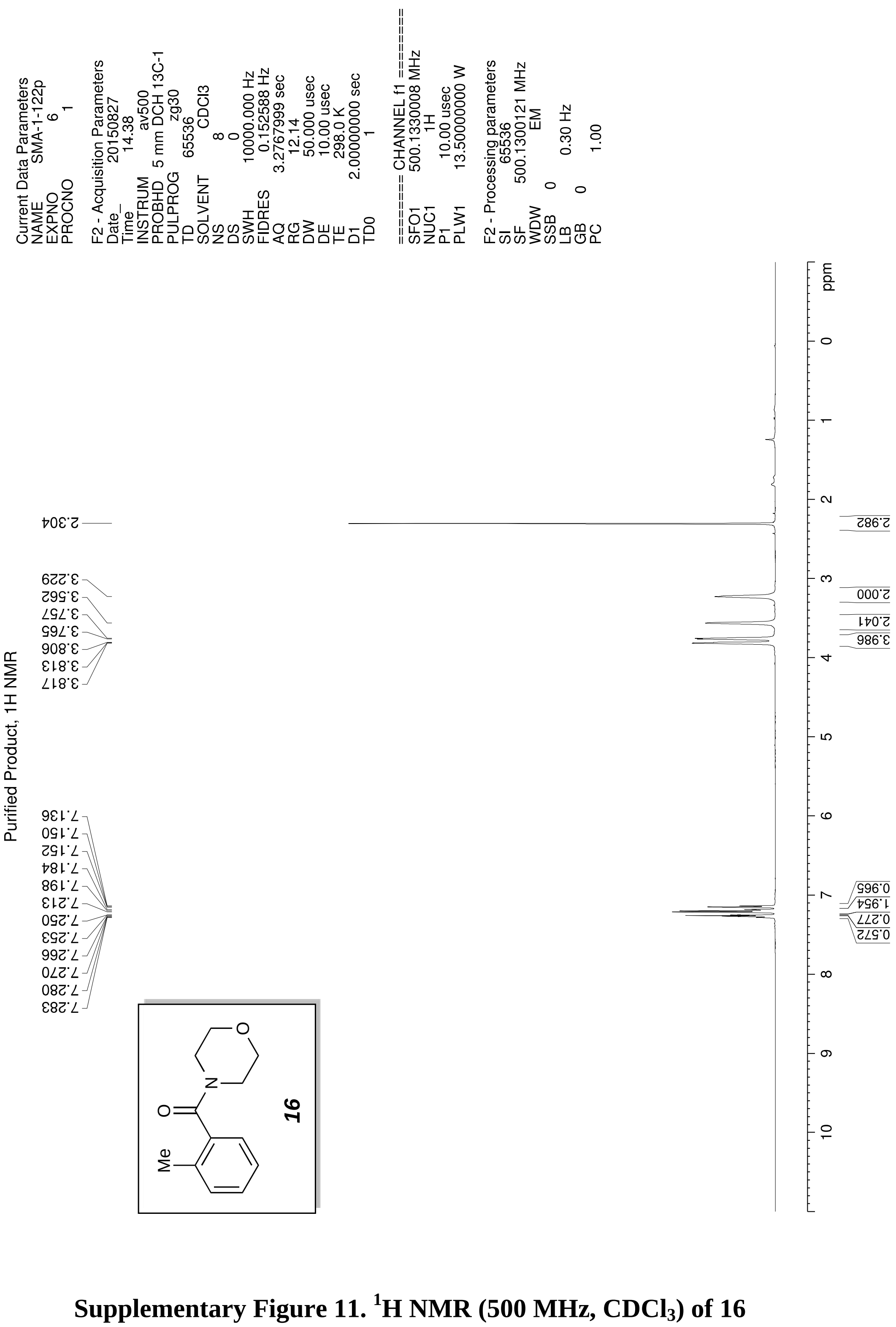


**Supplementary Figure 11. $^{1}$H NMR (500 MHz, $CDCl_3$) of 16**

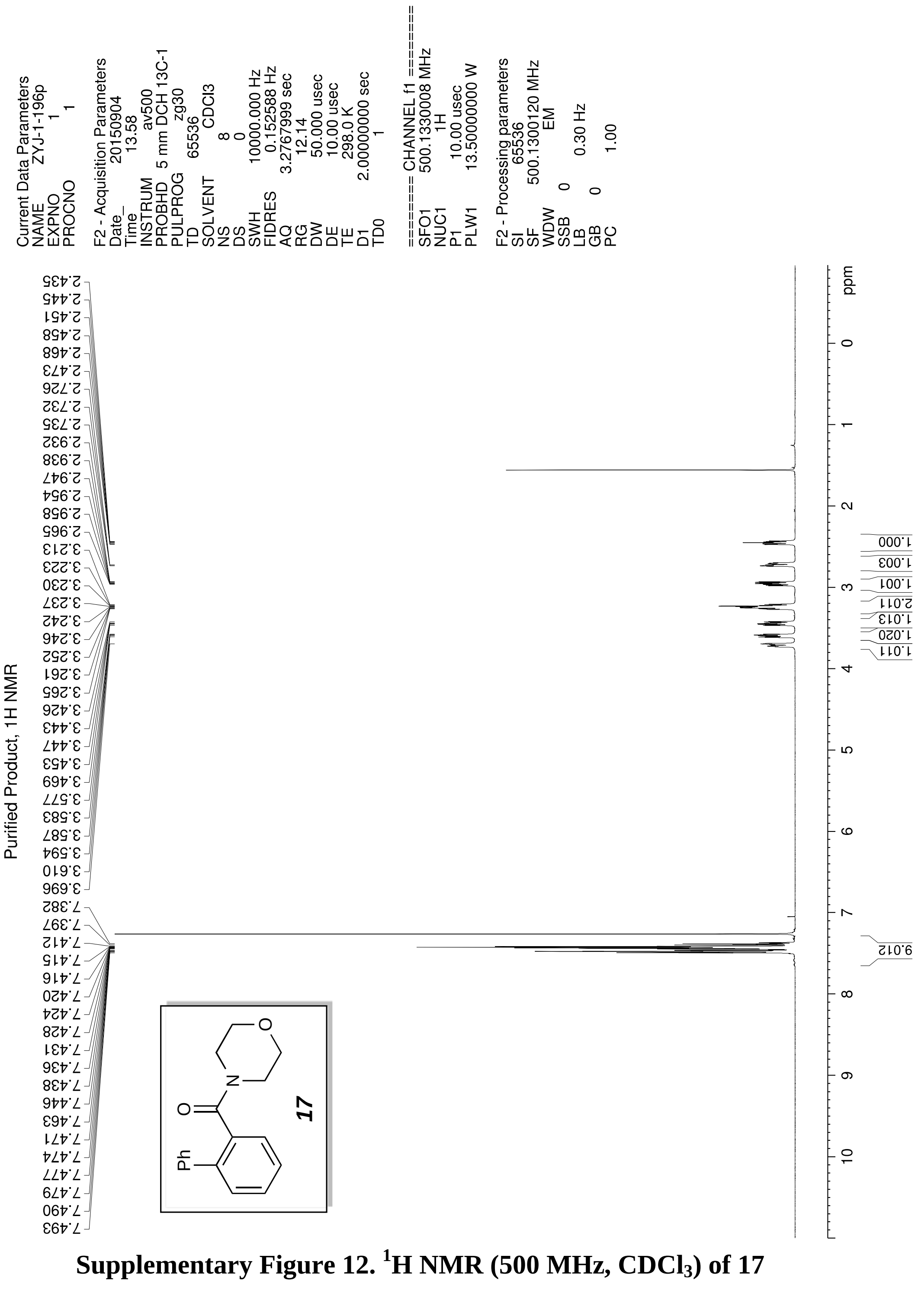


**Supplementary Figure 12. $^{1}$H NMR (500 MHz, $CDCl_3$) of 17**

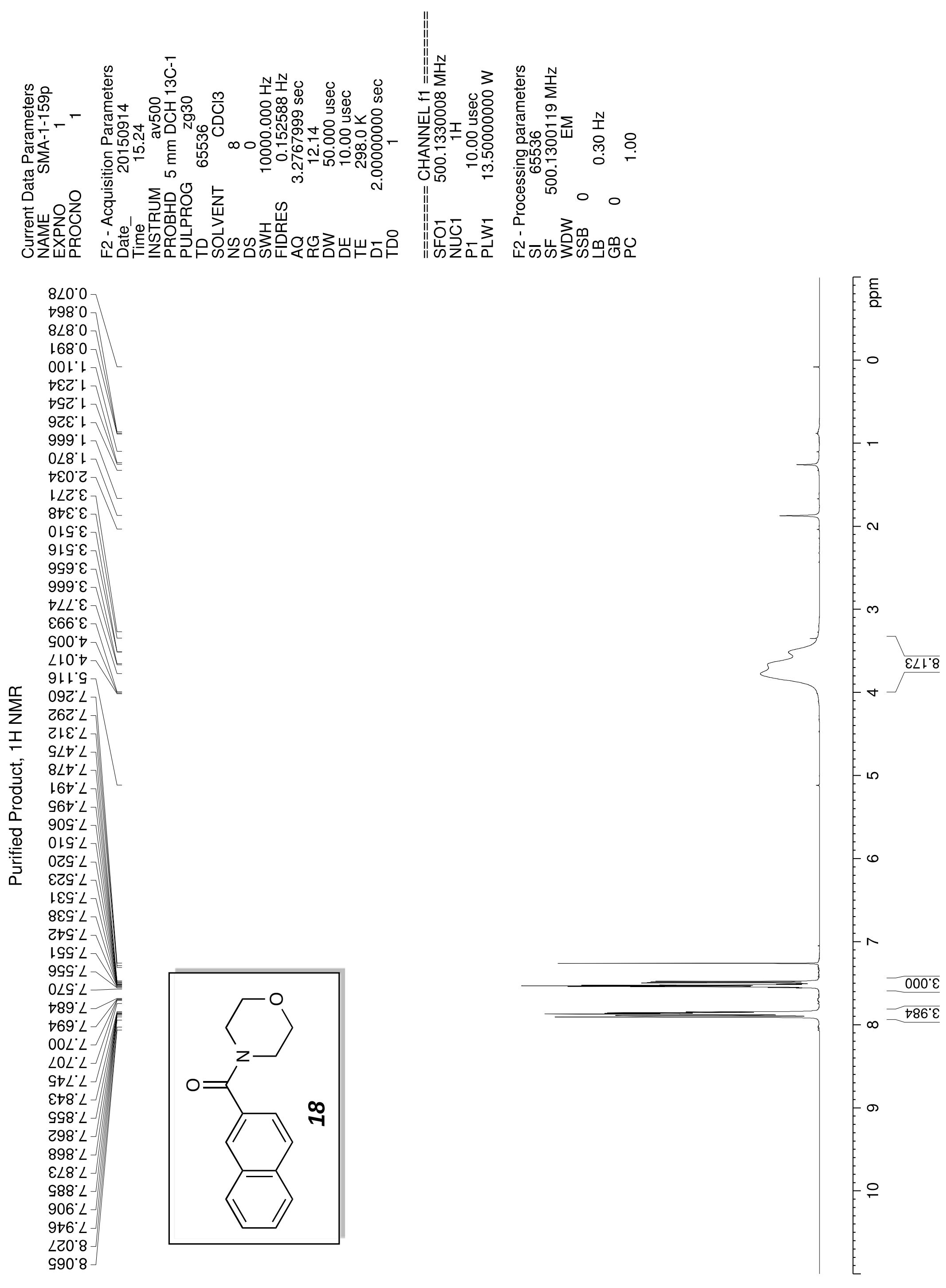


**Supplementary Figure 13. $^1$H NMR (500 MHz, $CDCl_3$) of 18**

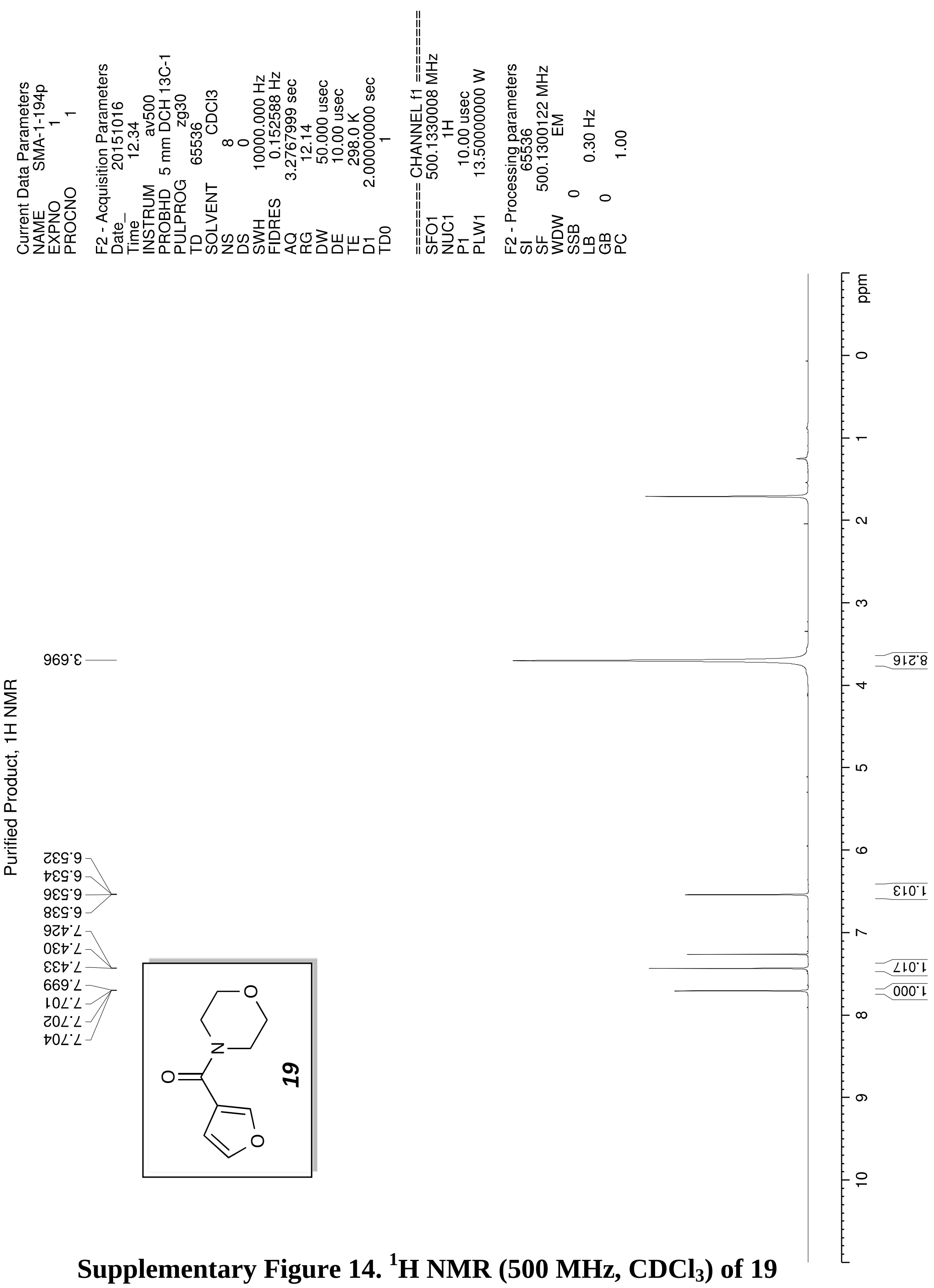


**Supplementary Figure 14. $^1$H NMR (500 MHz, $CDCl_3$) of 19**

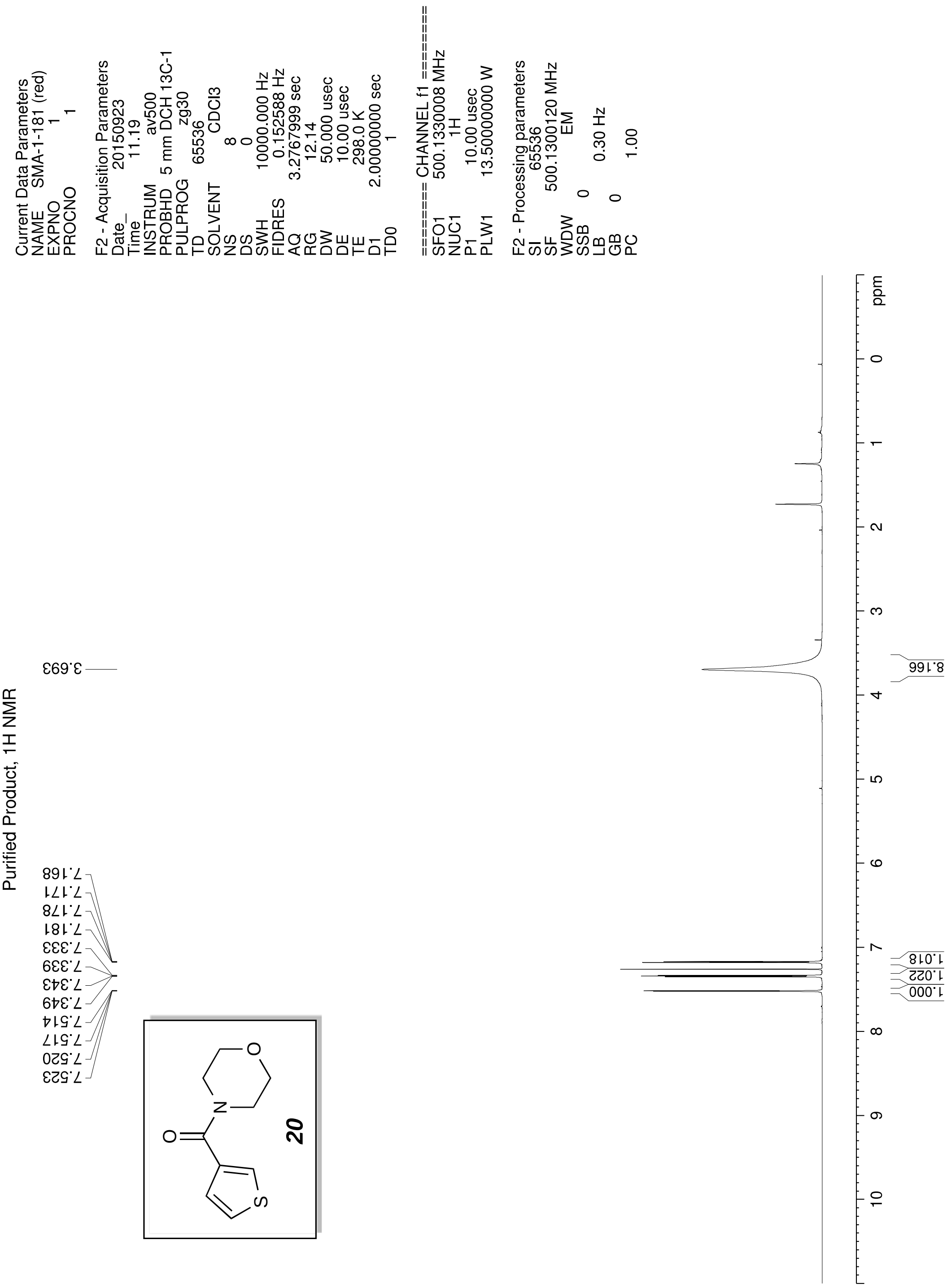


**Supplementary Figure 15. $^1$H NMR (500 MHz, $CDCl_3$) of 20**

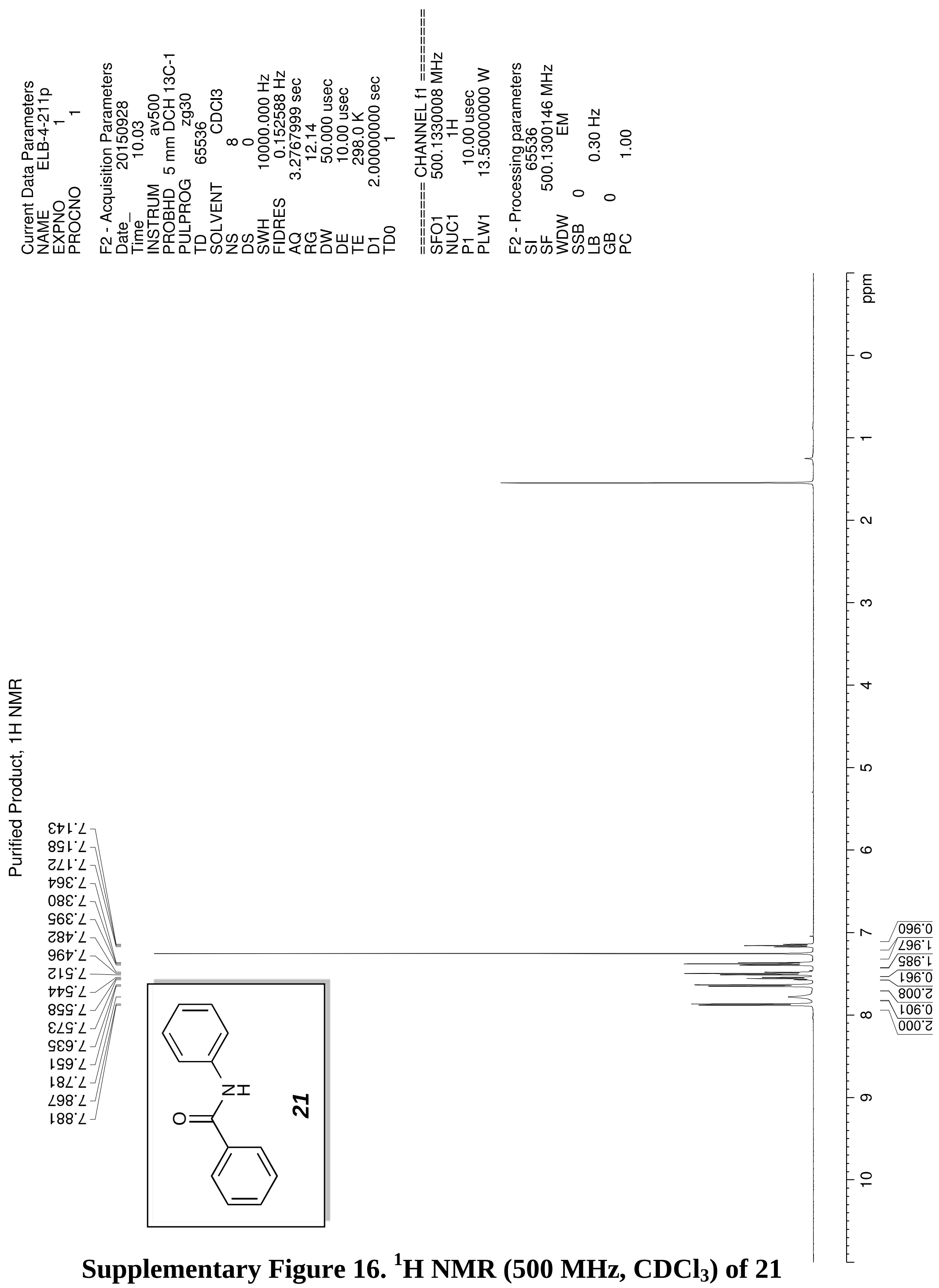


**Supplementary Figure 16. $^{1}$H NMR (500 MHz, $CDCl_3$) of 21**

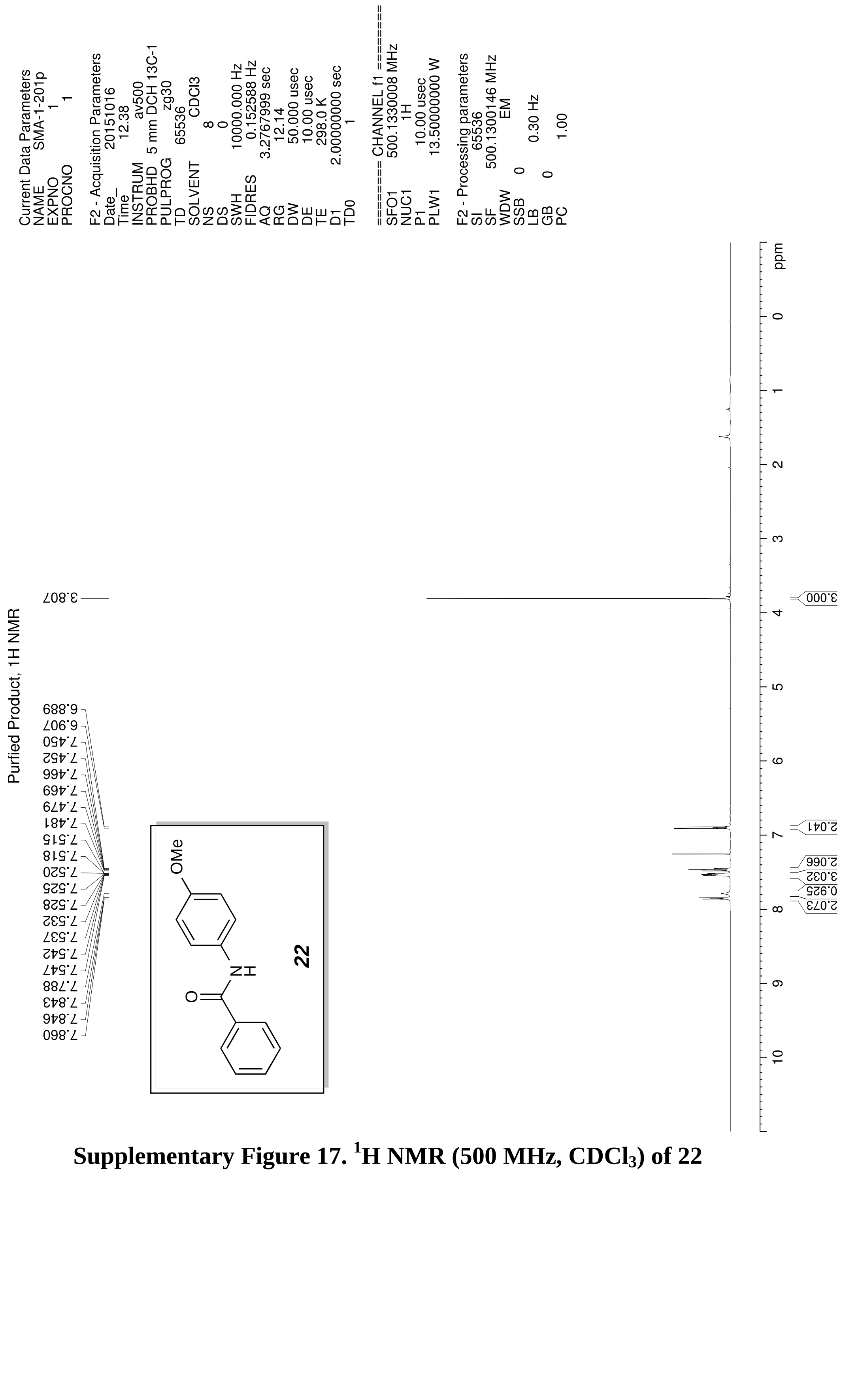


**Supplementary Figure 17. $^1$H NMR (500 MHz, $CDCl_3$) of 22**

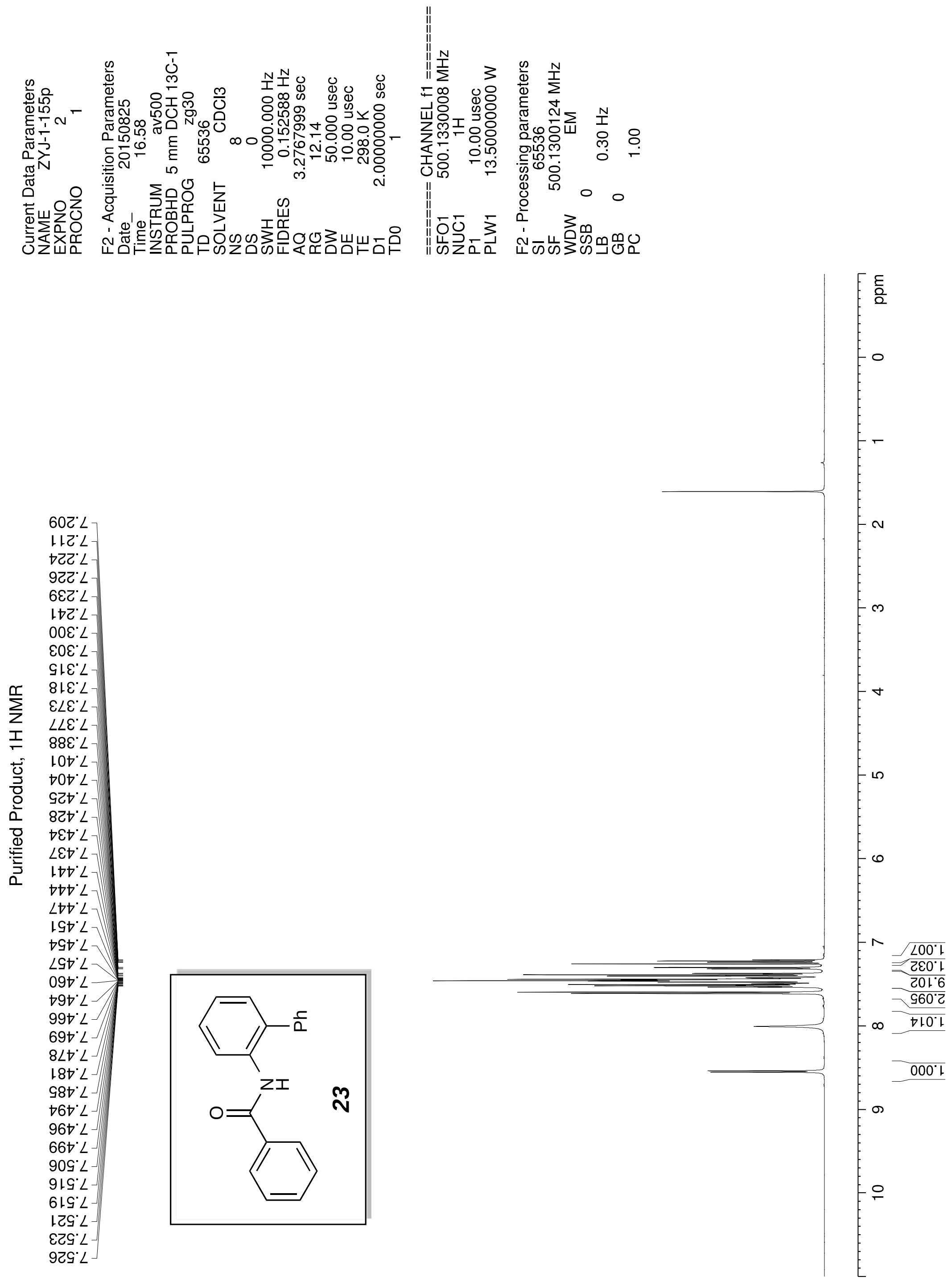

**Supplementary Figure 18. $^{1}$H NMR (500 MHz, $CDCl_3$) of 23**

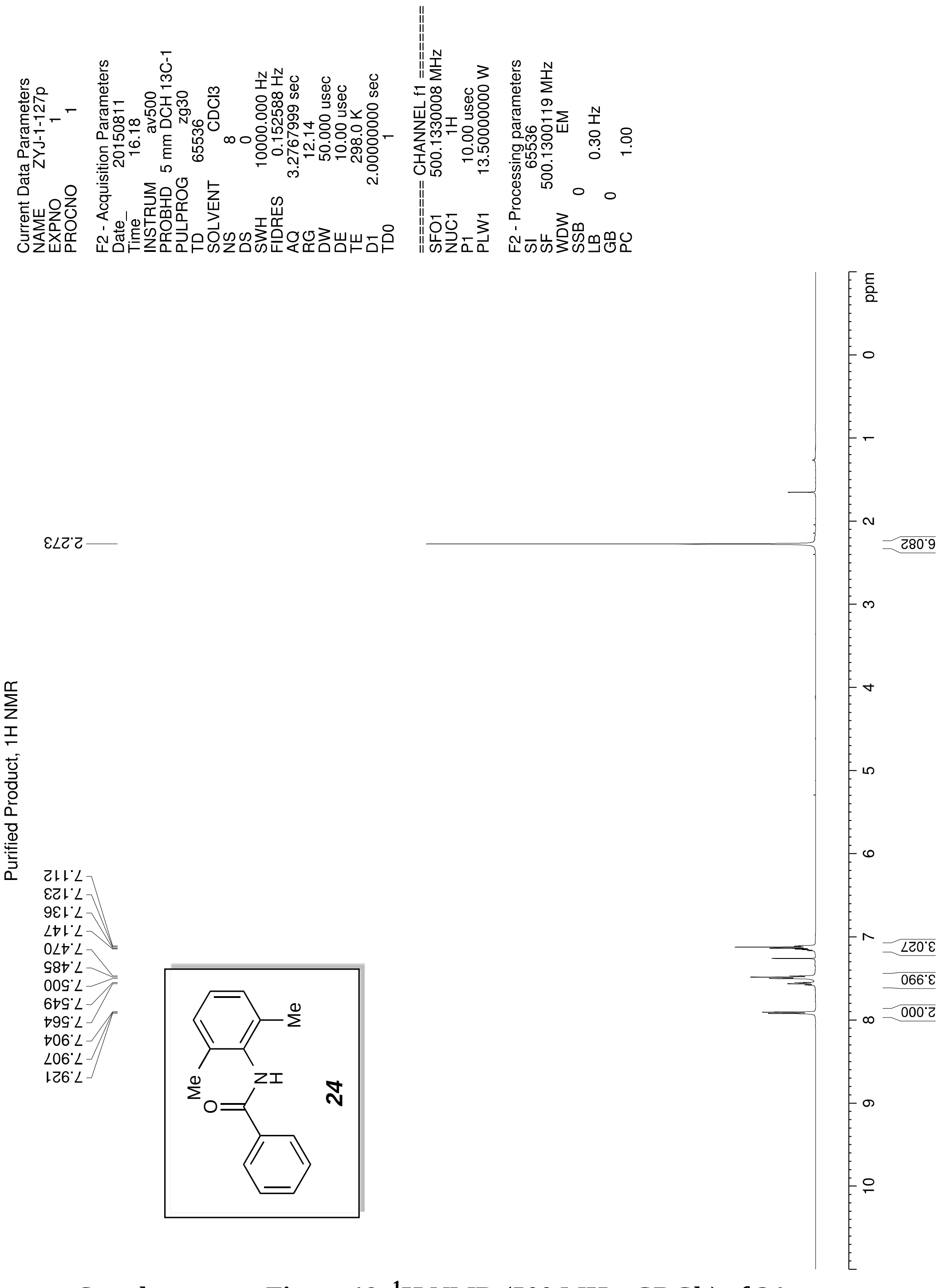


**Supplementary Figure 19. $^{1}$H NMR (500 MHz, $CDCl_3$) of 24**

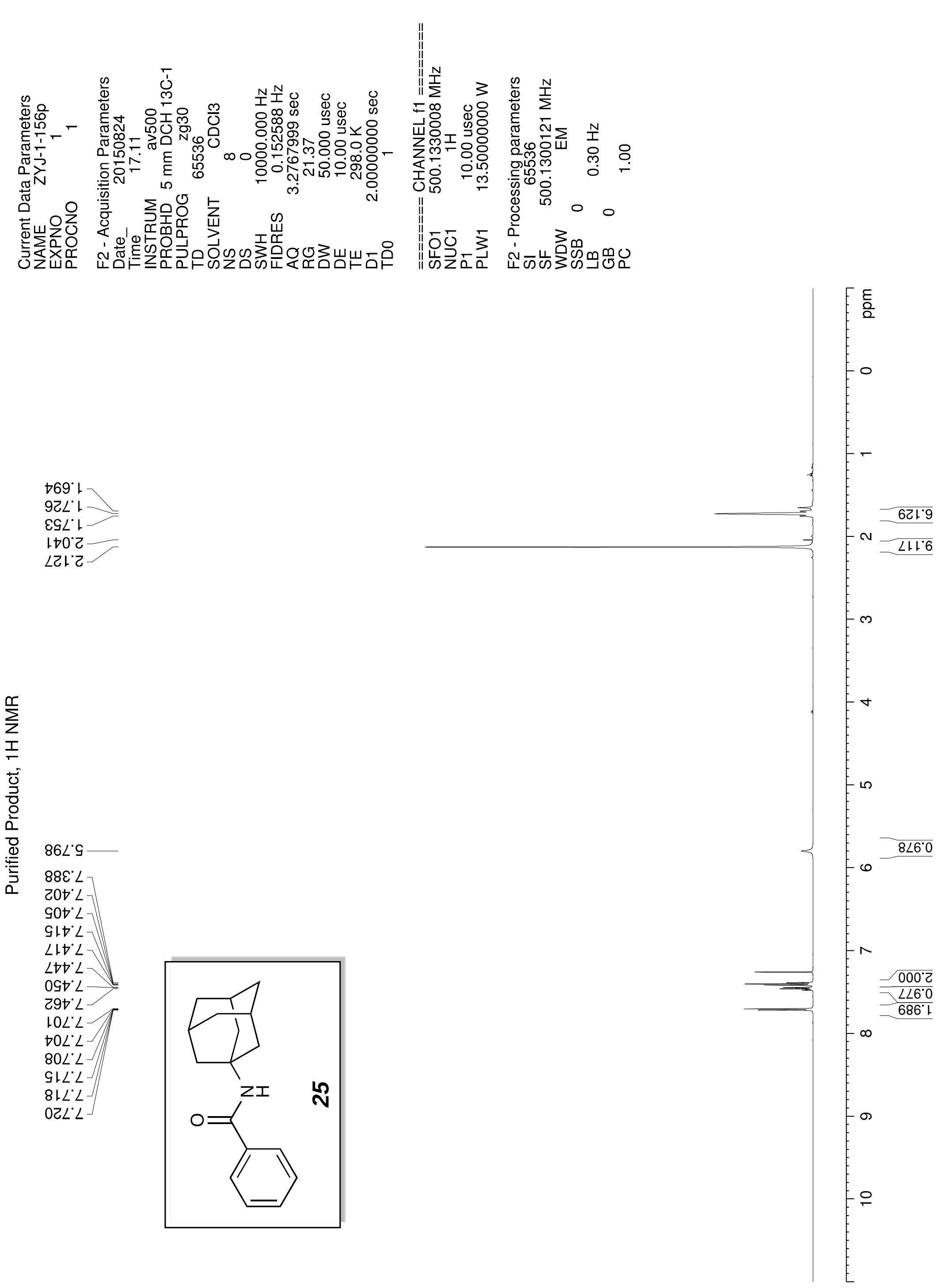


**Supplementary Figure 20. $^{1}$H NMR (500 MHz, $CDCl_3$) of 25**

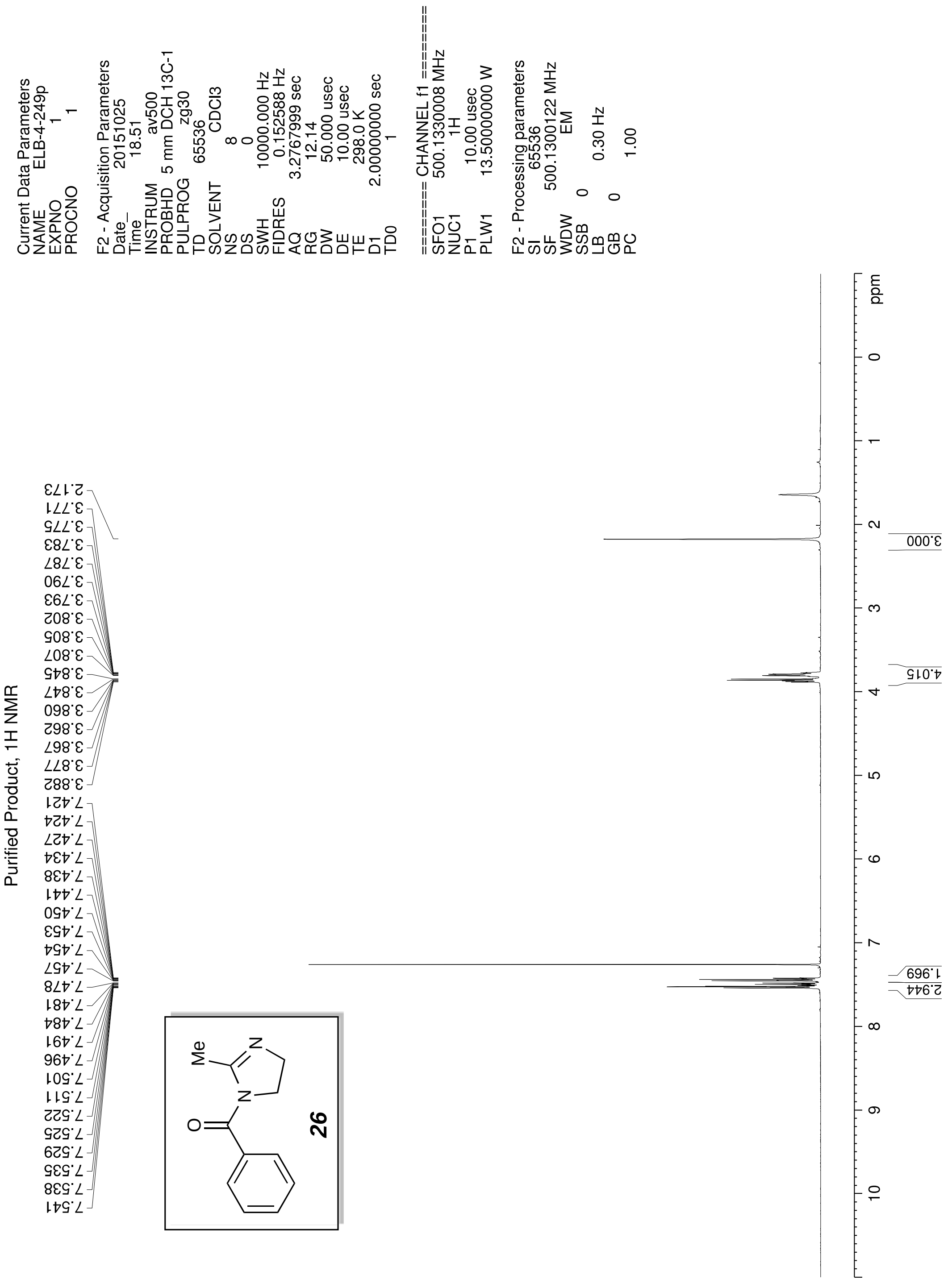


**Supplementary Figure 21. $^{1}H$ NMR (500 MHz, $CDCl_3$) of 26**

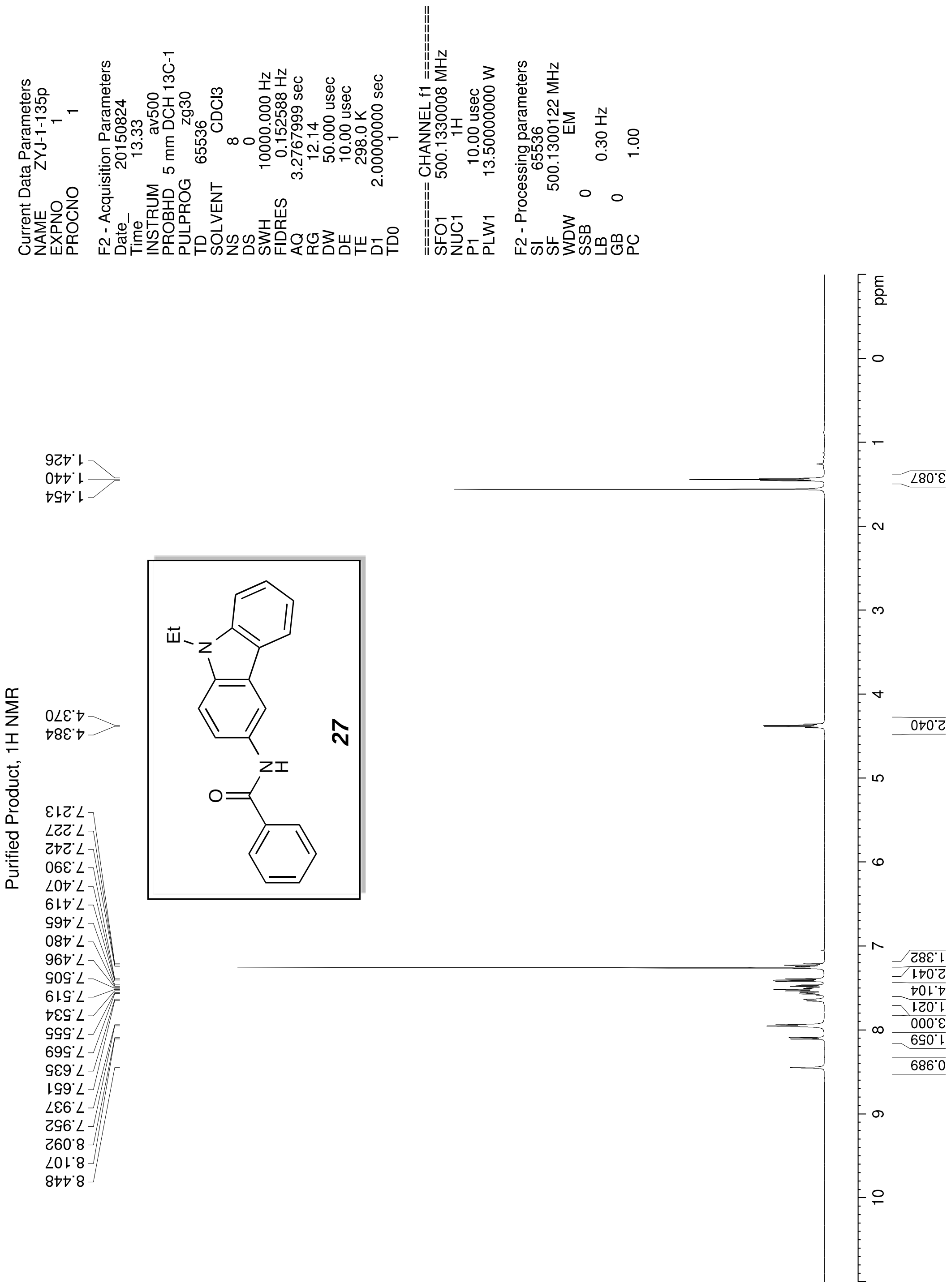


**Supplementary Figure 22. $^{1}$H NMR (500 MHz, $CDCl_3$) of 27**

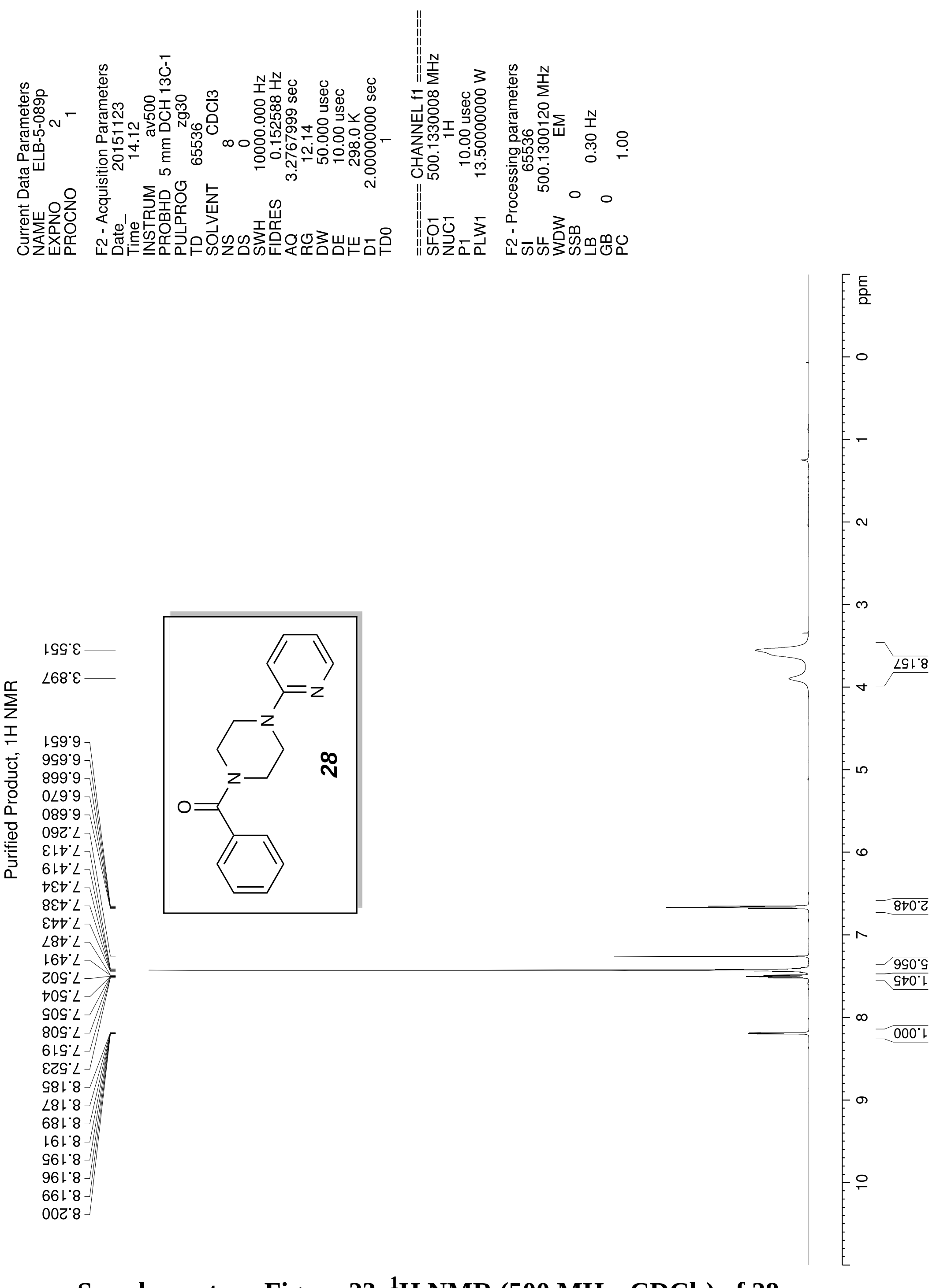

**Supplementary Figure 23. $^{1}H$ NMR (500 MHz, $CDCl_3$) of 28**

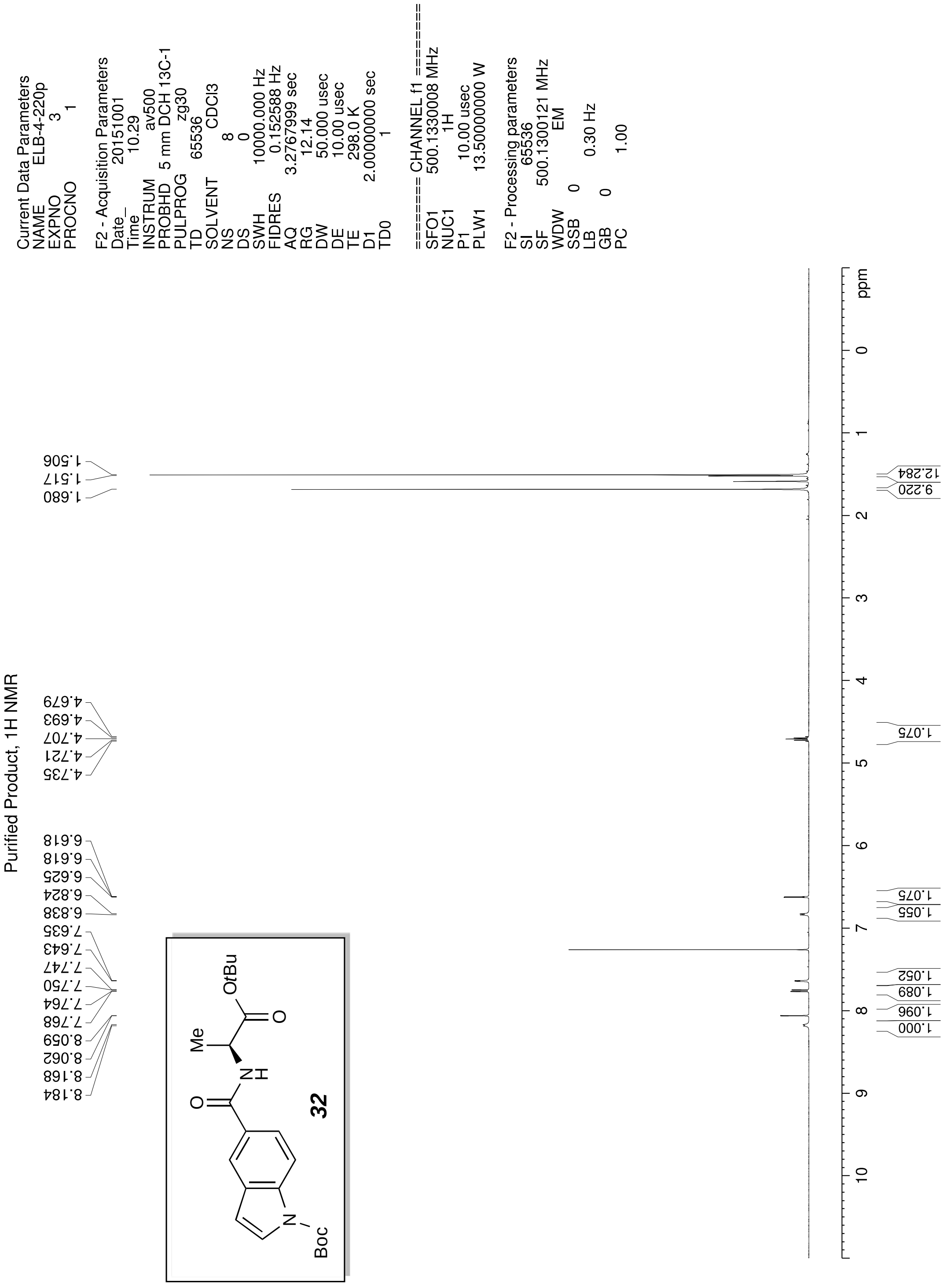


**Supplementary Figure 24.** $^{1}$**H NMR (500 MHz, CDCl$_3$) of 32**

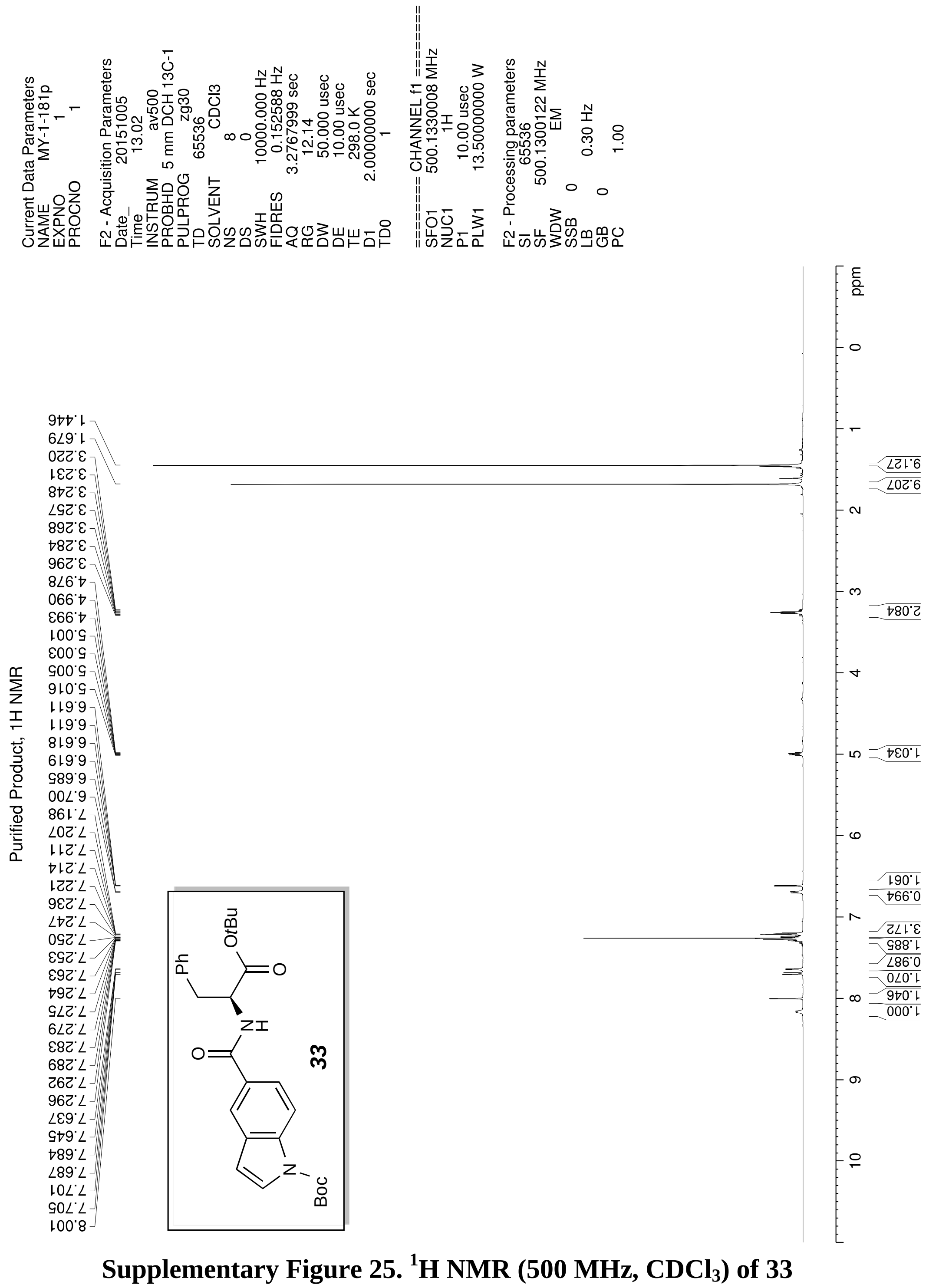


**Supplementary Figure 25. $^1$H NMR (500 MHz, $CDCl_3$) of 33**

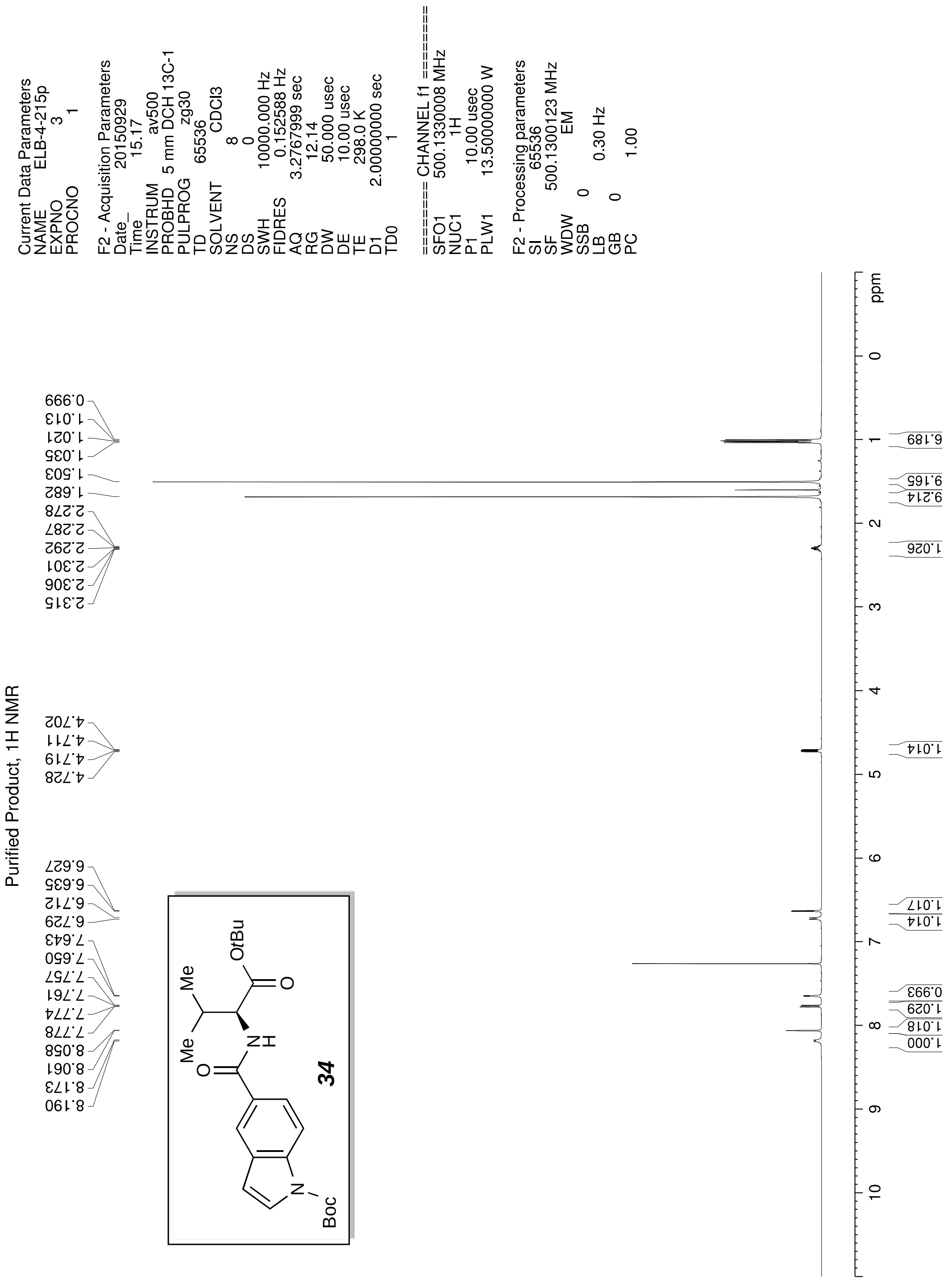


**Supplementary Figure 26. $^{1}$H NMR (500 MHz, $CDCl_3$) of 34**

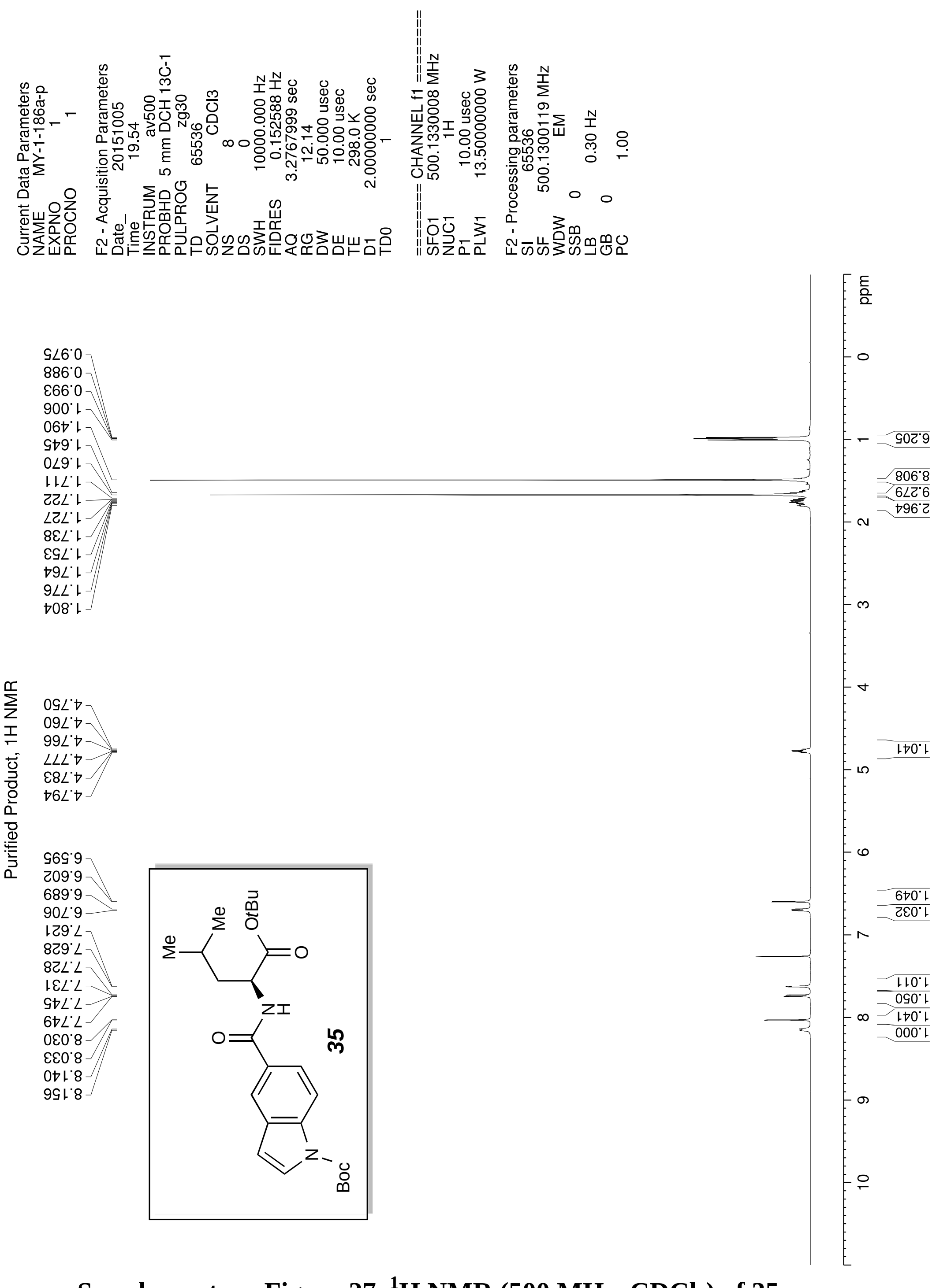


**Supplementary Figure 27. $^{1}$H NMR (500 MHz, $CDCl_3$) of 35**

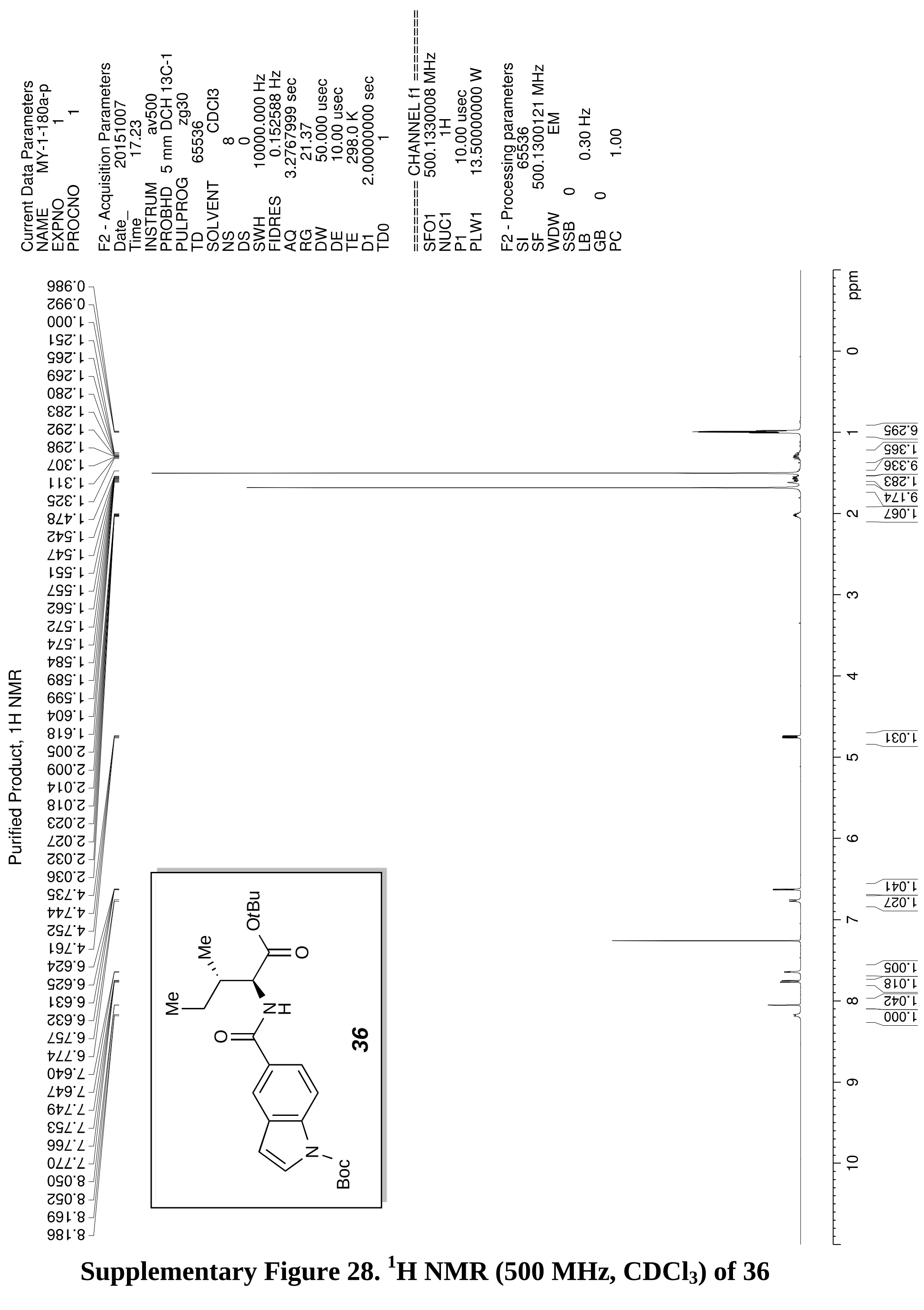


**Supplementary Figure 28. $^{1}$H NMR (500 MHz, $CDCl_3$) of 36**

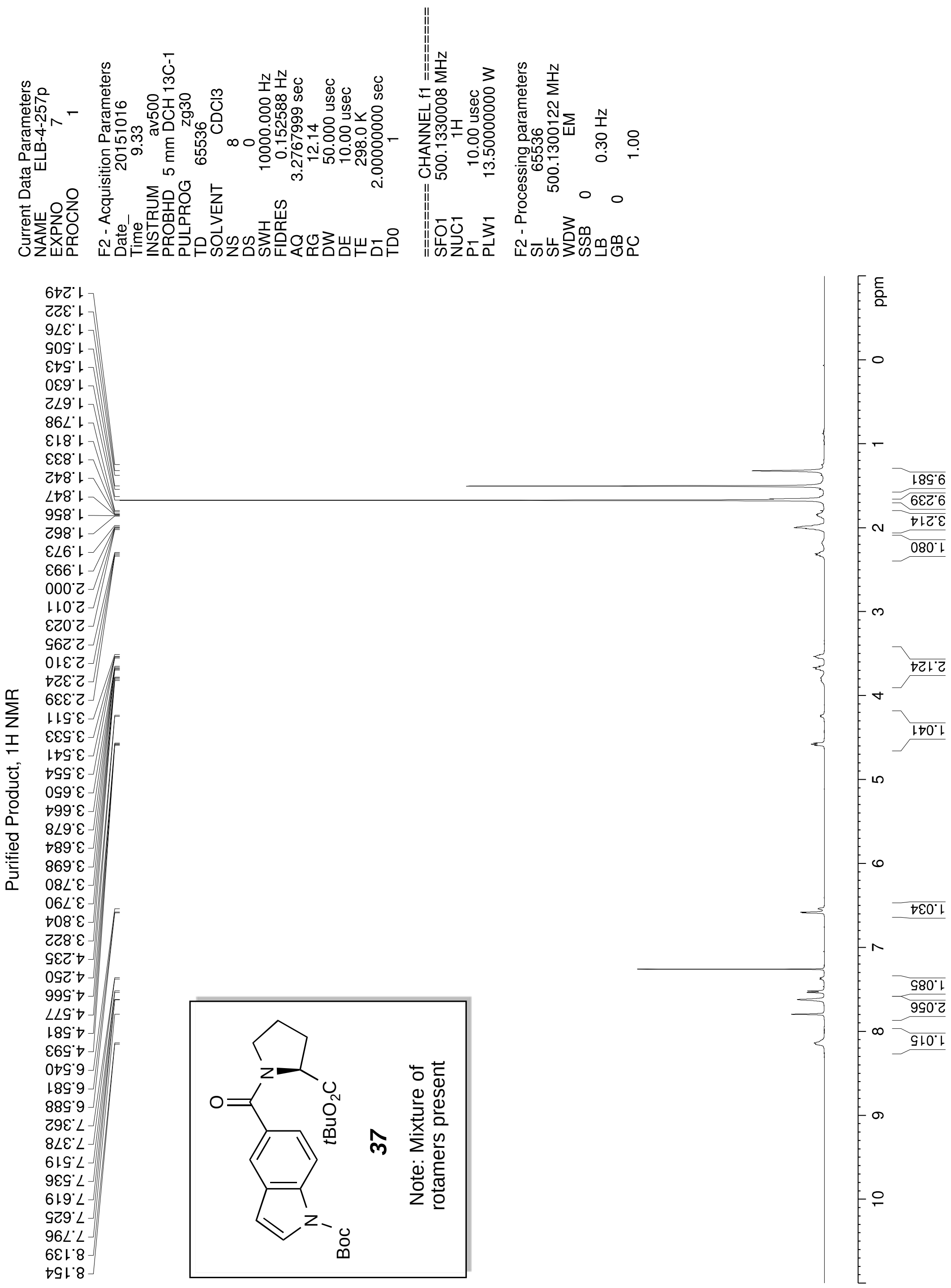


**Supplementary Figure 29. $^{1}H$ NMR (500 MHz, $CDCl_3$) of 37**

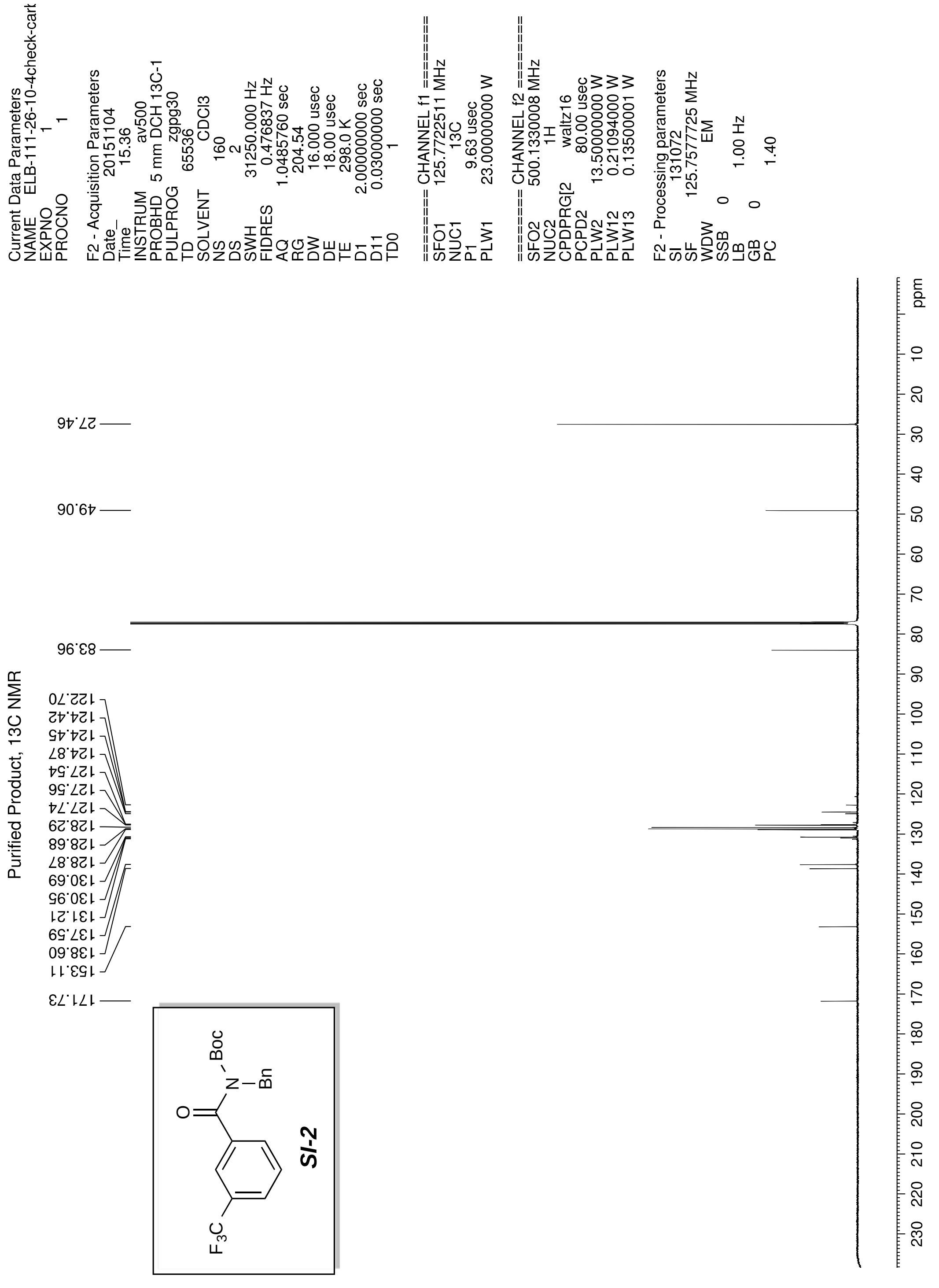


**Supplementary Figure 30.** $^{13}$**C NMR (500 MHz, CDCl$_3$) of SI-2**

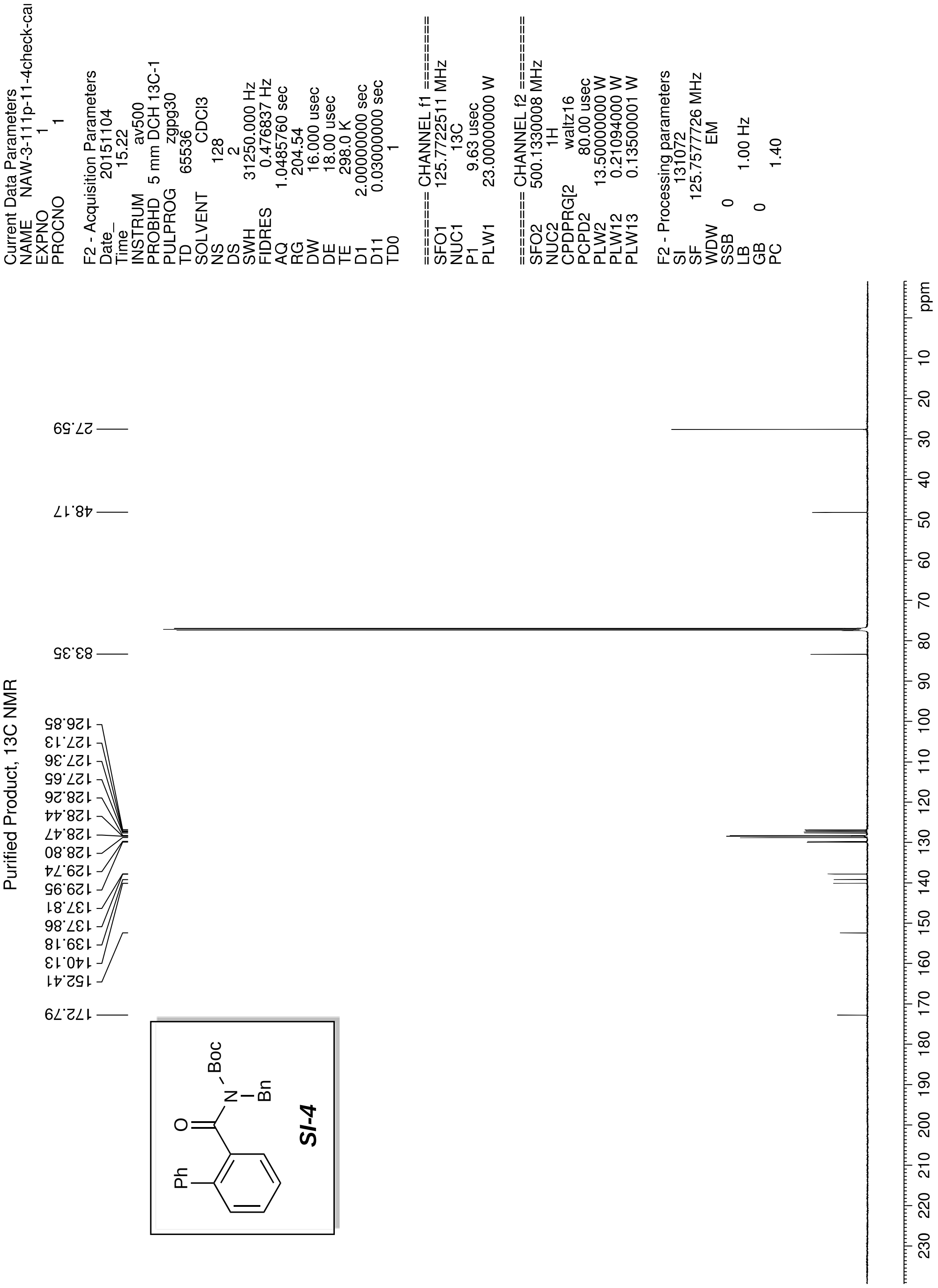


**Supplementary Figure 31.** $^{13}$**C NMR (500 MHz, CDCl$_3$) of SI-4**

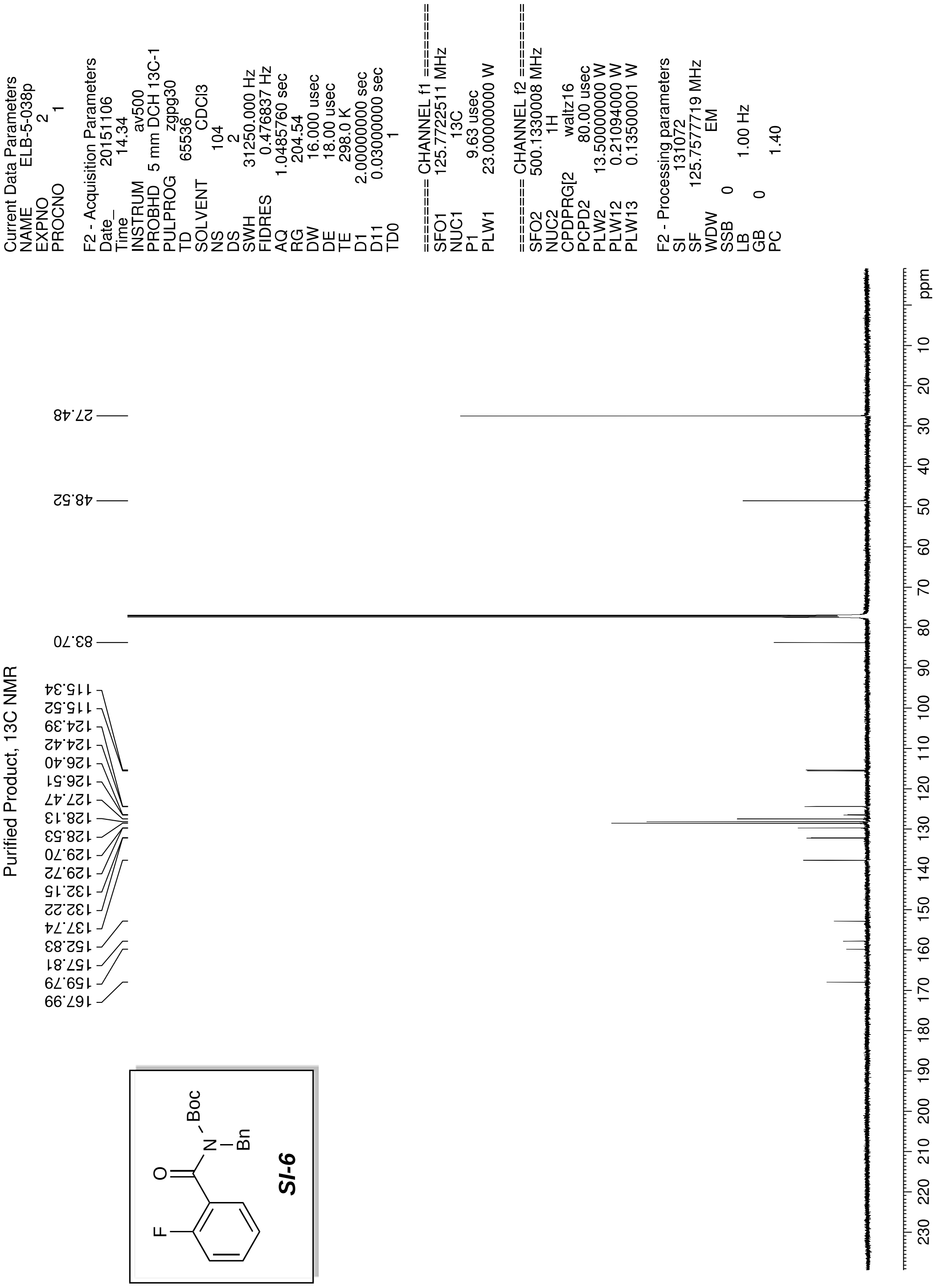


**Supplementary Figure 32.** $^{13}$**C NMR (500 MHz, CDCl$_3$) of SI-6**

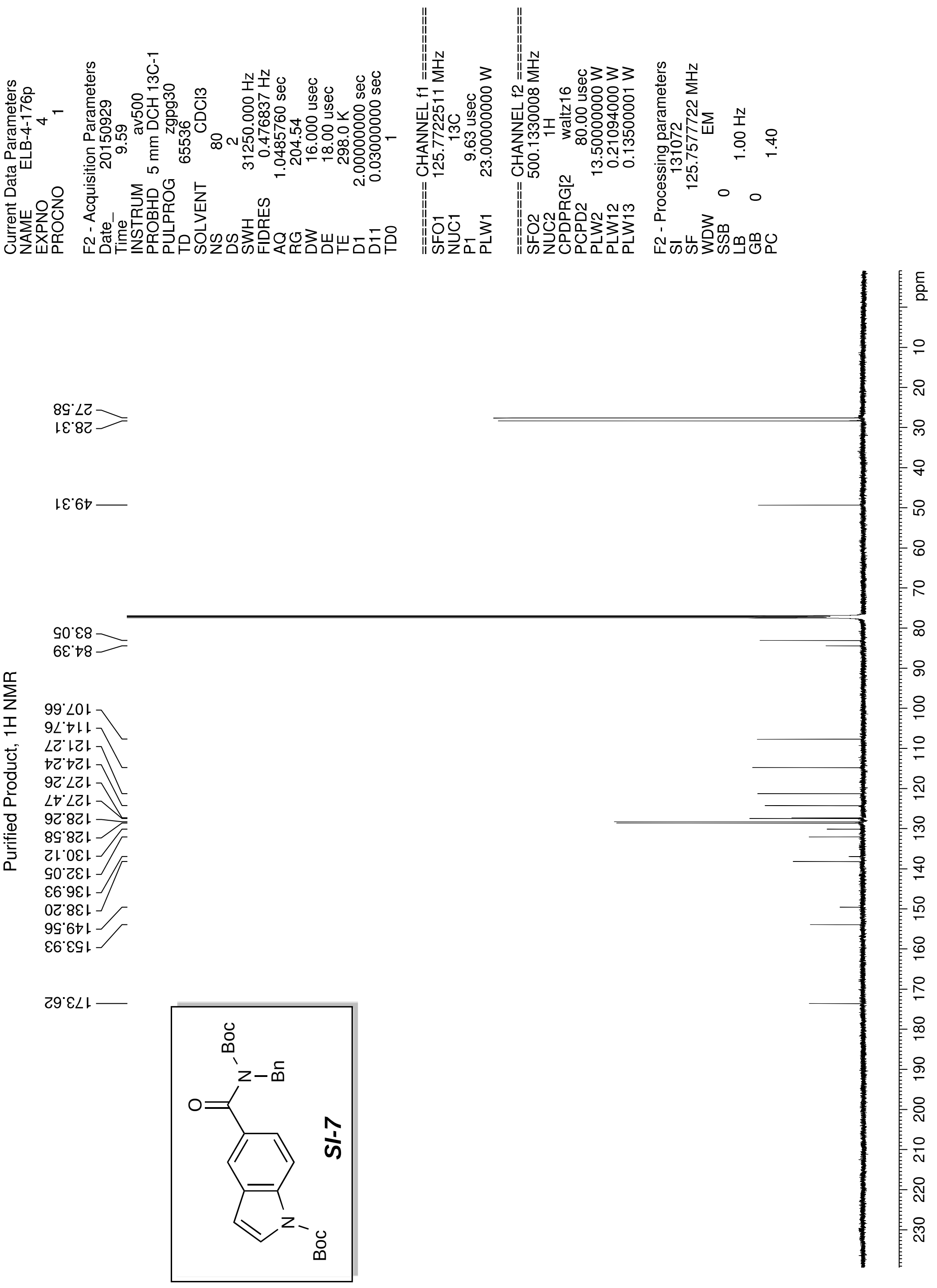


**Supplementary Figure 33.** $^{13}$C NMR (500 MHz, $CDCl_3$) of SI-7

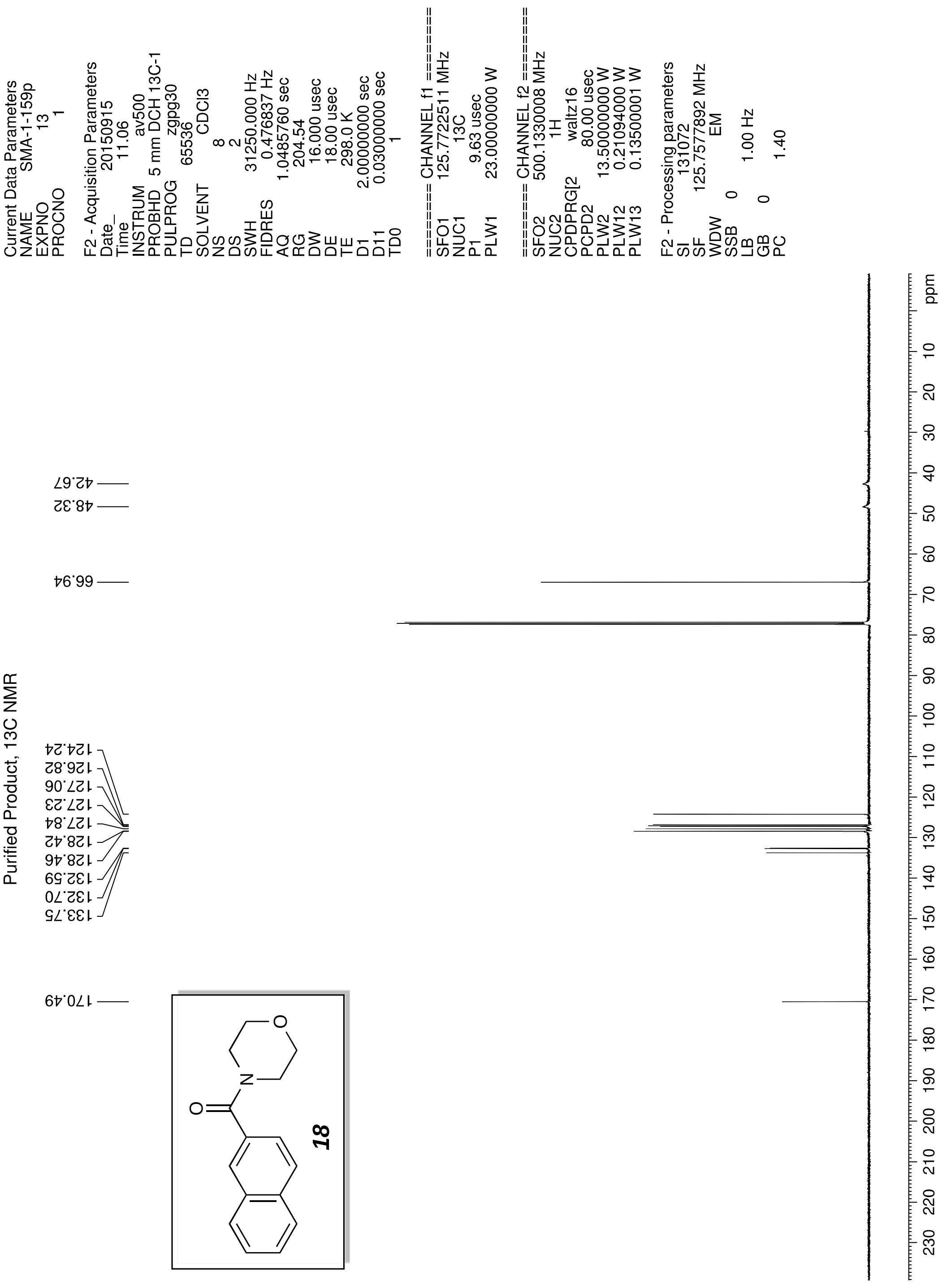


**Supplementary Figure 34. $^{13}$C NMR (500 MHz, $CDCl_3$) of 18**

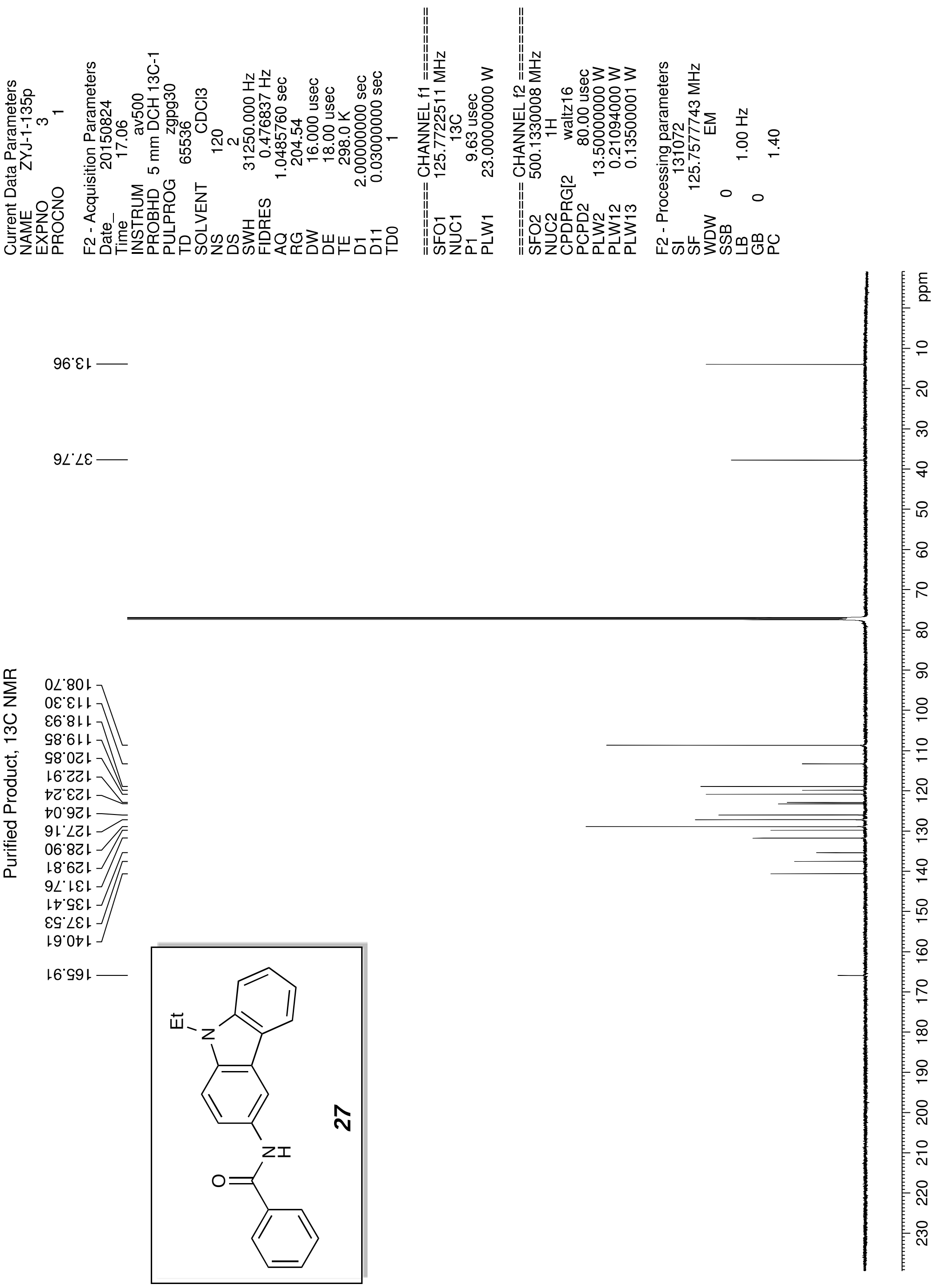


**Supplementary Figure 35.** $^{13}$**C NMR (500 MHz, CDCl**$_3$**) of 27**

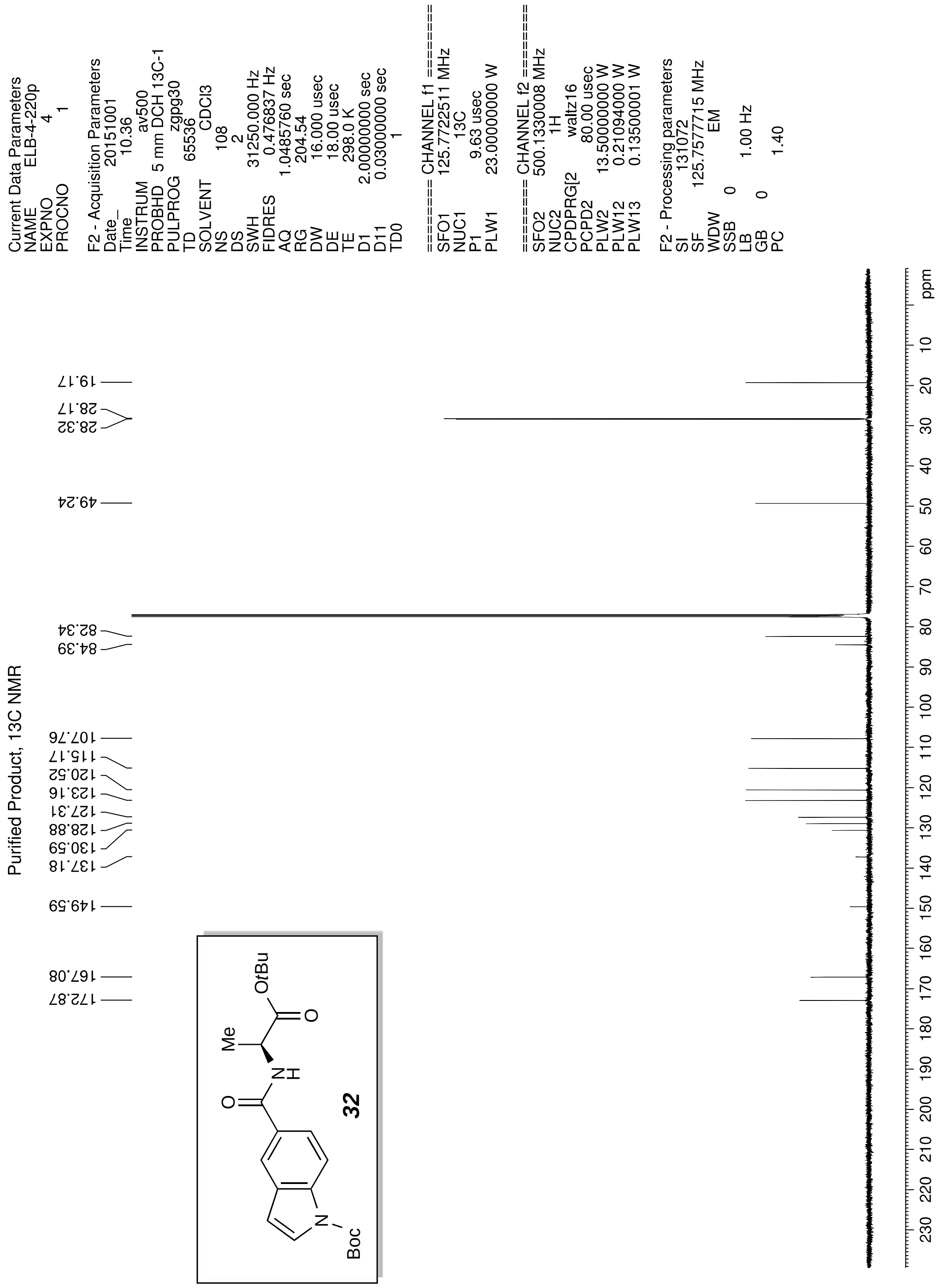


**Supplementary Figure 36. 13C NMR (500 MHz, $CDCl_3$) of 32**

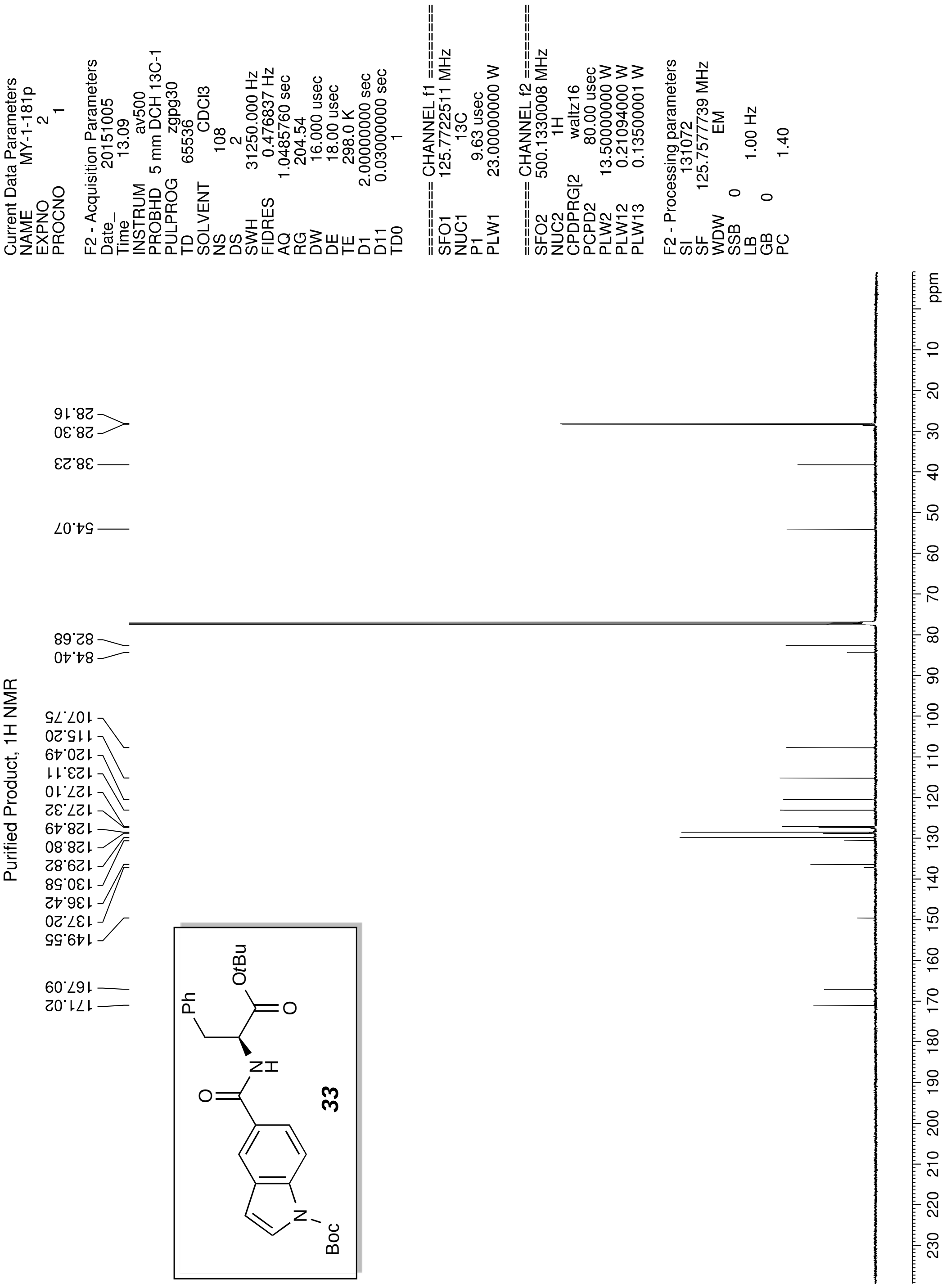


**Supplementary Figure 37.** $^{13}$**C NMR (500 MHz, CDCl$_3$) of 33**

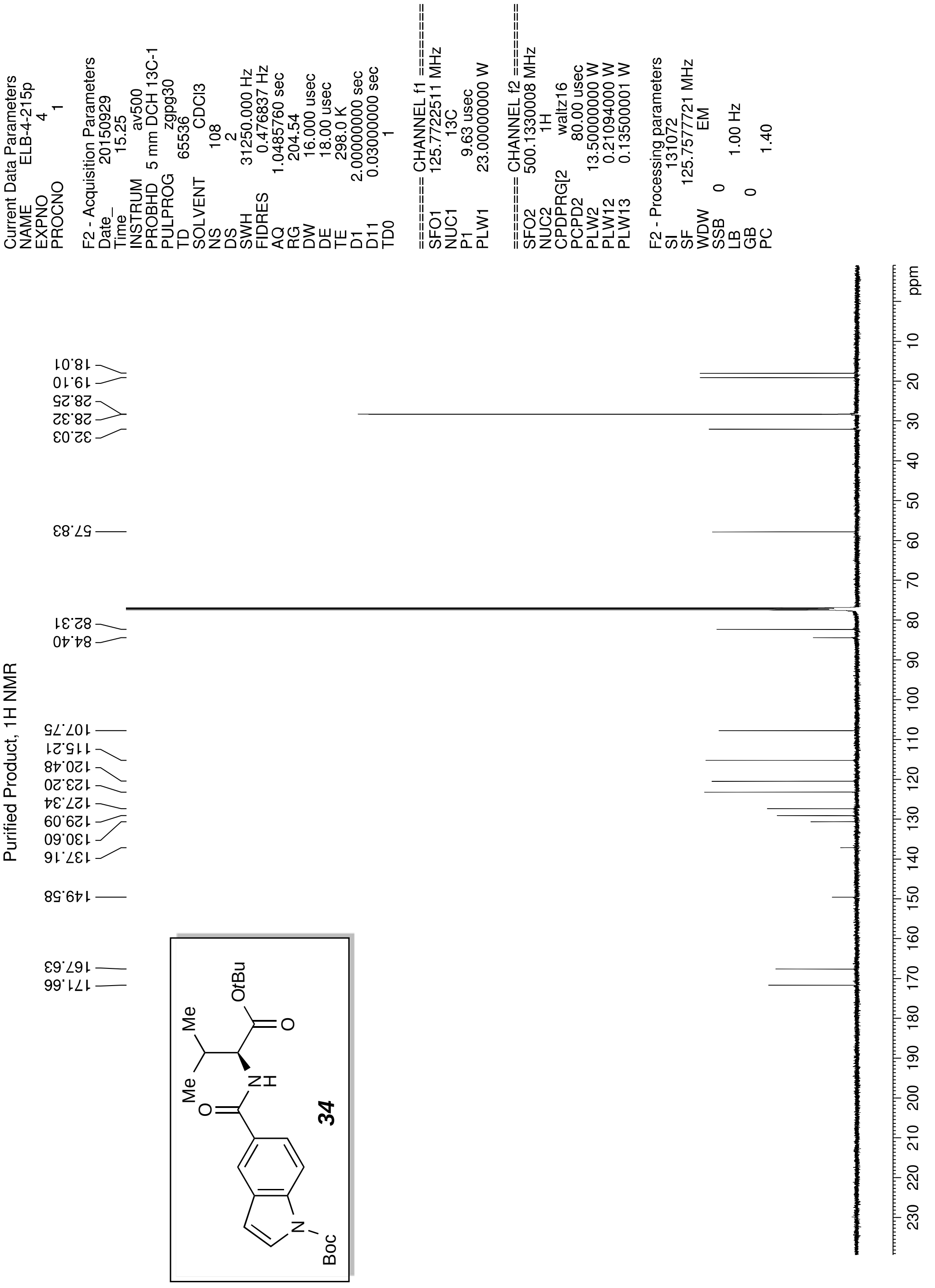


**Supplementary Figure 38.** $^{13}$**C NMR (500 MHz, CDCl$_3$) of 34**

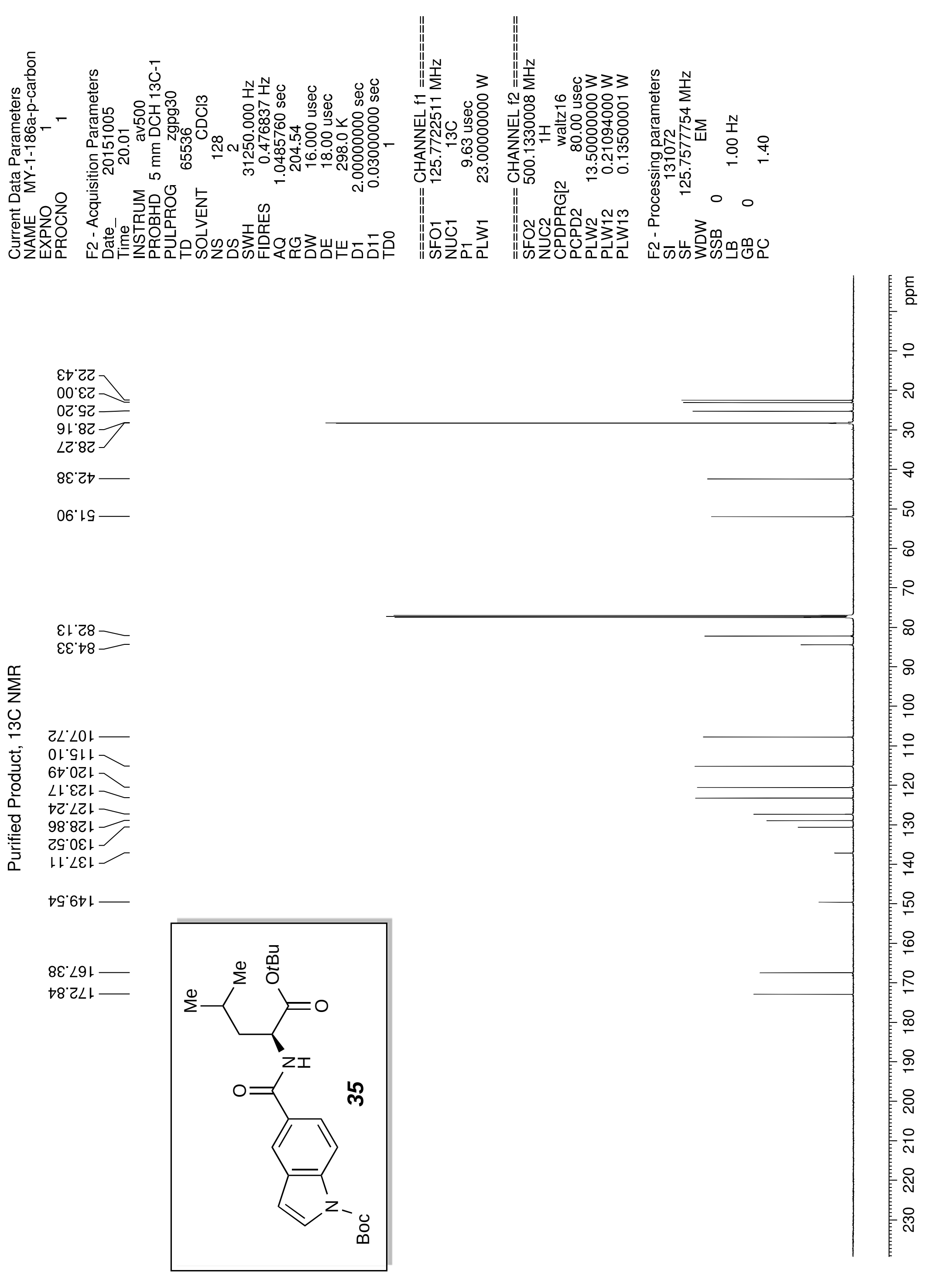


**Supplementary Figure 39.** $^{13}$**C NMR (500 MHz, CDCl$_3$) of 35**

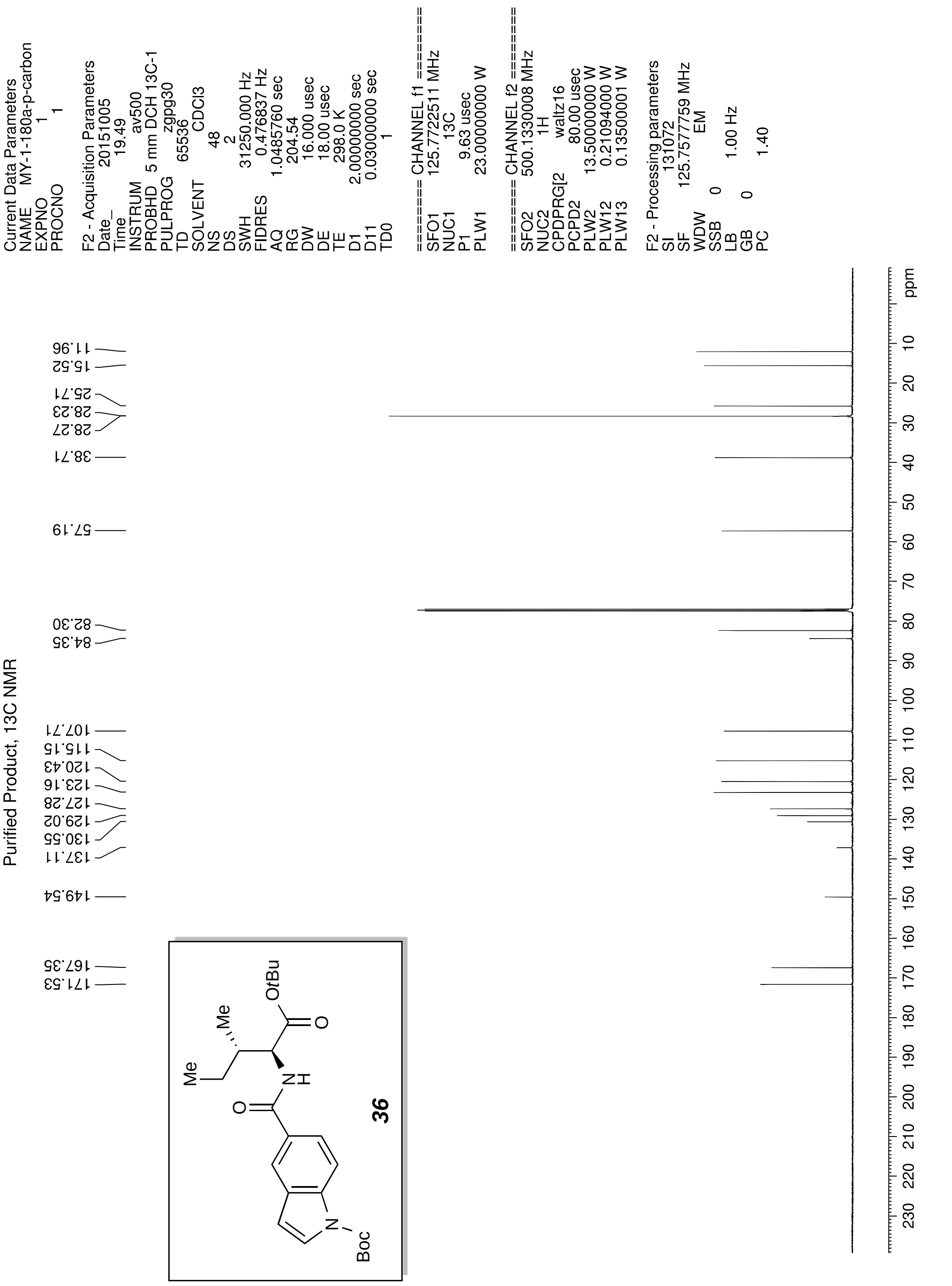


**Supplementary Figure 40. $^{13}$C NMR (500 MHz, $CDCl_3$) of 36**

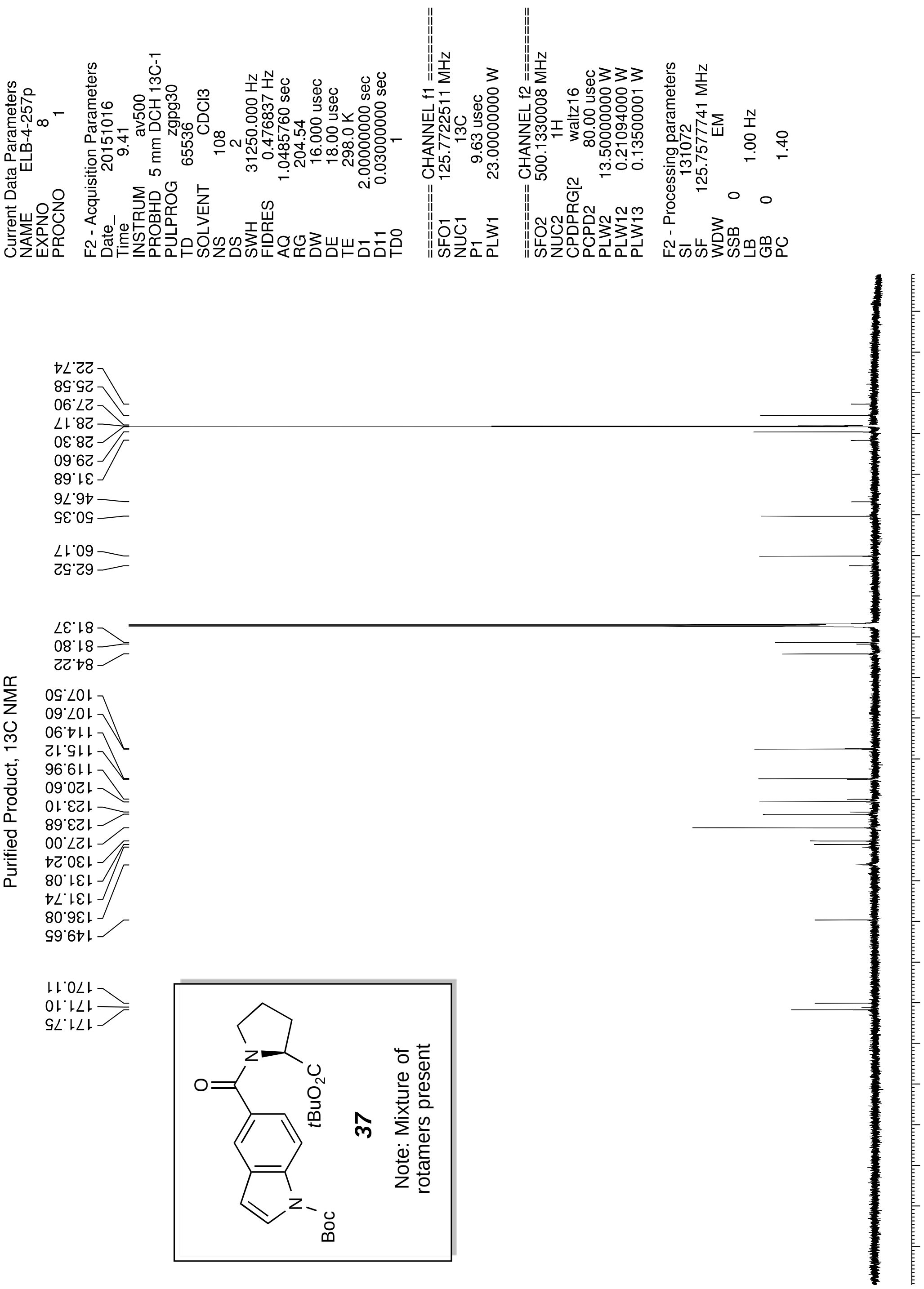


**Supplementary Figure 41. $^{13}$C NMR (500 MHz, $CDCl_3$) of 37**

| Compound | Method Column /Temp | Polar Cosolvent | Method Flow Rate | Rentention Times | Enantiomeric Ratio (er) |
|---|---|---|---|---|---|
| 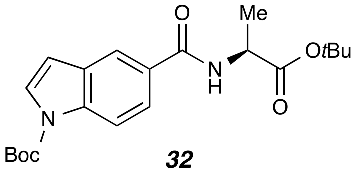 ***32*** | Daicel ChiralPak OJ-H / 35 °C | 10% MeOH | 2.00 mL/min | 3.9/4.52 min | **100:0** |
| 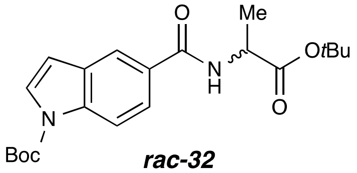 ***rac-32*** | Daicel ChiralPak OJ-H / 35 °C | 10% MeOH | 2.00 mL/min | 3.28/4.65 min | 50:50 |

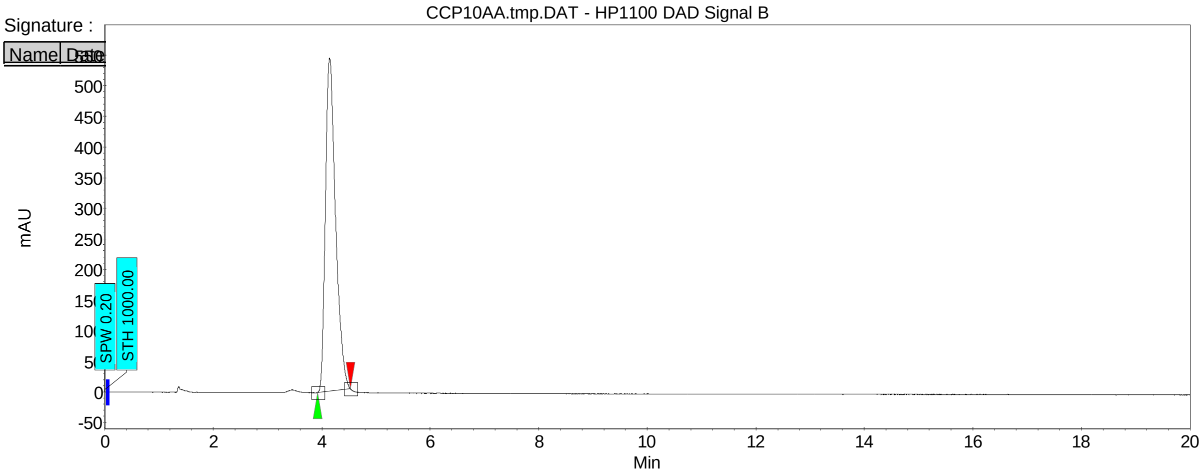


| Index | Name | Start | Time | End | RT Offset | Quantity | Height | Area | Area |
|---|---|---|---|---|---|---|---|---|---|
| | | [Min] | [Min] | [Min] | [Min] | [% Area] | [µV] | [µV.Min] | [%] |
| 1 | UNKNOWN | 3.92 | 4.14 | 4.52 | 0.00 | 100.00 | 543.9 | 117.8 | 100.000 |
| | | | | | | | | | |
| Total | | | | | | 100.00 | 543.9 | 117.8 | 100.000 |

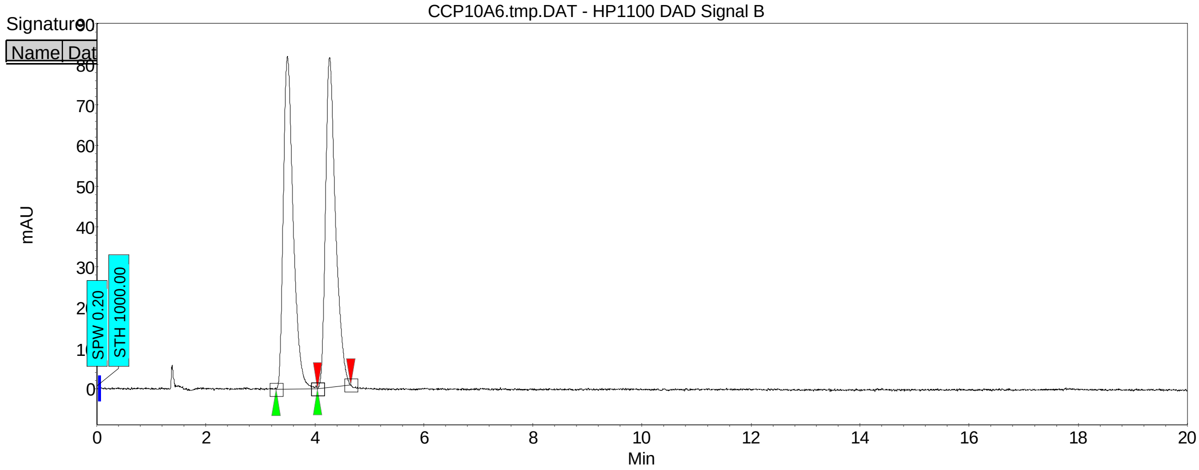


| Index | Name | Start | Time | End | RT Offset | Quantity | Height | Area | Area |
|---|---|---|---|---|---|---|---|---|---|
| | | [Min] | [Min] | [Min] | [Min] | [% Area] | [µV] | [µV.Min] | [%] |
| 1 | UNKNOWN | 3.28 | 3.50 | 4.05 | 0.00 | 49.71 | 82.1 | 17.1 | 49.706 |
| 2 | UNKNOWN | 4.05 | 4.27 | 4.65 | 0.00 | 50.29 | 81.4 | 17.3 | 50.294 |
| | | | | | | | | | |
| Total | | | | | | 100.00 | 163.5 | 34.4 | 100.000 |

**Supplementary Figure 42.** Enantioenriched (upper) and racemic (under) HPLC traces for **32**

| **Compound** | **Method Column /Temp** | **Polar Cosolvent** | **Method Flow Rate** | **Rentention Times** | **Enantiomeric Ratio (er)** |
|---|---|---|---|---|---|
| 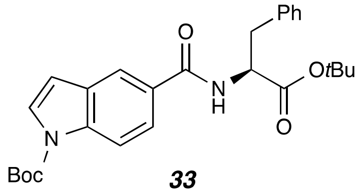 ***33*** | Daicel ChiralPak OJ-H / 35 °C | 10% MeOH | 2.00 mL/min | 7.58/8.77 min | **100:0** |
| 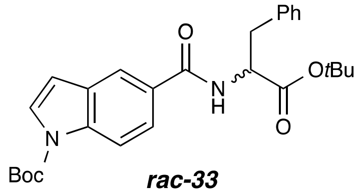 ***rac-33*** | Daicel ChiralPak OJ-H / 35 °C | 10% MeOH | 2.00 mL/min | 6.27/8.68 min | 48:52 |

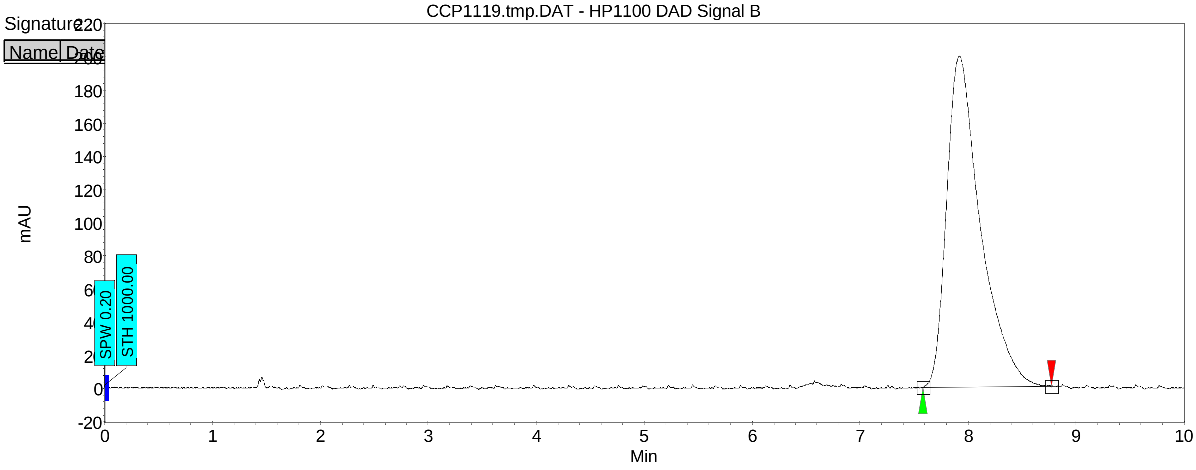


| Index | Name | Start | Time | End | RT Offset | Quantity | Height | Area | Area |
|---|---|---|---|---|---|---|---|---|---|
| | | [Min] | [Min] | [Min] | [Min] | [% Area] | [µV] | [µV.Min] | [%] |
| 1 | UNKNOWN | 7.58 | 7.92 | 8.77 | 0.00 | 100.00 | 199.5 | 72.4 | 100.000 |
| | | | | | | | | | |
| Total | | | | | | 100.00 | 199.5 | 72.4 | 100.000 |

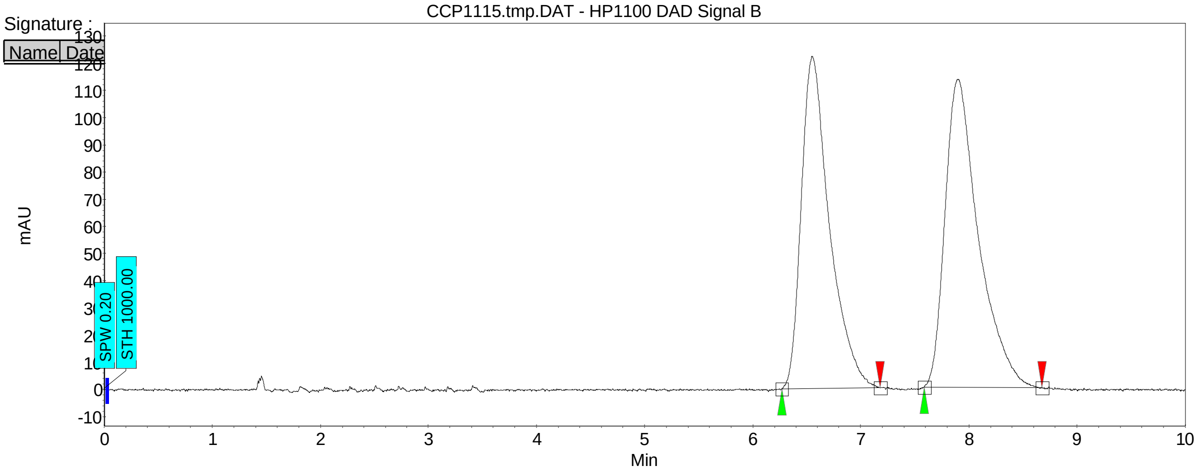


| Index | Name | Start | Time | End | RT Offset | Quantity | Height | Area | Area |
|---|---|---|---|---|---|---|---|---|---|
| | | [Min] | [Min] | [Min] | [Min] | [% Area] | [µV] | [µV.Min] | [%] |
| 1 | UNKNOWN | 6.27 | 6.55 | 7.18 | 0.00 | 48.01 | 122.1 | 37.5 | 48.007 |
| 2 | UNKNOWN | 7.59 | 7.90 | 8.68 | 0.00 | 51.99 | 113.3 | 40.6 | 51.993 |
| | | | | | | | | | |
| Total | | | | | | 100.00 | 235.4 | 78.0 | 100.000 |

**Supplementary Figure 43.** Enantioenriched (upper) and racemic (under) HPLC traces for **33**

| Compound | Method Column /Temp | Polar Cosolvent | Method Flow Rate | Rentention Times | Enantiomeric Ratio (er) |
|---|---|---|---|---|---|
| 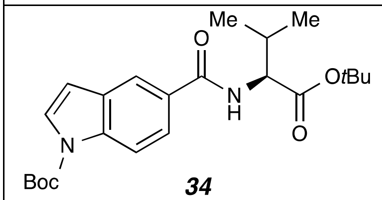  ***34*** | Daicel ChiralPak OJ-H / 35 °C | 10% MeOH | 2.00 mL/min | 3.56/4.13 min | **100:0** |
| 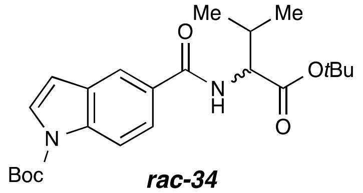  ***rac-34*** | Daicel ChiralPak OJ-H / 35 °C | 10% MeOH | 2.00 mL/min | 2.96/4.19 min | 50:50 |

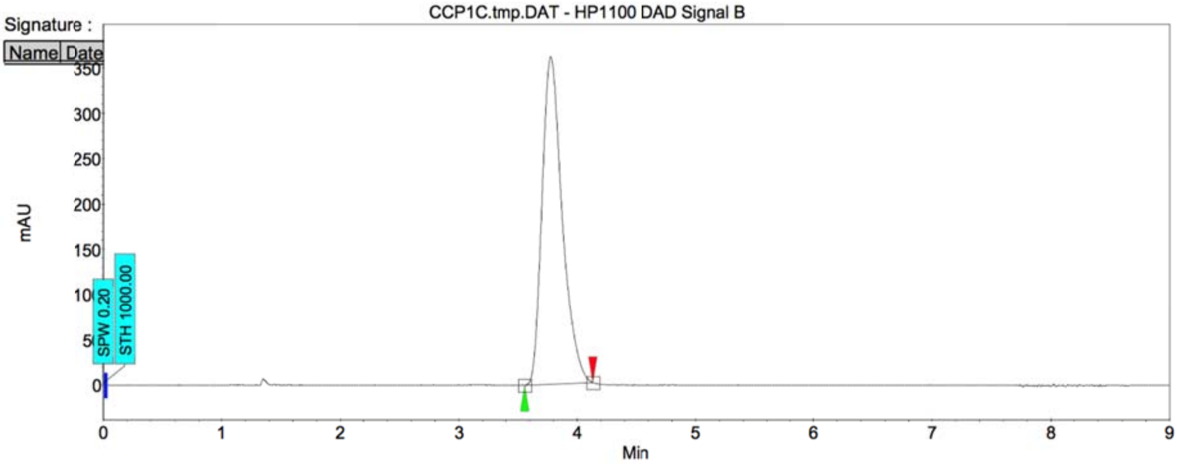


| Index | Name | Start | Time | End | RT Offset | Quantity | Height | Area | Area |
|---|---|---|---|---|---|---|---|---|---|
| | | [Min] | [Min] | [Min] | [Min] | [% Area] | [µV] | [µV.Min] | [%] |
| 1 | UNKNOWN | 3.56 | 3.78 | 4.13 | 0.00 | 100.00 | 361.4 | 71.1 | 100.000 |
| | | | | | | | | | |
| Total | | | | | | 100.00 | 361.4 | 71.1 | 100.000 |

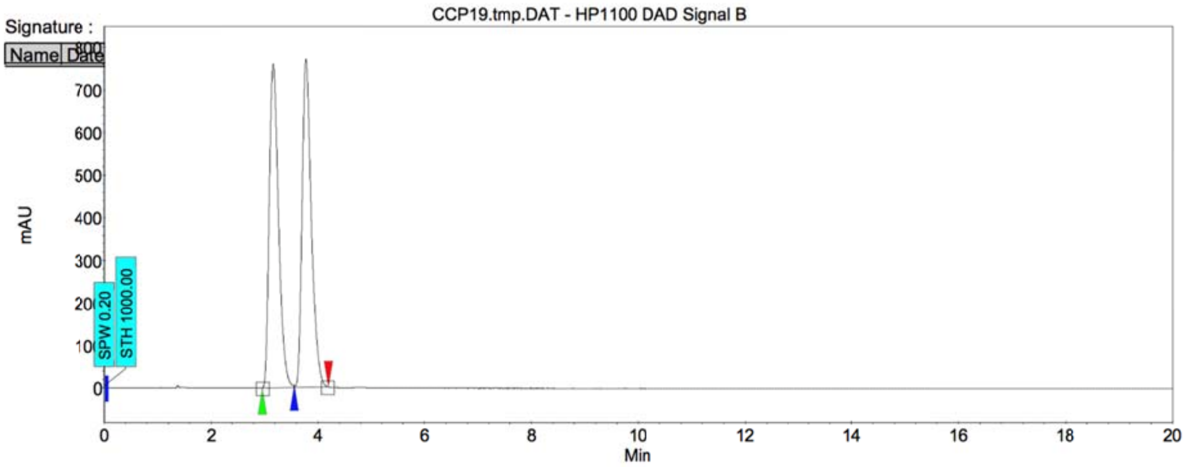


| Index | Name | Start | Time | End | RT Offset | Quantity | Height | Area | Area |
|---|---|---|---|---|---|---|---|---|---|
| | | [Min] | [Min] | [Min] | [Min] | [% Area] | [µV] | [µV.Min] | [%] |
| 1 | UNKNOWN | 2.96 | 3.17 | 3.56 | 0.00 | 49.82 | 760.3 | 152.2 | 49.823 |
| 2 | UNKNOWN | 3.56 | 3.78 | 4.19 | 0.00 | 50.18 | 769.6 | 153.3 | 50.177 |
| | | | | | | | | | |
| Total | | | | | | 100.00 | 1529.9 | 305.5 | 100.000 |

**Supplementary Figure 44.** Enantioenriched (upper) and racemic (under) HPLC traces for **34**

| Compound | Method Column /Temp | Polar Cosolvent | Method Flow Rate | Rentention Times | Enantiomeric Ratio (er) |
|---|---|---|---|---|---|
| 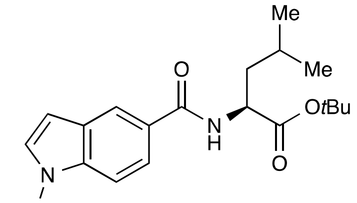 ***35*** | Daicel ChiralPak OJ-H / 35 °C | 10% MeOH | 2.00 mL/min | 4.59/5.24 min | **100:0** |
| 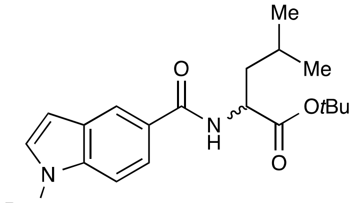 ***rac-35*** | Daicel ChiralPak OJ-H / 35 °C | 10% MeOH | 2.00 mL/min | 3.01/5.39 min | 50:50 |

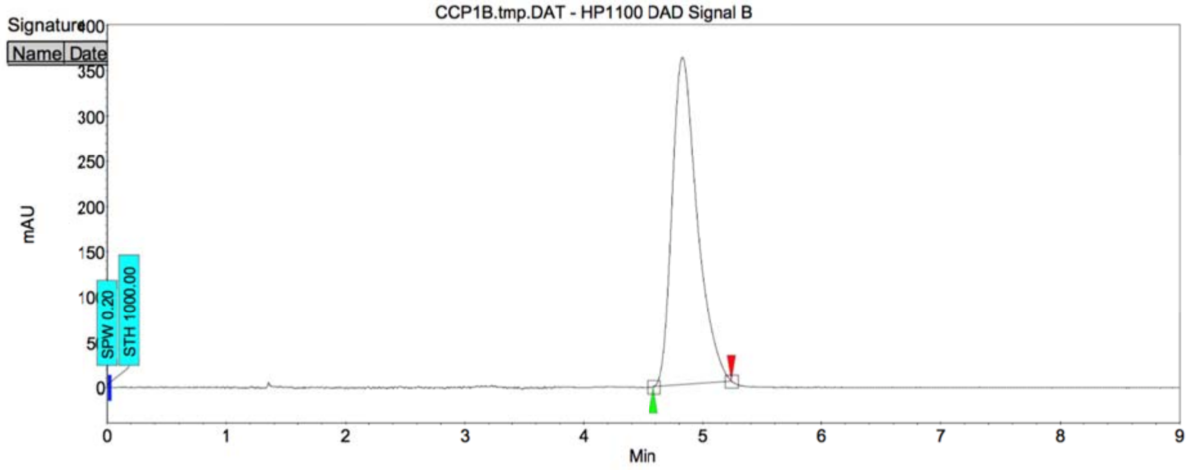


| Index | Name | Start | Time | End | RT Offset | Quantity | Height | Area | Area |
|---|---|---|---|---|---|---|---|---|---|
| | | [Min] | [Min] | [Min] | [Min] | [% Area] | [µV] | [µV.Min] | [%] |
| 1 | UNKNOWN | 4.59 | 4.83 | 5.24 | 0.00 | 100.00 | 361.8 | 87.9 | 100.000 |
| | | | | | | | | | |
| Total | | | | | | 100.00 | 361.8 | 87.9 | 100.000 |

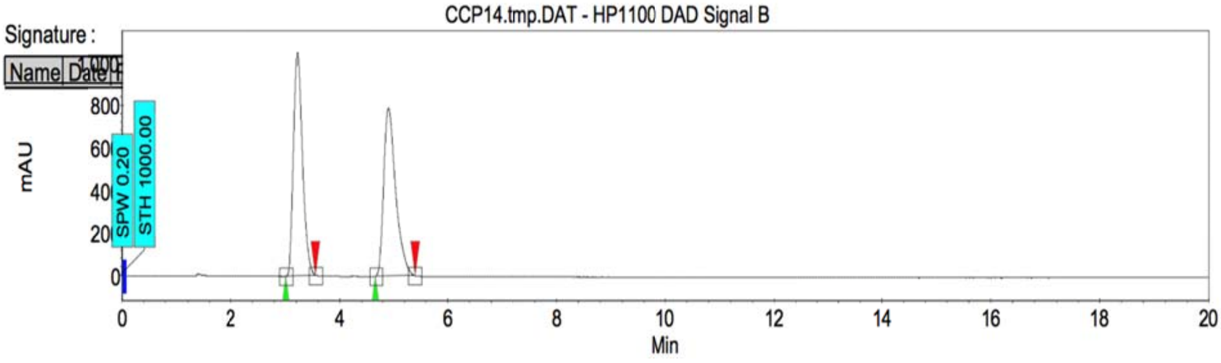


| Index | Name | Start | Time | End | RT Offset | Quantity | Height | Area | Area |
|---|---|---|---|---|---|---|---|---|---|
| | | [Min] | [Min] | [Min] | [Min] | [% Area] | [µV] | [µV.Min] | [%] |
| 1 | UNKNOWN | 3.01 | 3.23 | 3.56 | 0.00 | 50.21 | 1047.8 | 201.7 | 50.212 |
| 2 | UNKNOWN | 4.67 | 4.91 | 5.39 | 0.00 | 49.79 | 791.9 | 200.0 | 49.788 |
| | | | | | | | | | |
| Total | | | | | | 100.00 | 1839.7 | 401.7 | 100.000 |

**Supplementary Figure 45.** Enantioenriched (upper) and racemic (under) HPLC traces for **35**

| Compound | Method Column /Temp | Polar Cosolvent | Method Flow Rate | Rentention Times | Enantiomeric Ratio (er) |
|---|---|---|---|---|---|
| 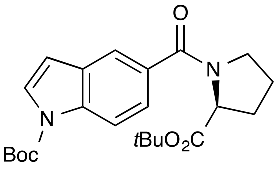  ***37*** | Daicel ChiralPak OJ-H / 35 °C | 5% MeOH | 2.00 mL/min | 10.49/12.33 min | **100:0** |
| 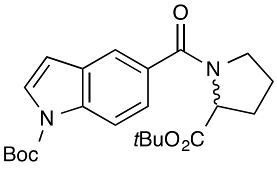  ***rac-37*** | Daicel ChiralPak OJ-H / 35 °C | 5% MeOH | 2.00 mL/min | 10.41/15.19 min | 50:50 |

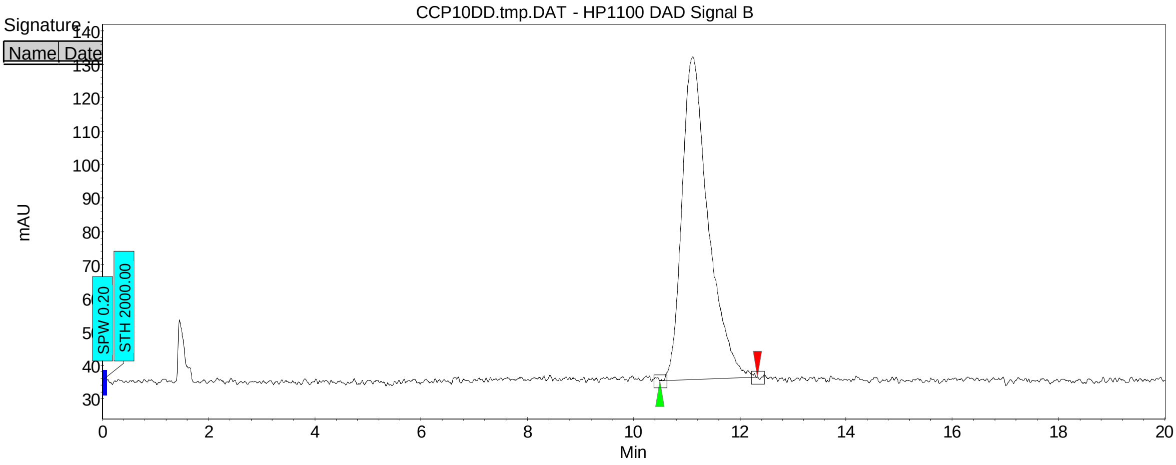


| Index | Name | Start | Time | End | RT Offset | Quantity | Height | Area | Area |
|---|---|---|---|---|---|---|---|---|---|
| | | [Min] | [Min] | [Min] | [Min] | [% Area] | [µV] | [µV.Min] | [%] |
| 1 | UNKNOWN | 10.49 | 11.11 | 12.33 | 0.00 | 100.00 | 96.4 | 55.3 | 100.000 |
| | | | | | | | | | |
| Total | | | | | | 100.00 | 96.4 | 55.3 | 100.000 |

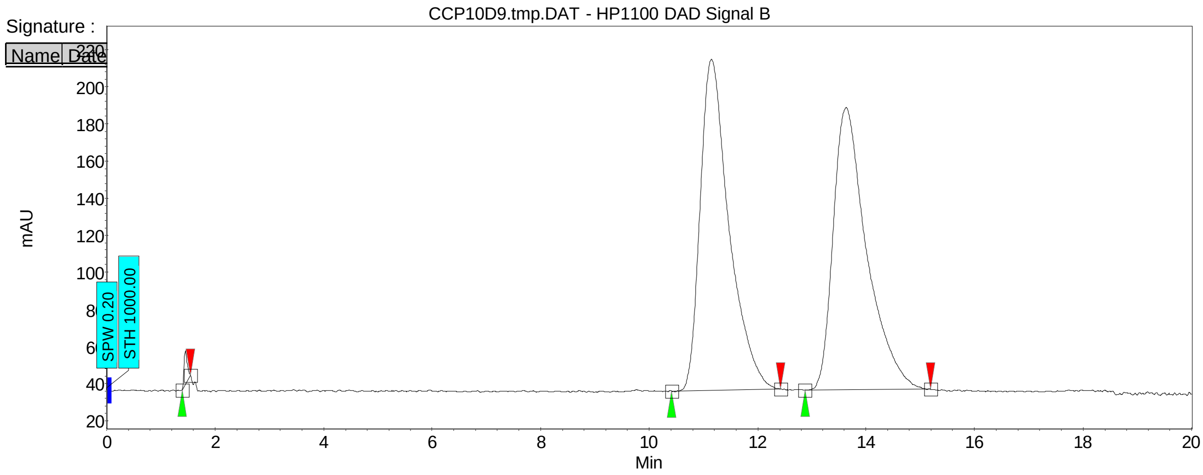


| Index | Name | Start | Time | End | RT Offset | Quantity | Height | Area | Area |
|---|---|---|---|---|---|---|---|---|---|
| | | [Min] | [Min] | [Min] | [Min] | [% Area] | [µV] | [µV.Min] | [%] |
| 1 | UNKNOWN | 1.39 | 1.45 | 1.55 | 0.00 | 0.56 | 17.9 | 1.2 | 0.560 |
| 2 | UNKNOWN | 10.41 | 11.15 | 12.42 | 0.00 | 49.94 | 178.3 | 109.6 | 49.941 |
| 3 | UNKNOWN | 12.87 | 13.63 | 15.19 | 0.00 | 49.50 | 152.0 | 108.6 | 49.499 |
| | | | | | | | | | |
| Total | | | | | | 100.00 | 348.2 | 219.4 | 100.000 |

**Supplementary Figure 46.** Enantioenriched (upper) and racemic (under) HPLC traces for **37**.

**Supplementary Table 1** Initial Survey of Benzamide Substrates with Morpholine (**9**).

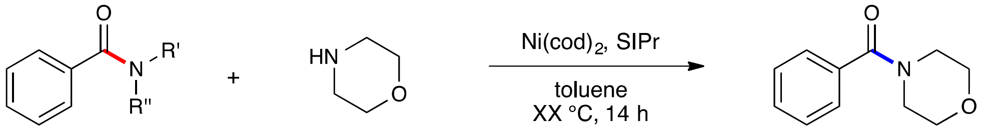


*9* *10*

| *Entry* | N(R')(R'') | *Ni(cod)$_2$ (mol%)* | *SIPr (mol%)* | *Equivs of 9* | *Temp (°C)* | *Yield of 10*[a] |
|---|---|---|---|---|---|---|
| 1 | N(Me)(H) *SI-8* | 10 | 20 | 2.5 | *60* | *0%* |
| 2 | N(Me)(Me) *SI-9* | 10 | 20 | 2.5 | *60* | *0%* |
| 3 | N(Ph)(Me) *SI-10* | 10 | 20 | 2.5 | *60* | *88%* |
| 4 | N(Bn)(Boc) *8b* | 10 | 20 | 2.5 | *60* | *quantitative yield* |
| 5 | N(Bn)(Boc) *8b* | 10 | 10 | 1.5 | *35* | *91%*[b] |
| 6 | N(Bn)(Boc) *8b* | – | 10 | 1.5 | *35* | *0%* |
| 7 | N(Bn)(Boc) *8b* | – | – | 1.5 | *35* | *0%* |

[a] Yields were determined by $^1$H NMR analysis using 1,3,5-trimethoxybenzene as an internal standard. [b] Yield range based on two isolation experiments.

## Supplementary Methods

### General

Unless stated otherwise, reactions were conducted in flame-dried glassware under an atmosphere of nitrogen and commercially obtained reagents were used as received. Non-commercially available substrates were synthesized following protocols specified beginning on page S46. Toluene was purified by distillation and taken through five freeze-pump-thaw cycles. Acid chlorides **SI-1**, **SI-5**, carboxylic acid **SI-3**, amines benzylamine, aniline (**SI-18**), 4-methoxyaniline (**SI-19**), 2,6-dimethylaniline (**SI-21**), adamantyl-1-amine (**SI-23**), 2-methylimidazoline (**SI-24**), 1-(2-pyridinyl)-piperazine (**SI-26**), were obtained from Sigma–Aldrich. Morpholine (**9**) was obtained from Spectrum Chemical MFG Corp., 2-biphenylylamine (**SI-20**) from Combi-Blocks and 3-amino-9-ethylcarbazole (**SI-25**) from Alfa Aesar. *N*-Methylbenzamide (**SI-8**) was purchased from Alfa Aesar. All liquid amines were distilled over $CaH_2$ prior to use. Natural amino esters L-alanine *tert*-butyl ester hydrochloride (**SI-33**), L-phenylalanine *tert*-butyl ester hydrochloride (**SI-35**), L-valine *tert*-butyl ester hydrochloride (**SI-37**), L-leucine *tert*-butyl ester hydrochloride (**SI-39**), L-isoleucine *tert*-butyl ester hydrochloride and L-proline *tert*-butyl ester hydrochloride (**SI-41**) were obtained from Combi-Blocks. Unnatural amino esters D-phenylalanine *tert*-butyl ester hydrochloride (**SI-36**), D-leucine *tert*-butyl ester hydrochloride (**SI-40**), and D-proline *tert*-butyl ester hydrochloride (**SI-42**) were obtained from Chem-Impex. D-alanine *tert*-butyl ester hydrochloride (**SI-34**) and D-valine *tert*-butyl ester hydrochloride (**SI-38**) was obtained from Combi-Blocks. Amberlyst® A21 free base was obtained from Sigma-Aldrich. $Ni(cod)_2$ and SIPr were obtained from Strem Chemicals. Ligand **SI-41** was obtained from Stream Chemicals, Inc. Reaction temperatures were controlled using an IKAmag temperature modulator, and unless stated otherwise, reactions were performed at room temperature (approximately 23 °C). Thin-layer chromatography (TLC) was conducted with EMD gel 60 F254 pre-coated plates (0.25 mm for analytical chromatography and 0.50 mm for preparative chromatography) and visualized using a combination of UV, anisaldehyde, and potassium permanganate staining techniques. Silicycle Siliaflash P60 (particle size 0.040–0.063 mm) was used for flash column chromatography. $^1H$ NMR spectra were recorded on Bruker spectrometers (at 500 MHz) and are reported relative to residual solvent signals. Data for $^1H$ NMR spectra are reported as follows: chemical shift (δ ppm), multiplicity, coupling constant (Hz), integration. Data for $^{13}C$ NMR are reported in terms of chemical shift (at 125 MHz). Data for $^{19}F$ NMR are reported in terms of chemical shift (at 282 MHz). IR spectra were recorded on a

Perkin-Elmer UATR Two FT-IR spectrometer and are reported in terms of frequency absorption ($cm^{-1}$). High-resolution mass spectra were obtained on Thermo Scientific™ Exactive Mass Spectrometer with DART ID-CUBE. Determination of enantiopurity was carried out on a Mettler Toledo SFC (supercritical fluid chromatography) using a Daicel ChiralPak OJ-H column.

**Preparation of Amide Substrates**

The following amides shown in Figure 2 and Supplementary Table S1 were synthesized following known protocols: **8b**[1], **SI-9**[2], **SI-10**[3], **SI-11**[1], **SI-12**[1], **SI-13**[1], **SI-14**[1], **SI-15**[1], **SI-16**[1], **SI-22**[4]. Syntheses for the remaining substrates shown in Figure 3 and 4 are as follows:

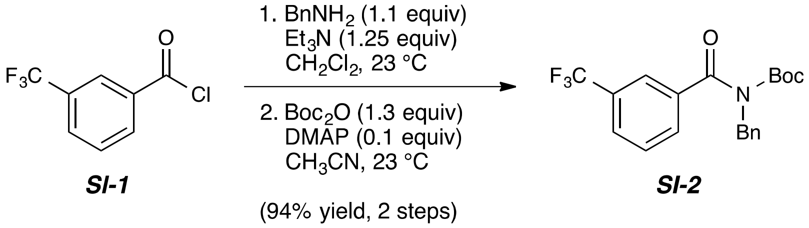


**Amide SI-2.** To a solution of acid chloride **SI-1** (1.38 g, 6.62 mmol, 1.0 equiv) and triethylamine (1.14 mL, 8.28 mmol, 1.25 equiv) in dichloromethane (6.6 mL), was added dropwise a solution of benzylamine (0.794 mL, 5.77 mmol, 1.1 equiv) in dichloromethane (6.6 mL, 0.5 M in total). The reaction mixture was stirred at 23 °C for 1 h, then diluted with EtOAc (50 mL) and washed successively with 1.0 M HCl (50 mL) and brine (50 mL). The organic layer was dried over $Na_2SO_4$ and concentrated under reduced pressure. The resulting crude solid material was used in the subsequent step without further purification.

To a flask containing the crude material from the previous step was added DMAP (80.8 mg, 0.662 mmol, 0.1 equiv) followed by acetonitrile (33.1 mL, 0.2 M). $Boc_2O$ (1.88 g, 8.61 mmol, 1.3 equiv) was added in one portion and the reaction vessel was flushed with $N_2$, then the reaction mixture was allowed to stir at 23 °C for 16 h. The reaction was quenched by the addition of saturated aqueous $NaHCO_3$ (10 mL), transferred to a separatory funnel with EtOAc (30 mL) and $H_2O$ (30 mL), and extracted with EtOAc (3 x 20 mL). The organic layers were combined, dried over $Na_2SO_4$, and evaporated under reduced pressure. The resulting crude residue was purified by flash chromatography (19:1 Hexanes:EtOAc) to yield amide **SI-2** (2.36 g, 94% yield, over two steps) as a white solid. Amide **SI-2**: mp: 67.8–69.7 °C; $R_f$ 0.54 (5:1 Hexanes:Acetone);

$^1$H NMR (500 MHz, $CDCl_3$): δ 7.78–7.74 (m, 1H), 7.74–7.66 (m, 2H), 7.56–7.49 (m, 1H), 7.45–7.39 (m, 2H), 7.37–7.31 (m, 2H), 7.31–7.25 (m, 1H), 5.00 (s, 2H), 1.14 (s, 9H); $^{13}$C NMR (125 MHz, $CDCl_3$): δ 171.7, 153.1, 138.6, 137.6, 130.8 (q, $J$ = 32.3), 130.7, 128.9, 128.7, 128.3, 127.7, 127.6 (q, $J$ = 3.6), 123.8 (q, $J$ = 270.3), 124.4 (q, $J$ = 3.9), 84.0, 49.1, 27.5; $^{19}$F NMR (282 MHz, $CDCl_3$): δ –62.8; IR (film): 3035, 2982, 1735, 1678, 1321, 1141 cm$^{-1}$; HRMS-ESI (*m/z*) $[M + H]^+$ calcd for $C_{20}H_{21}F_3NO_3$, 380.1474; found 380.1449.

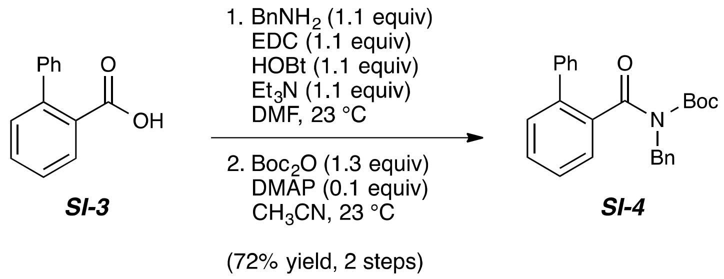


**Amide SI-4.** To a mixture of carboxylic acid **SI-3** (1.14 g, 5.78 mmol, 1.0 equiv), EDC (1.22 g, 6.36 mmol, 1.1 equiv), HOBt (858.0 mg, 6.36 mmol, 1.1 equiv) and triethylamine (0.880 mL, 6.36 mmol, 1.1 equiv) in DMF (57.8 mL, 1.0 M) was added benzylamine (0.694 mL, 6.36 mmol, 1.1 equiv). The resulting mixture was stirred at 23 °C for 16 h, and then diluted with deionized water (50 mL) and transferred to a separatory funnel with EtOAc (50 mL) and brine (50 mL). The aqueous layer was extracted with EtOAc (3 x 30 mL), and then the organic layers were combined and washed with deionized water (3 x 50 mL), dried over $Na_2SO_4$, and evaporated under reduced pressure. The resulting crude solid material was used in the subsequent step without further purification.

To a flask containing the crude material from the previous step was added DMAP (70.8 mg, 0.58 mmol, 0.1 equiv) followed by acetonitrile (28.9 mL, 0.2 M). $Boc_2O$ (1.64 g, 7.51 mmol, 1.3 equiv) was added in one portion and the reaction vessel was flushed with $N_2$, and then the reaction mixture was allowed to stir at 23 °C for 16 h. The reaction was quenched by the addition of saturated aqueous $NaHCO_3$ (10 mL), transferred to a separatory funnel with EtOAc (30 mL) and $H_2O$ (30 mL), and extracted with EtOAc (3 x 20 mL). The organic layers were combined, dried over $Na_2SO_4$, and evaporated under reduced pressure. The resulting crude residue was purified by flash chromatography (24:1 Hexanes:EtOAc) to yield amide **SI-4** (1.60 g, 72% yield,

over two steps) as an off-white solid. Amide **SI-4**: mp: 80.7–82.5 ºC; $R_f$ 0.38 (5:1 Hexanes:EtOAc); $^1$H NMR (500 MHz, $CDCl_3$): δ 7.51–7.43 (m, 1H), 7.41–7.32 (m, 8H), 7.31–7.21 (m, 5H), 5.03–4.47 (m, 2H), 1.09 (s, 9H); $^{13}$C NMR (125 MHz, $CDCl_3$): δ 172.8, 152.4, 140.1, 139.2, 137.9, 137.8, 130.0, 129.7, 128.8, 128.5, 128.4, 128.3, 127.7, 127.4, 127.1, 126.9, 83.4, 48.2, 27.6; IR (film): 3063, 2979, 1732, 1671, 1369, 1225 cm$^{-1}$; HRMS-ESI (*m/z*) $[M + H]^+$ calcd for $C_{25}H_{26}NO_3$ 388.1913; found 388.1907.

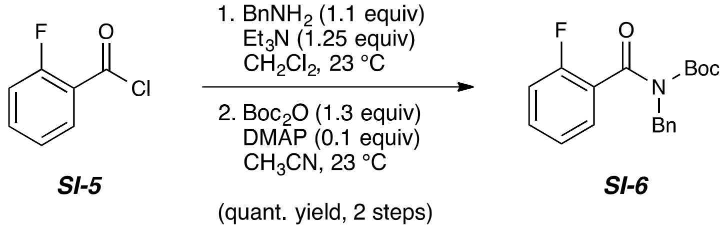


***SI-5*** ***SI-6***

**Amide SI-6.** To a solution of acid chloride **SI-5** (0.772 mL, 6.45 mmol, 1.0 equiv) and triethylamine (1.12 mL, 8.06 mmol, 1.25 equiv) in dichloromethane (6.5 mL), was added dropwise a solution of benzylamine (0.774 mL, 7.10 mmol, 1.1 equiv) in dichloromethane (6.5 mL, 0.5 M in total). The reaction mixture was stirred at 23 °C for 1 h, then diluted with EtOAc (50 mL) and washed successively with 1.0 M HCl (50 mL) and brine (50 mL). The organic layer was dried over $Na_2SO_4$ and concentrated under reduced pressure. The resulting crude solid material was used in the subsequent step without further purification.

To a flask containing the crude material from the previous step was added DMAP (79.0 mg, 0.645 mmol, 0.1 equiv) followed by acetonitrile (32.0 mL, 0.2 M). $Boc_2O$ (1.83 g, 8.39 mmol, 1.3 equiv) was added in one portion and the reaction vessel was flushed with $N_2$, then the reaction mixture was allowed to stir at 23 °C for 16 h. The reaction was quenched by the addition of saturated aqueous $NaHCO_3$ (10 mL), transferred to a separatory funnel with EtOAc (30 mL) and $H_2O$ (30 mL), and extracted with EtOAc (3 x 20 mL). The organic layers were combined, dried over $Na_2SO_4$, and evaporated under reduced pressure. Purification by flash chromatography (9:1 Hexanes:EtOAc) generated amide **SI-6** (quant. yield, over two steps) as an off-white solid. Amide **SI-6**: mp: 67.5–69.8 ºC; $R_f$ 0.39 (5:1 Hexanes:EtOAc); $^1$H NMR (500 MHz, $CDCl_3$): δ 7.57–7.48 (m, 1H), 7.46–7.38 (m, 3H), 7.36–7.30 (m, 2H), 7.30–7.23 (m, 1H),

7.22–7.14 (m, 1H), 7.08–6.99 (m, 1H), 5.04 (s, 2H), 1.19 (s, 9H); $^{13}$C NMR (125 MHz, $CDCl_3$): δ 168.0, 159.8, 157.8, 152.8, 137.7, 132.2 (d, $J$ = 8.4), 129.7 (d, $J$ = 2.8), 128.5, 128.1, 127.5, 126.5 (d, $J$ = 14.6), 124.4 (d, $J$ = 3.4), 115.4 (d, $J$ = 22.5), 83.7, 48.5, 27.5; $^{19}$F NMR (282 MHz, $CDCl_3$): δ –62.8; IR (film): 3035, 2981, 1737, 1670, 1455, 1352 cm$^{-1}$; HRMS-ESI (*m/z*) [M + H]$^+$ calcd for $C_{19}H_{21}FNO_3$, 330.1505; found 330.1487.

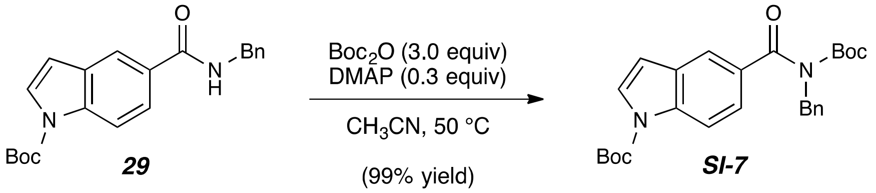


**Amide SI-7**. To a flask containing amide **29** (prepared from the known carboxylic acid[5]) (100 mg, 0.285 mmol, 1.0 equiv) was added DMAP (10.4 mg, 0.86 mmol, 0.3 equiv) followed by acetonitrile (1.4 mL, 0.2 M). $Boc_2O$ (186 mg, 0.86 mmol, 3.0 equiv) was added in one portion. The reaction vessel was flushed with $N_2$, and then the reaction mixture was heated to 50 °C. After stirring for 19 h, the reaction was quenched by the addition of saturated aqueous $NaHCO_3$ (2 mL), transferred to a separatory funnel with EtOAc (2 mL) and $H_2O$ (2 mL), and extracted with EtOAc (3 x 10 mL). The organic layers were combined, dried over $Na_2SO_4$, and evaporated under reduced pressure. The resulting crude residue was purified by flash chromatography (20:1 → 10:1 Hexanes:EtOAc) to yield amide **SI-7** (127 mg, 99% yield) as a white solid. Amide **SI-7**: mp: 112.4–114.5 ºC; $R_f$ 0.63 (3:1 Hexanes:EtOAc); $^1$H NMR (500 MHz, $CDCl_3$): δ 8.19–8.09 (m, 1H), 7.08–7.76 (m, 1H), 7.65–7.60 (m, 1H), 7.54–7.48 (m, 1H), 7.47–7.42 (m, 2H), 7.37–7.30 (m, 2H), 7.29–7.23 (m, 1H), 6.61–6.56 (m, 1H), 5.00 (s, 2H), 1.68 (s, 9H), 1.10 (s, 9H); $^{13}$C NMR (125 MHz, $CDCl_3$): δ 173.6, 153.9, 149.6, 138.2, 136.9, 132.1, 130.1, 128.6, 128.3, 127.5, 127.3, 124.2, 121.3, 114.8, 107.7, 84.4, 83.1, 49.3, 28.3, 27.6; IR (film): 2979, 1731, 1671, 1368, 1334 cm$^{-1}$; HRMS-ESI (*m/z*) [M + H]$^+$ calcd for $C_{26}H_{31}N_2O_5$, 451.2233; found 451.2193.

**Initial Survey and Optimization of Benzamide Substrates with Morpholine (9)**

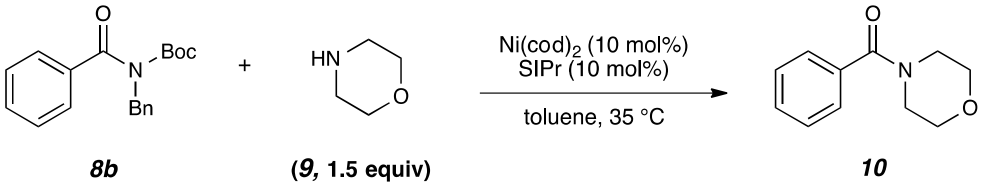


**Representative procedure for transamidation reactions of benzamides from Figure 2 and Supplementary Table 1 (coupling of amide 8b and morpholine (9) is used as an example).** A 1-dram vial containing a magnetic stir bar was flame-dried under reduced pressure, and then allowed to cool under $N_2$. The vial was charged with amide substrate **8b** (62.2 mg, 0.200 mmol, 1.0 equiv), and 1,3,5-trimethoxybenzene (10.1 mg, 0.060 mmol, 0.3 equiv) and the vial was flushed with $N_2$. Morpholine (**9**) (37.0 μL, 0.300 mmol, 1.5 equiv) was then added to the vial, which was then taken into a glove box and charged with Ni(cod)$_2$ (5.5 mg, 0.020 mmol, 10 mol%) and SIPr (7.8 mg, 0.020 mmol, 10 mol%). Subsequently, toluene (0.20 mL, 1.0 M) was added. The vial was sealed with a Teflon-lined screw cap, removed from the glove box, and stirred at 35 °C for 14 h. After cooling to 23 °C, the mixture was diluted with hexanes (0.5 mL) and filtered by passage through a plug of silica gel (10 mL of EtOAc eluent). The volatiles were removed under reduced pressure, and the yield was determined by $^1$H NMR analysis with 1,3,5-trimethoxybenzene as an internal standard.[6]

**Procedure for Activated Amide Transamidation via Nickel-Catalysis**

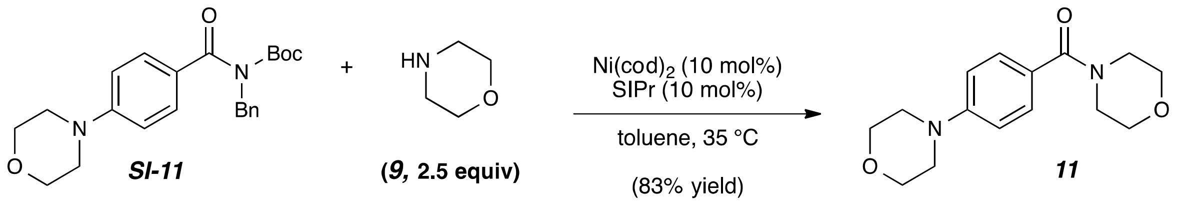


**Representative Procedure (coupling of amide SI-11 and morpholine (9) is used as an example). Amide 11 (Figure 3 entry 1).** A 1-dram vial containing a magnetic stir bar was flame-dried under reduced pressure, and then allowed to cool under $N_2$. The vial was charged with amide substrate **SI-11** (79.3 mg, 0.200 mmol, 1.0 equiv), and the vial was flushed with $N_2$. Morpholine (**9**) (43.1 μL, 0.500 mmol, 2.5 equiv) was added to the vial, which was then taken into a glove box and charged with Ni(cod)$_2$ (5.5 mg, 0.020 mmol, 10 mol%) and SIPr (7.8 mg,

0.020 mmol, 10 mol%). Subsequently, toluene (0.20 mL, 1.0 M) was added. The vial was sealed with a Teflon-lined screw cap, removed from the glove box, and stirred at 35 °C for 14 h. After cooling to 23 °C, the mixture was diluted with hexanes (0.5 mL) and filtered over a plug of silica gel (10 mL of EtOAc eluent). The volatiles were removed under reduced pressure, and the crude residue was purified by preparative thin-layer chromatography (1:1 Hexanes:EtOAc → 100% EtOAc) to yield amide product **11** (83% yield, average of two experiments) as a white solid. Amide **11**: $R_f$ 0.32 (100% EtOAc). Spectral data match those previously reported.[7]

*Any modifications of the conditions shown in the representative procedure above are specified in the following schemes, which depict all of the results shown in Figure 3.*

*For each of the nickel-catalysed reactions described herein, control experiments were performed concurrently where Ni(cod)$_2$ or both Ni(cod)$_2$ and SIPr were omitted from the reactions. In all cases, these control experiments led to the recovery of the amide substrates with no detectable conversion to the corresponding amides.*

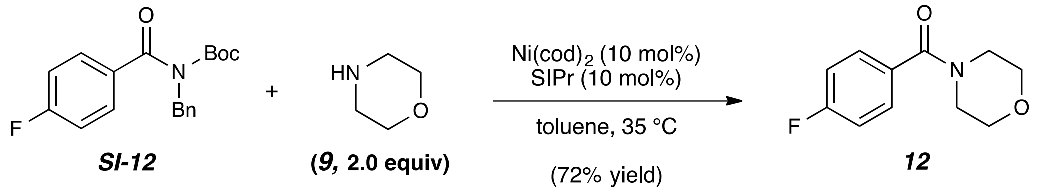


**Amide 12 (Figure 3 entry 2)**. Purification by flash chromatography (2:1 Hexanes:EtOAc) generated amide **12** (72% yield, average of two experiments) as a clear oil. Amide **12**: $R_f$ 0.31 (1:1 Hexanes:EtOAc). Spectral data match those previously reported.[8]

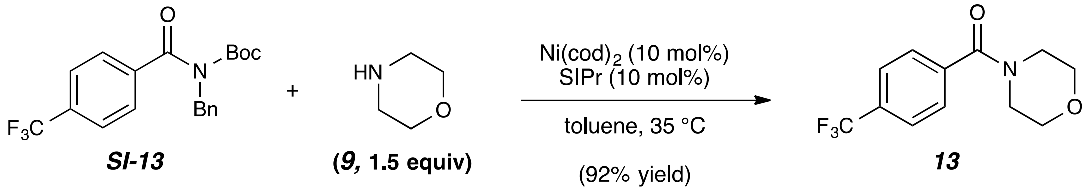

**Amide 13 (Figure 3 entry 3)**. Purification by flash chromatography (2:1 Hexanes:EtOAc) generated amide **13** (92% yield, average of two experiments) as a white solid. Amide **13**: $R_f$ 0.42 (1:1 Hexanes:EtOAc). Spectral data match those previously reported.[9]

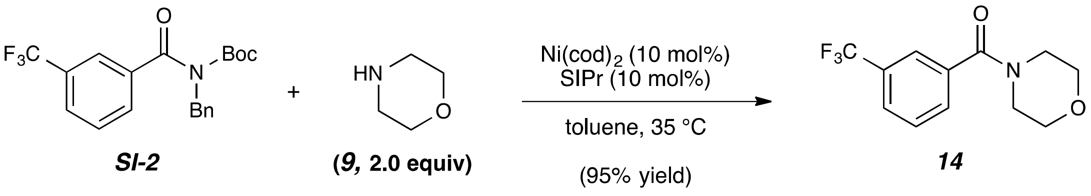


**Amide 14 (Figure 3 entry 4)**. Purification by flash chromatography (2:1 Hexanes:EtOAc) generated amide **14** (95% yield, average of two experiments) as a white solid. Amide **14**: $R_f$ 0.39 (1:1 Hexanes:EtOAc). Spectral data match those previously reported.[10]

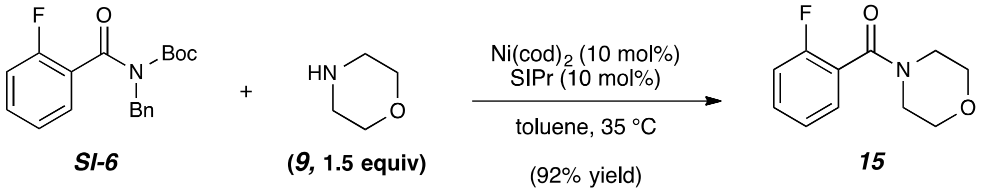


**Amide 15 (Figure 3 entry 5)**. Purification by flash chromatography (5:1 → 2:1 Hexanes:EtOAc) generated amide **15** (92% yield, average of two experiments) as a white solid. Amide **15**: $R_f$ 0.43 (1:1 Hexanes:EtOAc). Spectral data match those previously reported.[11]

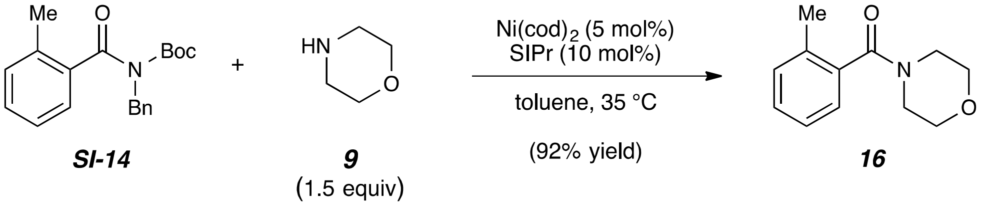


**Amide 16 (Figure 3 entry 6)**. Purification by flash chromatography (5:1 → 2:1 Hexanes:EtOAc) generated amide **16** (92% yield, average of two experiments) as a white

crystalline solid. Amide **16**: $R_f$ 0.41 (1:1 Hexanes:EtOAc). Spectral data match those previously reported.[9]

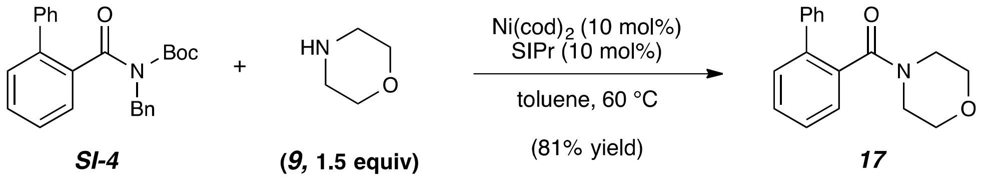


*SI-4* (*9*, 1.5 equiv) *17*

**Amide 17 (Figure 3 entry 7)**. Purification by flash chromatography (2:1 Hexanes:EtOAc) generated amide **17** (81% yield, average of two experiments) as a white solid. Amide **17**: $R_f$ 0.43 (1:1 Hexanes:EtOAc). Spectral data match those previously reported.[11]

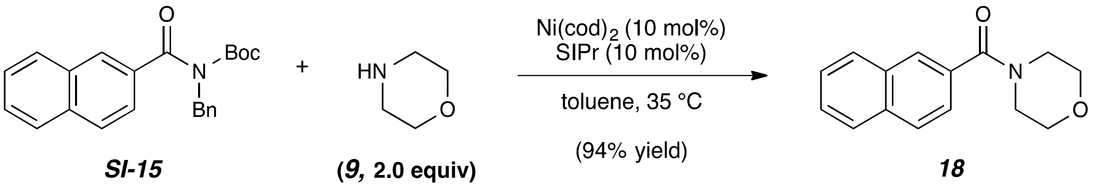


*SI-15* (*9*, 2.0 equiv) *18*

**Amide 18 (Figure 3 entry 8)**. Purification by flash chromatography (5:1 Hexanes:EtOAc) generated amide **18** (94% yield, average of two experiments) as a white solid. Amide **18**: $R_f$ 0.29 (1:1 Hexanes:EtOAc); $^1$H NMR (500 MHz, $CDCl_3$): δ 7.94–7.82 (m, 4H), 7.60–7.44 (m, 3H), 4.90–3.26 (m, 8H); $^{13}$C NMR (125 MHz, $CDCl_3$, 13 of 14 observed): δ 170.5, 133.8, 132.7, 132.6, 128.5, 128.4, 127.8, 127.2, 127.1, 126.8, 124.2, 66.9, 48.2, 42.7; IR (film): 3490, 2965, 2855, 1623, 1427 cm$^{-1}$; HRMS-ESI (*m/z*) $[M + H]^+$ calcd for $C_{15}H_{16}NO_2$, 242.1181; found 242.0291.

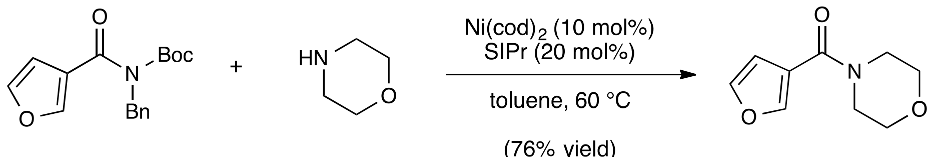


*SI-16* (*9*, 1.5 equiv) *19*

**Amide 19 (Figure 2 entry 9)**. Purification by flash chromatography (2:1 Hexanes:EtOAc) generated amide **19** (76% yield, average of two experiments) as a clear oil. Amide **19**: $R_f$ 0.25 (1:1 Hexanes:EtOAc). Spectral data match those previously reported.[12]

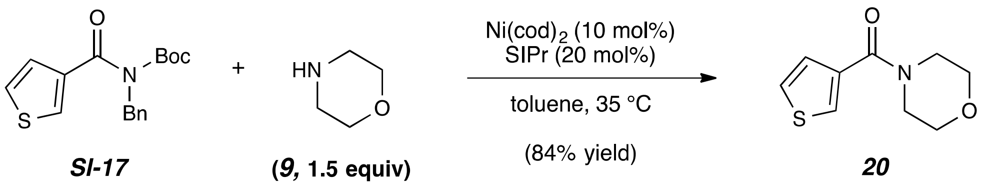


***SI-17*** (***9***, **1.5 equiv**) ***20***

**Amide 20 (Figure 3 entry 10)**. Purification by flash chromatography (2:1 Hexanes:EtOAc) generated amide **20** (84% yield, average of two experiments) as a clear oil. Amide **20**: $R_f$ 0.25 (1:1 Hexanes:EtOAc). Spectral data match those previously reported.[13]

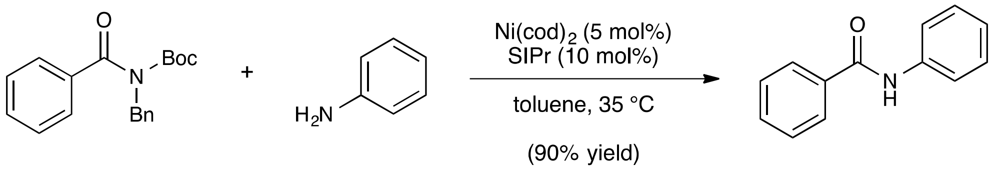


***8b*** (***SI-18***, **1.2 equiv**) ***21***

**Amide 21 (Figure 3 entry 11)**. Purification by flash chromatography (15:1 Hexanes:EtOAc) generated amide **21** (90% yield, average of two experiments) as a yellow solid. Amide **21**: $R_f$ 0.39 (3:1 Hexanes:EtOAc). Spectral data match those previously reported.[8]

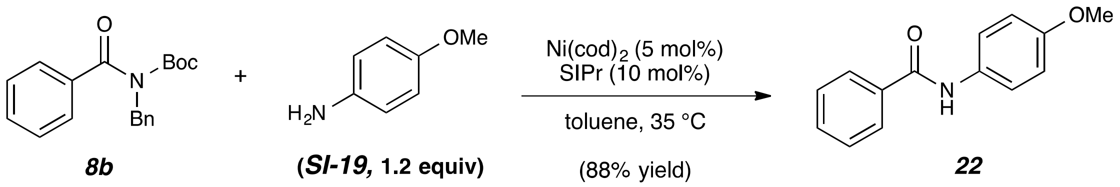


***8b*** (***SI-19***, **1.2 equiv**) ***22***

**Amide 22 (Figure 3 entry 12)**. Purification by flash chromatography (5:1 Hexanes:EtOAc) generated amide **22** (88% yield, average of two experiments) as an opaque solid. Amide **22**: $R_f$ 0.75 (1:1 Hexanes:EtOAc). Spectral data match those previously reported.[14]

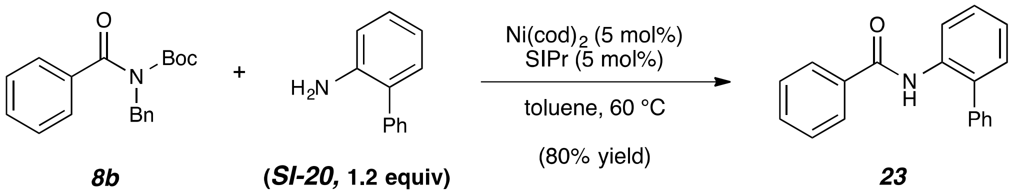


**Amide 23 (Figure 3 entry 13)**. Purification by flash chromatography (40:1 Hexanes:EtOAc) generated amide **23** (80% yield, average of two experiments) as an opaque oil. Amide **23**: $R_f$ 0.48 (5:1 Hexanes:EtOAc). Spectral data match those previously reported.[15]

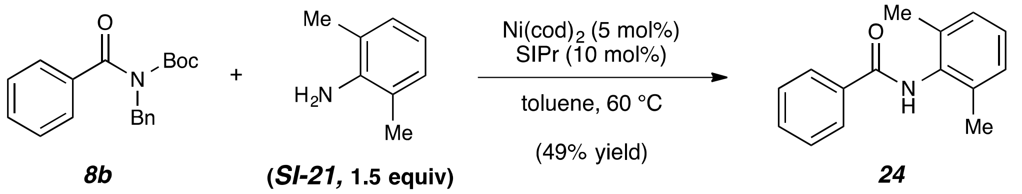


**Amide 24 (Figure 3 entry 14)**. Purification by flash chromatography (20:1 → 10:1 → 2:1 Hexanes:EtOAc) generated amide **24** (89% yield, average of two experiments) as a white solid. Amide **24**: $R_f$ 0.22 (5:1 Hexanes:EtOAc). Spectral data match those previously reported.[16]

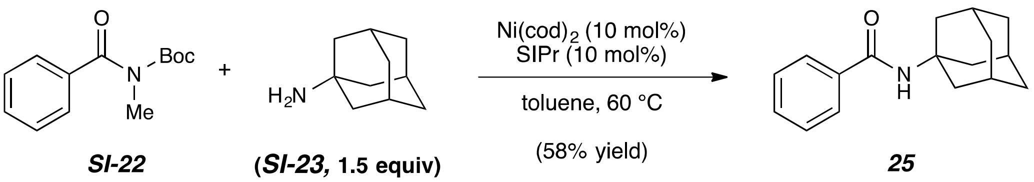


**Amide 25 (Figure 3 entry 15)**. Purification by flash chromatography (40:1 Hexanes:EtOAc) generated amide **25** (58% yield, average of two experiments) as a white solid. Amide **25**: $R_f$ 0.48 (5:1 Hexanes:EtOAc). Spectral data match those previously reported.[17]

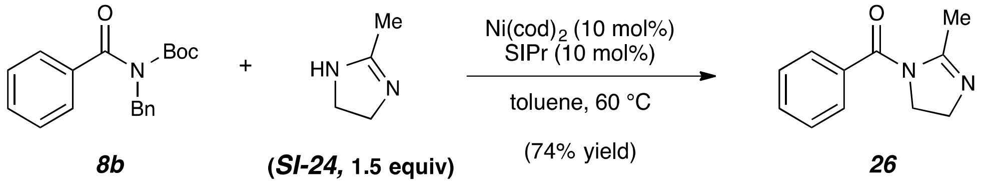


**8b** (**SI-24,** 1.5 equiv) **26**

**Amide 26 (Figure 3 entry 16)**. Purification by flash chromatography (1:1 Hexanes:EtOAc) generated amide **26** (74% yield, average of two experiments) as a pale yellow solid. Amide **26**: $R_f$ 0.21 (100% EtOAc) Spectral data match those previously reported.[18]

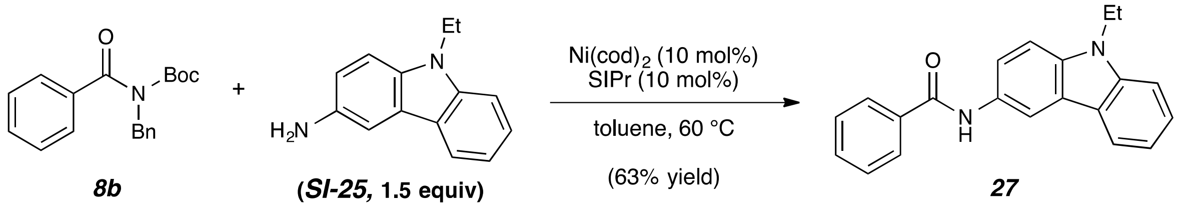


**8b** (**SI-25,** 1.5 equiv) **27**

**Amide 27 (Figure 3 entry 17)**. Purification by flash chromatography (17:1 Hexanes:EtOAc) generated amide **27** (63% yield, average of two experiments) as a tan solid. Amide **27**: $R_f$ 0.70 (1:1 Hexanes:EtOAc); $^1$H NMR (500 MHz, $CDCl_3$): δ 8.45 (s, 1H), 8.10 (m, 1H), 7.99–7.90 (m, 3H), 7.68–7.61 (m, 1H), 7.59–7.44 (m, 4H), 7.43–7.37 (m, 2H), 7.25–7.20 (m, 1H), 4.42–4.33 (m, 2H), 1.48–1.40 (m, 3H); $^{13}$C NMR (125 MHz, $CDCl_3$, 18 of 19 observed): δ 165.9, 140.6, 137.5, 135.4, 131.8, 129.8, 128.9, 127.2, 126.0, 123.2, 122.9, 120.9, 119.9, 118.9, 113.3, 108.7, 37.8, 14.0; IR (film): 3300, 3055, 2977, 1644, 1536 cm$^{-1}$; HRMS-ESI (*m/z*) $[M + H]^+$ calcd for $C_{21}H_{18}N_2O$, 315.1497; found 315.1477.

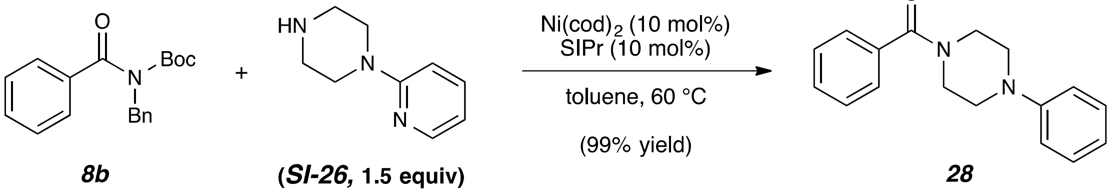


**8b** (**SI-26,** 1.5 equiv) **28**

**Amide 28 (Figure 3 entry 18)**. Purification by flash chromatography (100% EtOAc) generated amide **28** (99% yield, average of two experiments) as a yellow solid. Amide **28**: $R_f$ 0.48 (100% EtOAc) Spectral data match those previously reported.[19]

**Amino Ester Scope**

**Representative procedure for free-basing amino esters and subsequent reaction with substrate SI-7 (coupling of amide SI-7 and alanine *tert*-butyl ester (SI-27) is used as an example).**

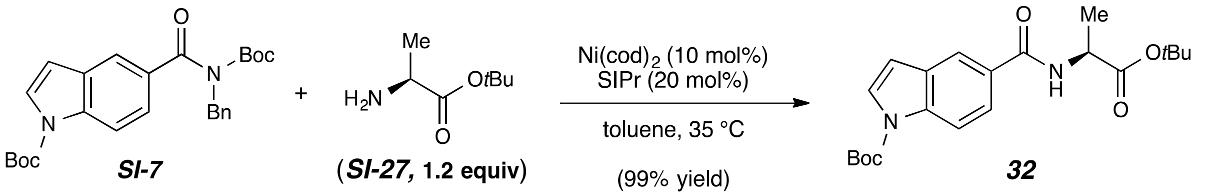


**Amide 32 (Figure 4 entry 1):** A 25 mL flask with a magnetic stir bar was flame-dried under reduced pressure, and then allowed to cool under $N_2$. The vial was charged with NH-Ala-O*t*Bu HCl (500 mg, 2.76 mmol, 1.0 equiv), and the vial was flushed with $N_2$. $CH_2Cl_2$ (14.5 mL, 0.19 M) and Amberlyst® A21 free base (1.0 g, 200 wt%) were added and the resulting mixture was stirred vigorously at 23 °C for 3 h. The mixture was then filtered over a plug of celite (15 mL of $CH_2Cl_2$). The volatiles were removed under reduced pressure to yield the free-based amino ester (80% yield), which was used directly in the nickel-catalyzed transamidation.

A 1-dram vial containing a magnetic stir bar was flame-dried under reduced pressure, and then allowed to cool under $N_2$. The vial was charged with substrate **SI-7** (90.1 mg, 0.200 mmol, 1.0 equiv) and the free-based NH-Ala-O*t*Bu (**SI-27**) (34.9 mg, 0.240 mmol, 1.2 equiv). The vial was flushed with $N_2$, then taken into a glove box, and charged with $Ni(cod)_2$ (5.5 mg, 0.020 mmol, 10 mol%) and SIPr (15.6 mg, 0.040 mmol, 20 mol%). Subsequently, toluene (0.20 mL, 1.0 M) was added. The vial was sealed with a Teflon-lined screw cap, removed from the glove box, and stirred at 35 °C for 14 h. After cooling to 23 °C, the mixture was diluted with hexanes (0.5 mL) and filtered over a plug of silica gel (10 mL of EtOAc eluent). The volatiles were removed under reduced pressure, and the crude residue was purified by preparative thin-layer chromatography (5:1 Hexanes:EtOAc) to yield amide product **32** (99% yield, average of two experiments) as an amorphous solid. Amide **32**: $R_f$ 0.15 (5:1 Hexanes:EtOAc); $^1H$ NMR (500

MHz, $CDCl_3$): δ 8.18 (d, *J* = 8.3, 1H), 8.06 (d, *J* = 1.48, 1H), 7.76 (dd, *J* = 8.7, 1.8, 1H), 7.64 (d, *J* = 3.6, 1H), 6.83 (d, J = 7.1, 1H), 6.62 (d, *J* = 3.6, 1H), 4.71 (app quintet, *J* = 7.1, 1H), 1.68 (s, 9H), 1.53–1.49 (m, 12H); $^{13}C$ NMR (125 MHz, $CDCl_3$): δ 172.9, 167.1, 149.6, 137.2, 130.6, 128.9, 127.3, 123.2, 120.5, 115.2, 107.8, 84.4, 82.3, 49.2, 28.3, 28.2, 19.2; IR (film): 3319, 2979, 2936, 1733, 1638 $cm^{-1}$; HRMS-ESI (*m/z*) $[M + H]^+$ calcd for $C_{21}H_{29}N_2O_5$, 389.2076; found 389.2041; $[\alpha]^{20.6}_D$ +2.60 ° (*c* = 1.00, $CHCl_3$).

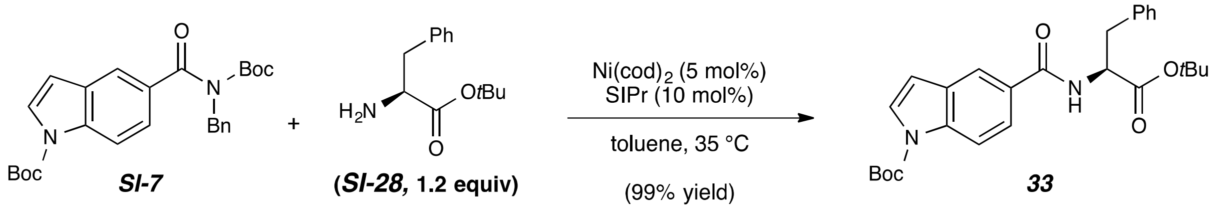


**Amide 33 (Figure 4 entry 2):** Purification by preparative thin-layer chromatography (25:1 → 5:1 Hexanes:EtOAc) generated amide **33** (99% yield, average of two experiments) as an amorphous solid. Amide **33**: $R_f$ 0.20 (5:1 Hexanes:EtOAc); $^1H$ NMR (500 MHz, $CDCl_3$): δ 8.17 (d, *J* = 8.3, 1H), 8.00 (d, *J* = 1.3, 1H), 7.69 (dd, *J* = 8.7, 1.8, 1H), 7.64 (d, *J* = 3.5, 1H), 7.36–7.16 (m, 5H), 6.69 (d, *J* = 7.3, 1H), 6.62 (dd, *J* = 3.7, 0.35, 1H), 5.05–4.93 (m, 1H), 3.33–3.16 (m, 2H), 1.68 (s, 9H), 1.44 (s, 9H); $^{13}C$ NMR (125 MHz, $CDCl_3$): δ 171.0, 167.1, 149.6, 137.2, 136.4, 130.6, 129.8, 128.8, 128.5, 127.3, 127.1, 123.1, 120.5, 115.2, 107.8, 84.4, 82.7, 54.1, 38.2, 28.3, 28.2; IR (film): 3320, 2979, 2934, 1733, 1642 $cm^{-1}$; HRMS-ESI (*m/z*) $[M + H]^+$ calcd for $C_{27}H_{33}N_2O_5$, 465.2389; found 465.2357; $[\alpha]^{20.9}_D$ +61.4 ° (*c* = 1.00, $CHCl_3$).

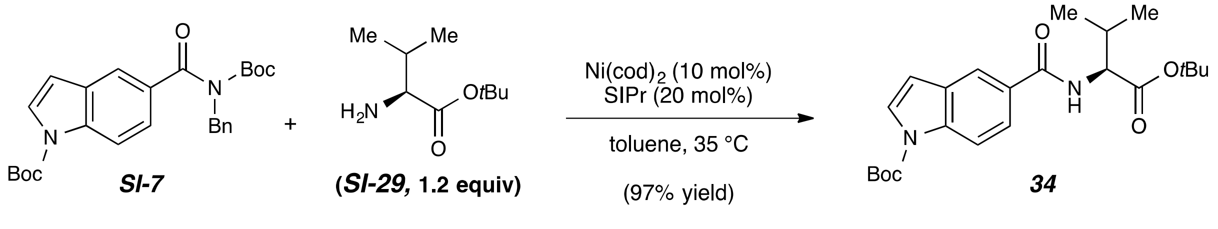


**Amide 34 (Figure 4 entry 3)**. Purification by preparative thin-layer chromatography (9:1 Hexanes:EtOAc) generated amide **34** (97% yield, average of two experiments) as an amorphous solid. Amide **34**: $R_f$ 0.40 (5:1 Hexanes:EtOAc); $^1H$ NMR (500 MHz, $CDCl_3$): δ 8.18 (d, *J* = 8.3,

1H), 8.06 (d, $J$ = 1.7, 1H), 7.77 (dd, $J$ = 8.7, 1.4, 1H), 7.64 (d, $J$ = 3.6, 1H), 6.72 (d, $J$ = 8.7, 1H), 6.63 (d, $J$ = 3.6, 1H), 4.72 (dd, $J$ = 8.3, 4.3, 1H), 2.37–2.21 (m, 1H), 1.68 (s, 9H), 1.50 (s, 9H), 1.02 (dd, $J$ = 10.7, 6.8, 6H); $^{13}$C NMR (125 MHz, $CDCl_3$): δ 171.7, 167.6, 149.6, 137.2, 130.6, 129.1, 127.3, 123.2, 120.5, 115.2, 107.8, 84.4, 82.3, 57.8, 32.0, 28.32, 28.25, 19.1, 18.0; IR (film): 3357, 2975, 2935, 1733, 1643 cm$^{-1}$; HRMS-ESI (*m/z*) $[M + H]^+$ calcd for $C_{23}H_{33}N_2O_5$, 417.2389; found 417.2350; $[\alpha]^{21.1}_D$ +176.8 ° (*c* = 1.00, $CHCl_3$).

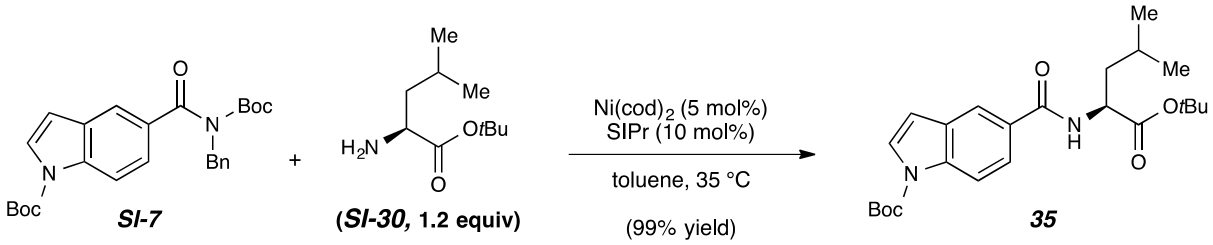


*SI-7* (*SI-30*, 1.2 equiv) **35**

**Amide 35 (Figure 4 entry 4)**. Purification by preparative thin-layer chromatography (10:1 → 5:1 Hexanes:EtOAc) generated amide **35** (99% yield, average of two experiments) as an amorphous solid. Amide **35**: $R_f$ 0.52 (3:1 Hexanes:EtOAc); $^1$H NMR (500 MHz, $CDCl_3$): δ 8.15 (d, $J$ = 8.0, 1H), 8.03 (d, $J$ = 1.6, 1H), 7.74 (dd, $J$ = 8.6, 1.6, 1H), 7.63 (d, $J$ = 3.5, 1H), 6.70 (d, $J$ = 8.6, 1H), 6.60 (d, $J$ = 3.5, 1H), 4.83–4.70 (m, 1H), 1.86–1.69 (m, 3H), 1.67 (s, 9H), 1.49 (s, 9H), 1.05–0.93 (m, 6H); $^{13}$C NMR (125 MHz, $CDCl_3$): δ 172.8, 167.4, 149.5, 137.1, 130.5, 128.9, 127.2, 123.2, 120.5, 115.1, 107.7, 84.3, 82.1, 51.9, 42.4, 28.3, 28.2, 25.2, 23.0, 22.4; IR (film): 3347, 2977, 2872, 1735, 1640 cm$^{-1}$; HRMS-ESI (*m/z*) $[M + H]^+$ calcd for $C_{24}H_{35}N_2O_5$, 431.2546; found 431.2506; $[\alpha]^{20.8}_D$ +27.0 ° (*c* = 1.00, $CHCl_3$).

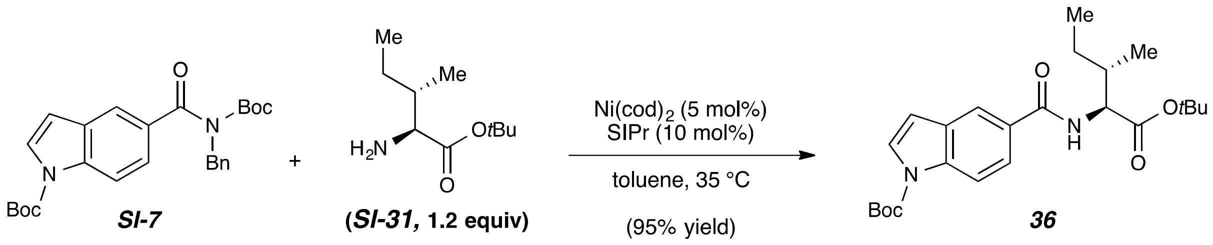


*SI-7* (*SI-31*, 1.2 equiv) **36**

**Amide 36 (Figure 4 entry 5)**. Purification by preparative thin-layer chromatography (10:1 → 5:1 Hexanes:EtOAc) generated amide **36** (95% yield, average of two experiments) as an amorphous solid. Amide **36**: $R_f$ 0.59 (3:1 Hexanes:EtOAc); $^1$H NMR (500 MHz, $CDCl_3$): δ 8.18 (d, $J$ = 8.2, 1H), 8.05 (d, $J$ = 1.5, 1H), 7.76 (dd, $J$ = 8.7, 1.5, 1H), 7.64 (d, $J$ = 3.5, 1H), 6.77 (d, $J$ = 8.7, 1H), 6.63 (dd, $J$ = 4.4, 0.5, 1H), 4.75 (dd, J = 8.2, 2.7, 1H), 2.11–1.94 (m, 1H), 1.68 (s, 9H), 1.64–1.54 (m, 1H), 1.50 (s, 9H), 1.38–1.20 (m, 1H), 1.05–0.94 (m, 6H); $^{13}$C NMR (125 MHz, $CDCl_3$): δ 171.5, 167.4, 149.5, 137.1, 130.6, 129.0, 127.3, 123.2, 120.4, 115.2, 107.7, 84.4, 82.3, 57.2, 38.7, 28.27, 28.23, 25.7, 15.5, 12.0; IR (film): 3356, 2974, 2935, 1738, 1644 cm$^{-1}$; HRMS-ESI (*m/z*) $[M + H]^+$ calcd for $C_{24}H_{35}N_2O_5$, 431.2546; found 431.2501; $[\alpha]^{21.7}_D$ +42.4 ° ($c$ = 1.00, $CHCl_3$).

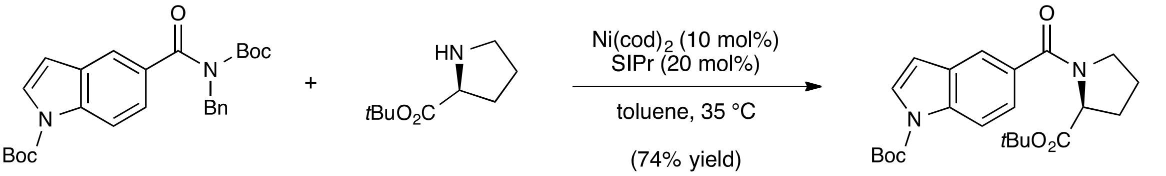


***SI-7*** (***SI-32,*** **1.2 equiv)** ***37***

**Amide 37 (Figure 4 entry 6)**. Purification by preparative thin-layer chromatography (5:1 Hexanes:EtOAc) generated amide **37** (74% yield, average of two experiments) as an amorphous solid. Amide **37**: $R_f$ 0.61 (1:1 Hexanes:EtOAc); $^1$H NMR (500 MHz, $CDCl_3$): δ 8.23–8.04 (m, 1H), 7.83–7.58 (m, 2H), 7.57–7.32 (m, 1H), 6.62–6.49 (m, 1H), 4.65–4.17 (m, 1H), 3.90–3.46 (m, 2H), 2.41–2.10 (m, 1H), 2.07–1.78 (m, 3H), 1.67 (s, 9H), 1.54–1.29 (m, 9H); $^{13}$C NMR (125 MHz, $CDCl_3$): δ 171.8, 171.1, 170.1, 149.7, 136.1, 131.7, 131.1, 130.2, 127.0, 123.7, 123.1, 120.6, 120.0, 115.1, 114.9, 107.6, 107.5, 84.2, 81.8, 81.4, 62.5, 60.2, 50.4, 46.8, 31.7, 29.6, 28.3, 28.2, 27.9, 25.6, 22.7; IR (film): 2978, 2935, 1733, 1407, 1366 cm$^{-1}$; HRMS-ESI (*m/z*) $[M + H]^+$ calcd for $C_{23}H_{31}N_2O_5$, 415.2233; found 415.1240; $[\alpha]^{21.4}_D$ +42.0 ° ($c$ = 1.00, $CHCl_3$). *Note:* ***37*** *was obtained as mixture of rotamers. These data represent empirically observed chemical shifts from the* $^{13}$*C NMR spectrum.*

**Verification of Enantiopurity - Racemic Compound Synthesis**

**Representative procedure for free-basing racemic amino esters and subsequent reaction with substrate SI-7 (coupling of amide SI-7 and rac-alanine *tert*-butyl ester (rac-SI-27) is used as an example).**

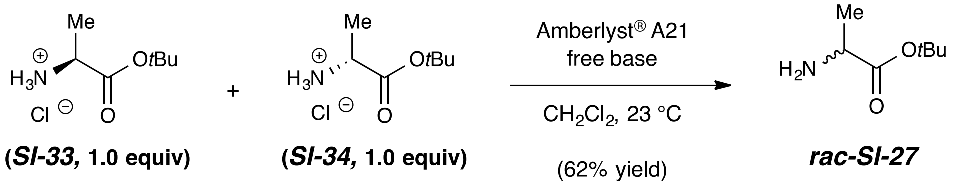


**Racemic Amino Ester rac-SI-27:** A 10 mL flask with a magnetic stir bar was flame-dried under reduced pressure, and then allowed to cool under $N_2$. The vial was charged with L-NH-Ala-O*t*Bu HCl (**SI-33**) (300 mg, 1.65 mmol, 1.0 equiv), and D-NH-Ala-O*t*Bu HCl (**SI-34**) (300 mg, 1.65 mmol, 1.0 equiv). The vial was flushed with $N_2$ and then $CH_2Cl_2$ (17.4 mL, 0.19 M), and Amberlyst® A21 free base (1.2 g, 200 wt%) were added. After stirring vigorously at 23 °C for 2 h, the mixture was filtered over a plug of celite (15 mL of $CH_2Cl_2$). The volatiles were removed under reduced pressure to yield the free-based amino ester **rac-SI-27** (62% yield).

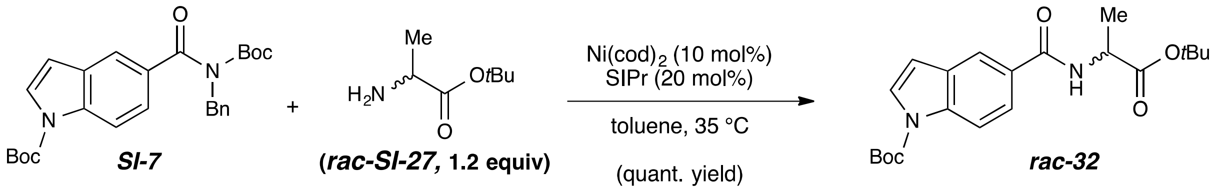


**Racemic Amide rac-32:** A 1-dram vial was flame-dried under reduced pressure, and then allowed to cool under $N_2$. The vial was charged with a magnetic stir bar, amide substrate **SI-7** (90.1 mg, 0.200 mmol, 1.0 equiv), and the free-based racemic NH-Ala-O*t*Bu (**rac-SI-27**) (34.8 mg, 0.240 mmol, 1.2 equiv). The vial was flushed with $N_2$, taken into a glove box, and charged with $Ni(cod)_2$ (5.5 mg, 0.020 mmol, 10 mol%) and SIPr (15.6 mg, 0.040 mmol, 20 mol%). Subsequently, toluene (0.20 mL, 1.0 M) was added. The vial was sealed with a Teflon-lined screw cap, removed from the glove box, and stirred at 35 °C for 14 h. After cooling to 23 °C, the mixture was diluted with hexanes (0.5 mL) and filtered over a plug of silica gel (10 mL of EtOAc eluent). The volatiles were removed under reduced pressure, and the crude residue was

purified by preparative thin-layer chromatography (4:1 Hexanes:EtOAc) to yield amide product **rac-32** (quant. yield) as an amorphous solid.

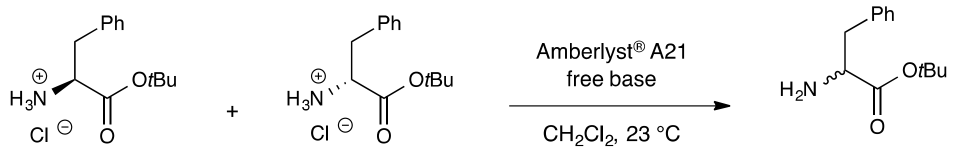


(*SI-35,* 1.0 equiv) (*SI-36,* 1.0 equiv) ***rac-SI-28***

**Racemic Amino Ester rac-SI-28:** A 10 mL flask with a magnetic stir bar was flame-dried under reduced pressure, and then allowed to cool under $N_2$. The vial was charged with L-NH-Phe-O*t*Bu HCl (**SI-35**) (50 mg, 0.194 mmol, 1.0 equiv), and D-NH-Phe-O*t*Bu HCl (**SI-36**) (50 mg, 0.194 mmol, 1.0 equiv). The vial was flushed with $N_2$ and then $CH_2Cl_2$ (2.0 mL, 0.19 M), and Amberlyst® A21 free base (0.200 g, 200 wt%) were added. After stirring vigorously at 23 °C for 2 h, the mixture was filtered over a plug of celite (15 mL of $CH_2Cl_2$). The volatiles were removed under reduced pressure to yield the free-based amino ester **rac-SI-28** (74% yield).

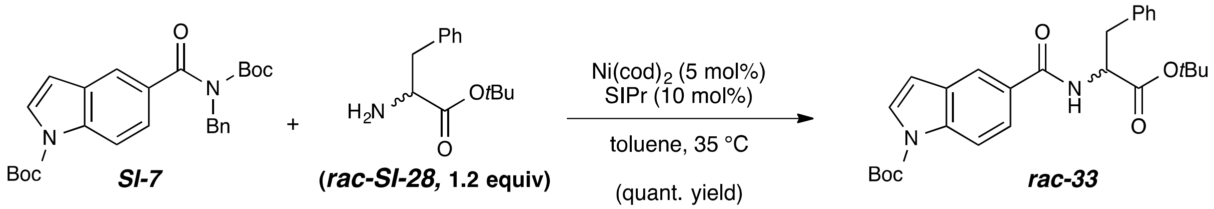


***SI-7*** (***rac-SI-28,*** 1.2 equiv) ***rac-33***

**Racemic Amide rac-33:** A 1-dram vial was flame-dried under reduced pressure, and then allowed to cool under $N_2$. The vial was charged with a magnetic stir bar, amide substrate **SI-7** (90.1 mg, 0.200 mmol, 1.0 equiv), and the free-based racemic NH-Phe-O*t*Bu (**rac-SI-28**) (53.1 mg, 0.240 mmol, 1.2 equiv). The vial was flushed with $N_2$, taken into a glove box, and charged with $Ni(cod)_2$ (2.8 mg, 0.010 mmol, 5 mol%) and SIPr (7.8 mg, 0.020 mmol, 10 mol%). Subsequently, toluene (0.20 mL, 1.0 M) was added. The vial was sealed with a Teflon-lined screw cap, removed from the glove box, and stirred at 35 °C for 14 h. After cooling to 23 °C, the mixture was diluted with hexanes (0.5 mL) and filtered over a plug of silica gel (10 mL of EtOAc eluent). The volatiles were removed under reduced pressure, and the crude residue was

purified by preparative thin-layer chromatography (5:1 Hexanes:EtOAc) to yield amide product **rac-33** (quant. yield) as an amorphous solid.

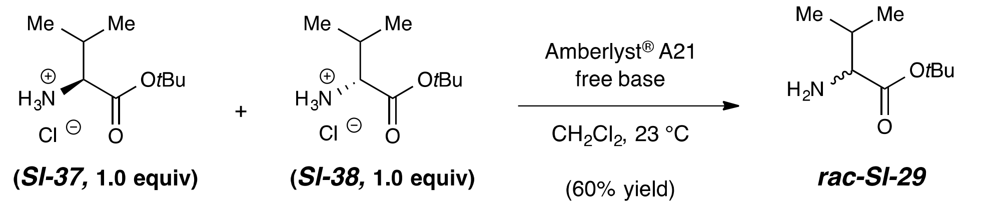


**Racemic Amino Ester rac-SI-29:** A 10 mL flask with a magnetic stir bar was flame-dried under reduced pressure, and then allowed to cool under $N_2$. The vial was charged with L-NH-Val-O*t*Bu HCl (**SI-37**) (300 mg, 1.43 mmol, 1.0 equiv), and D-NH-Val-O*t*Bu HCl (**SI-38**) (300 mg, 1.43 mmol, 1.0 equiv). The vial was flushed with $N_2$ and then $CH_2Cl_2$ (15.0 mL, 0.19 M), and Amberlyst® A21 free base (1.20 g, 200 wt%) were added. After stirring vigorously at 23 °C for 2 h, the mixture was filtered over a plug of celite (15 mL of $CH_2Cl_2$). The volatiles were removed under reduced pressure to yield the free-based amino ester **rac-SI-29** (60% yield).

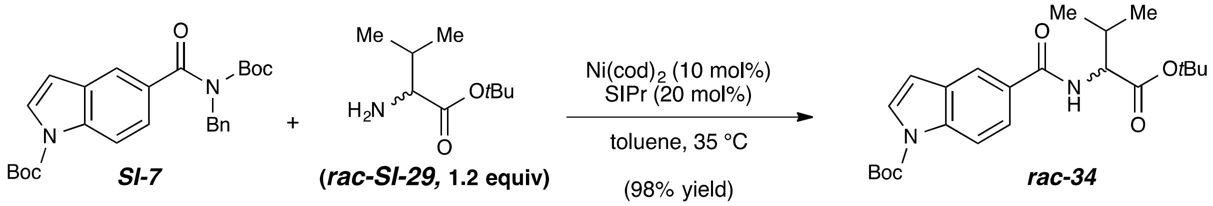


**Racemic Amide rac-34:** A 1-dram vial was flame-dried under reduced pressure, and then allowed to cool under $N_2$. The vial was charged with a magnetic stir bar, amide substrate **SI-7** (90.1 mg, 0.200 mmol, 1.0 equiv), and the free-based racemic NH-Val-O*t*Bu (**rac-SI-29**) (41.6 mg, 0.240 mmol, 1.2 equiv). The vial was flushed with $N_2$, taken into a glove box, and charged with $Ni(cod)_2$ (5.5 mg, 0.020 mmol, 10 mol%) and SIPr (15.6 mg, 0.040 mmol, 20 mol%). Subsequently, toluene (0.20 mL, 1.0 M) was added. The vial was sealed with a Teflon-lined screw cap, removed from the glove box, and stirred at 35 °C for 14 h. After cooling to 23 °C, the mixture was diluted with hexanes (0.5 mL) and filtered over a plug of silica gel (10 mL of EtOAc eluent). The volatiles were removed under reduced pressure, and the crude residue was

purified by preparative thin-layer chromatography (4:1 Hexanes:EtOAc 2X) to yield amide product **rac-34** (98% yield) as an amorphous solid.

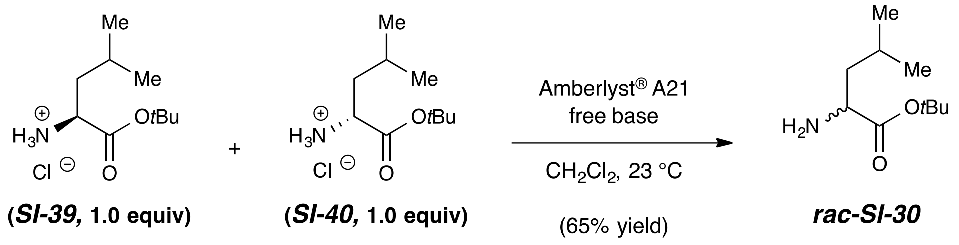


**Racemic Amino Ester rac-SI-30:** A 10 mL flask with a magnetic stir bar was flame-dried under reduced pressure, and then allowed to cool under $N_2$. The vial was charged with L-NH-Leu-O*t*Bu HCl (**SI-39**) (300 mg, 1.34 mmol, 1.0 equiv), and D-NH-Leu-O*t*Bu HCl (**SI-40**) (300 mg, 1.34 mmol, 1.0 equiv). The vial was flushed with $N_2$ and then $CH_2Cl_2$ (14.1 mL, 0.19 M), and Amberlyst® A21 free base (1.20 g, 200 wt%) were added. After stirring vigorously at 23 °C for 2 h, the mixture was filtered over a plug of celite (15 mL of $CH_2Cl_2$). The volatiles were removed under reduced pressure to yield the free-based amino ester **rac-SI-30** (65% yield).

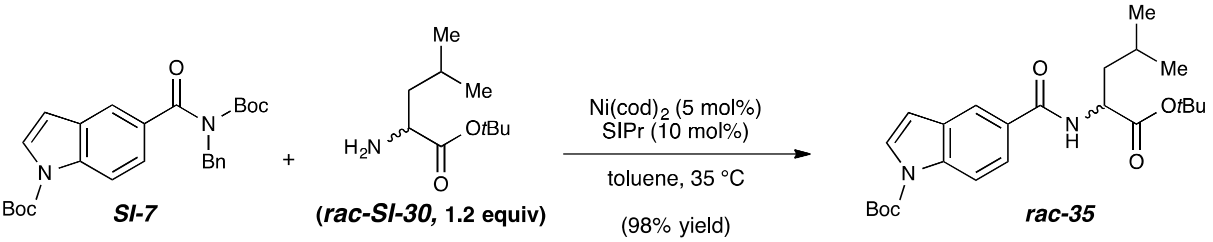


**Racemic Amide rac-35:** A 1-dram vial was flame-dried under reduced pressure, and then allowed to cool under $N_2$. The vial was charged with a magnetic stir bar, amide substrate **SI-7** (90.1 mg, 0.200 mmol, 1.0 equiv), and the free-based racemic NH-Leu-O*t*Bu (**rac-SI-30**) (45.0 mg, 0.240 mmol, 1.2 equiv). The vial was flushed with $N_2$, taken into a glove box, and charged with Ni(cod)$_2$ (2.8 mg, 0.010 mmol, 5 mol%) and SIPr (7.8 mg, 0.020 mmol, 10 mol%). Subsequently, toluene (0.20 mL, 1.0 M) was added. The vial was sealed with a Teflon-lined screw cap, removed from the glove box, and stirred at 35 °C for 14 h. After cooling to 23 °C, the mixture was diluted with hexanes (0.5 mL) and filtered over a plug of silica gel (10 mL of

EtOAc eluent). The volatiles were removed under reduced pressure, and the crude residue was purified by preparative thin-layer chromatography (4:1 Hexanes:EtOAc 2X) to yield amide product **rac-35** (98% yield) as an amorphous solid.

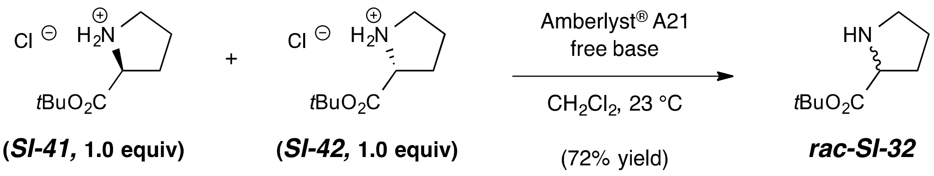


**Racemic Amino Ester rac-SI-32:** A 10 mL flask with a magnetic stir bar was flame-dried under reduced pressure, and then allowed to cool under $N_2$. The vial was charged with L-NH-Pro-O*t*Bu HCl (**SI-41**) (300 mg, 1.44 mmol, 1.0 equiv), and D-NH-Pro-O*t*Bu HCl (**SI-42**) (300 mg, 1.44 mmol, 1.0 equiv). The vial was flushed with $N_2$ and then $CH_2Cl_2$ (15.2 mL, 0.19 M), and Amberlyst® A21 free base (1.20 g, 200 wt%) were added. After stirring vigorously at 23 °C for 2 h, the mixture was filtered over a plug of celite (15 mL of $CH_2Cl_2$). The volatiles were removed under reduced pressure to yield the free-based amino ester **rac-SI-32** (72% yield).

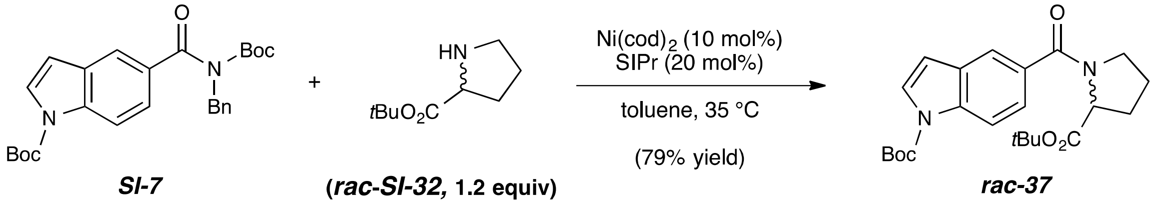


**Racemic Amide rac-37:** A 1-dram vial was flame-dried under reduced pressure, and then allowed to cool under $N_2$. The vial was charged with a magnetic stir bar, amide substrate **SI-7** (90.1 mg, 0.200 mmol, 1.0 equiv), and the free-based racemic NH-Pro-O*t*Bu (**rac-SI-32**) (41.1 mg, 0.240 mmol, 1.2 equiv). The vial was flushed with $N_2$, taken into a glove box, and charged with Ni(cod)$_2$ (5.5 mg, 0.020 mmol, 10 mol%) and SIPr (15.6 mg, 0.040 mmol, 20 mol%). Subsequently, toluene (0.20 mL, 1.0 M) was added. The vial was sealed with a Teflon-lined screw cap, removed from the glove box, and stirred at 35 °C for 14 h. After cooling to 23 °C, the mixture was diluted with hexanes (0.5 mL) and filtered over a plug of silica gel (10 mL of EtOAc eluent). The volatiles were removed under reduced pressure, and the crude residue was

purified by preparative thin-layer chromatography (6:1 Hexanes:EtOAc) to yield amide product **rac-37** (79% yield) as an amorphous solid.

**Supplementary References**


[1] Weires, N. A.; Baker, E. L. & Garg, N. K. Nickel-catalysed Suzuki-Miyaura coupling of amides. *Nat. Chem.* **8**, 75–79 (2016).

[2] Zhang, N.; Li, B.; Zhong, H.; Huang, J. Synthesis of *N*-alkyl and *N*-aryl isoquinolones and derivatives via Pd-catalysed C–H activation and cyclization reactions. *Org. Biomol. Chem.* **10**, 9429–9439 (2012).

[3] Li, Y.; Jia, F.; Li, Z. Iron-catalyzed oxidative amidation of tertiary amines with aldehydes. *Chem. Eur. J.* **19**, 82–86 (2013).

[4] Oishi, S.; Saito, S. Double molecular recognition with aminoorganoboron complexes: Selective alcoholysis of β-dicarbonyl derivatives. *Angew. Chem Int. Ed.* **51**, 5395–5399 (2012).

[5] De Koning, C. B.; Michael, J. P.; Rousseau, A. L. A versatile and convenient method for the synthesis of substituted benzo[*a*]carbazoles and pyrido[2,3-*a*]carbazoles. *J. Chem. Soc., Perkin Trans. 1*, 1705–1713 (2000).

[6] Verma, S. K.; Ghorpade, R.; Pratap, A.; Kaushik, M. P. Solvent free, *N*,*N*'-carbonyldiimidazole (CDI) mediated amidation. *Tetrahedron Lett.* **53**, 2373–2376 (2012).

[7] Tobisu, M.; Shimasaki, T.; Chatani, N. $Ni^0$-catalyzed direct amination of anisoles involving the cleavage of carbon–oxygen bonds. *Chem. Lett.* **38**, 710–711 (2009).

[8] Li, J.; Xu, F.; Zhang, Y.; Shen, Q. Heterobimetallic lanthanide/sodium phenoxides: Efficient catalysts for amidation of aldehydes with amines. *J. Org. Chem.* **74**, 2575–2577 (2009).

[9] Fang, W.; Deng, Q.; Xu, M.; Tu, T. Highly efficient aminocarbonylation of iodoarenes at atmospheric pressure catalyzed by a robust acenaphthoimidazolyidene allylic palladium complex. *Org. Lett.* **15**, 3678–3681 (2013).

[10] Chen, M.; Buchwald, S. L. Rapid and efficient trifluoromethylation of aromatic and heteroaromatic compounds using potassium trifluoroacetate enabled by a flow system. *Angew. Chem. Int. Ed.* **52**, 11628–11631 (2013).

[11] Lysén, M.; Kelleher, S.; Begtrup, M.; Kristensen, J. L. Synthesis of tertiary benzamides via Pd-catalyzed coupling of arylboronic esters and carbamoyl chlorides. *J. Org. Chem.* **70**, 5342–5343 (2005).

[12] Zanatta, N.; Faoro, D.; Silva, S. C.; Bonacorso, H. G.; Martins, M. A. P. Convenient synthesis of furan-3-carboxylic acid and derivatives. *Tetrahedron Lett.* **45**, 5689–5691 (2004).

[13] Friis, S. D.; Skrydstrup, T.; Buchwald, S. L. Mild Pd-catalyzed aminocarbonylation of (hetero)aryl bromides with a palladacycle precatalyst. *Org. Lett.* **16**, 4296–4299 (2014).

[14] Teo, Y.-C.; Yong, F.-F.; Ithnin, I. K.; Yio, S.-H. T.; Lin, Z. Efficient manganese/copper bimetallic catalyst for N-arylation of amides and sulfonamides under mild conditions in water. *Eur. J. Org. Chem.* 515–524 (2015).

[15] Yang, S.; Li, B.; Wan, X.; Shi, Z. Ortho arylation of acetanilides via Pd(II)-catalyzed C–H functionalization. *J. Am. Chem. Soc.* **129**, 6066–6067 (2007).

[16] Quesnel, J. S.; Arndtsen, B. A. A palladium-catalyzed carbonylation approach to acid chloride synthesis. *J. Am. Chem. Soc.* **135**, 16841–16844 (2013).

[17] Dokli, I.; Gredičak, M. Mechanochemical ritter reaction: A rapid approach to functionalized amides at room temperature. *Eur. J. Org. Chem.* 2727–2732 (2015).

[18] Ye, G.; Chen, C.; Chatterjee, S.; Collier, W. E.; Zhou, A.; Song, Y.; Beard, D. J.; Jr. Pittman, C. U. Tandem reactions of 2-methylimidazoline and 1,2-dimethylimidazoline with various benzoyl chlorides. *Synthesis* 141–152 (2010).

[19] Sarswat, A.; Kumar, R.; Kumar, L.; Lal, N.; Sharma, S.; Prabhakar, Y. S.; Pandey, S. K.; Lal, J.; Verma, V.; Jain, A.; Maikhuri, J. P.; Dalela, D.; Gupta, G.; Sharma, V. L. Arylpiperazines for management of benign prostatic hyperplasia: Design, synthesis, quantitative structure–activity relationships, and pharmacokinetic studies. *J. Med. Chem.* **54**, 302–311 (2011).
